# Comparative genomic analysis of the aldehyde dehydrogenase gene superfamily in *Arabidopsis thaliana* – searching for the functional key to hypoxia tolerance

**DOI:** 10.3389/fpls.2022.1000024

**Published:** 2022-11-17

**Authors:** Yufeng Guan, Umesh Kumar Tanwar, Ewa Sobieszczuk-Nowicka, Jolanta Floryszak-Wieczorek, Magdalena Arasimowicz-Jelonek

**Affiliations:** ^1^ Department of Plant Ecophysiology, Faculty of Biology, Adam Mickiewicz University in Poznań, Poznań, Poland; ^2^ Department of Plant Physiology, Faculty of Biology, Adam Mickiewicz University in Poznań, Poznań, Poland; ^3^ Department of Plant Physiology, Poznań University of Life Sciences, Poznań, Poland

**Keywords:** aldehyde dehydrogenases, hypoxia, flooding, abiotic stresses, arabidopsis

## Abstract

Flooding entails different stressful conditions leading to low oxygen availability for respiration and as a result plants experience hypoxia. Stress imposed by hypoxia affects cellular metabolism, including the formation of toxic metabolites that dramatically reduce crop productivity. Aldehyde dehydrogenases (ALDHs) are a group of enzymes participating in various aspects of plant growth, development and stress responses. Although we have knowledge concerning the multiple functionalities of ALDHs in tolerance to various stresses, the engagement of ALDH in plant metabolism adjustment to hypoxia is poorly recognized. Therefore, we explored the *ALDH* gene superfamily in the model plant *Arabidopsis thaliana*. Genome-wide analyses revealed that 16 *AtALDH* genes are organized into ten families and distributed irregularly across *Arabidopsis* 5 chromosomes. According to evolutionary relationship studies from different plant species, the *ALDH* gene superfamily is highly conserved. *AtALDH2* and *ALDH3* are the most numerous families in plants, while *ALDH18* was found to be the most distantly related. The analysis of *cis*-acting elements in promoters of *AtALDHs* indicated that *AtALDHs* participate in responses to light, phytohormones and abiotic stresses. Expression profile analysis derived from qRT-PCR showed the *AtALDH2B7*, *AtALDH3H1* and *AtALDH5F1* genes as the most responsive to hypoxia stress. In addition, the expression of *AtALDH18B1*, *AtALDH18B2*, *AtALDH2B4*, and *AtALDH10A8* was highly altered during the post-hypoxia-reoxygenation phase. Taken together, we provide comprehensive functional information on the *ALDH* gene superfamily in Arabidopsis during hypoxia stress and highlight *ALDHs* as a functional element of hypoxic systemic responses. These findings might help develop a framework for application in the genetic improvement of crop plants.

## Introduction

Aldehyde dehydrogenases (ALDHs) are a group of ubiquitous enzymes involved in metabolism of both prokaryotes and eukaryotes. They can oxidize excess of aliphatic and aromatic aldehydes that are intermediates or byproducts of crucial biochemical pathways in living cells (Kirch et al., 2004; Stiti et al., 2011; Brocker et al., 2013). The enzyme catalyzes the conversion of reactive aldehydes to the corresponding non-toxic carboxylic acids with the participation of NAD(P)^+^. Thus, the ALDH-mediated detoxification of aldehydes may attenuate reactive compounds that provide reactive carbonyl groups, which directly interact with DNA to cause severe changes and in consequence, lead to programmed cell death. Plants and animals have many common ALDH families and many genes are highly conserved between these two evolutionarily distinct groups (Brocker et al., 2013). ALDH was shown to play an essential role in carnitine biosynthesis, gluconeogenesis, glycolysis, amino acid metabolism and other physiological processes (Marchitti et al., 2008; Tylichová et al., 2010).

In plants *ALDHs* represent a widespread expression that underscores their multifaceted physiological roles, which are still largely unclear, and unraveling them is currently a priority of many scientific investigations (Guo et al., 2020; Tola et al., 2021; Islam and Ghosh, 2022). ALDH proteins are found under both physiological and pathophysiological conditions in different subcellular compartments, including cytosol, mitochondria, chloroplasts, peroxisomes and microsomes (Mitsuya et al., 2009; Missihoun et al., 2011) (Huang et al., 2008; Pawłowski et al., 2017; Guo et al., 2020). The multifaceted role of ALDHs has been well documented in rice. The aldehyde detoxification activity of rice OsALDH7 was proven to maintain seed viability (Shin et al., 2009; Shen et al., 2012), while OsALDH2b was found to negatively regulate tapetal programmed cell death and be essential for male reproductive development (Xie et al., 2020). In turn, OsALDH2a potentially functions in submergence tolerance (Nakazono et al., 2000).

Most plant *ALDH* genes studied so far are expressed under adverse environmental conditions accompanied by oxidative stress. Thus, the ALDH-dependent scavenging activity against toxic aldehydes derived from lipid peroxidation fulfills a cytoprotective role. Induction of ALDH at various gene expression levels was documented during a wide range of stress stimuli, including heat, ultraviolet radiation, dehydration, salinity, heavy metal or aluminum stresses, excessive light exposure or anaerobic conditions (Sunkar et al., 2003; Kotchoni et al., 2006). The stress-inducible ALDH proteins are predicted to be necessary for the mechanisms of stress metabolism adjustment and long-term adaptation, since the activity can alleviate the effects of oxidative stress (Stiti et al., 2011). This could not only be due to direct aldehyde detoxification activity, but also to the ALDH contribution to the homeostasis of pyridine nucleotides which are important redox sensors in cells. As indicated by Missihoun et al. (2018), the loss of function of Arabidopsis ALDH3I1 and ALDH7B4 led to a decrease in NAD(P)H, the NAD(P)H/NAD(P) ratio and an alteration of the glutathione pools affecting the efficiency of photosynthesis in Arabidopsis. It is known that reducing equivalents such as NAD(P)H and reduced glutathione are essential for maintaining cellular redox homeostasis (Xiao and Loscalzo, 2020). Thus, widespread ALDHs may participate in stress-induced dynamic redox changes.

Flooding as one of the most serious abiotic stresses affects plant cellular metabolism leading to a dramatic reduction in crop productivity. It restricts seed germination, plant growth and development at all stages of the life cycle; however, the developmental stage of the stressed plant and its duration are crucial for the scale and extent of damage (Fukao and Bailey-Serres, 2004; León et al., 2021). The adverse effects on plant organisms may vary depending on the flooding conditions that may affect only roots (waterlogging) or both roots and shoots (partial or complete submergence) (Sasidharan et al., 2017). Plants that experience flooding undergo hypoxia until the water subsides and normoxia conditions are restored. Plant responses induced by molecular-oxygen deficiency involve changes at the transcriptomic, proteomic, metabolomic and enzyme activity levels (e.g. León et al., 2021; Wang and Komatsu, 2022). Although the root is the first organ that senses hypoxia caused by floods, a set of physiological and biochemical changes is also provoked at the leaf level (Wang and Komatsu, 2022). This is confirmed by the fact that a reduction of available oxygen results in the limited net photosynthetic rate, diminished photosynthetic electron transport rate and photosystem II photochemical efficiency, alike the inhibition of transport from roots to leaves (Liu et al., 2014).

Plants that survive or undergo transient flooding reprogram their metabolism from the hypoxia phase to post-hypoxia reoxygenation, which is accompanied by an increased generation of reactive oxygen species (ROS) creating an oxidative cellular environment (Jethva et al., 2022; Zafari et al., 2022). ALDH is recognized as an aldehyde scavenger that eliminates toxic aldehydes caused by oxidative stress, while its implication in cellular response to hypoxia was evidenced in organisms belonging to various domains of life (Mustroph et al., 2009; Hermes-Lima et al., 2015; Sun et al., 2017). However, identification of the precise participation of plant ALDH in the unique metabolic switch induced by hypoxia still requires detailed research.

In the present study, we provided a detailed genome-wide identification, comprehensive gene description, evolution and expression analysis of the *ALDH* gene families in *Arabidopsis thaliana*. By integrating these data, we uncovered the importance of ubiquitous ALDHs in molecular-oxygen deficiency conditions created by flooding. To better understand the function of *AtALDHs* under hypoxia and post-hypoxia recovery, its expression patterns were examined by semi-quantitative RT-PCR. Although ALDHs of many species including *Arabidopsis thaliana* have been well characterized (Estey et al., 2007; Gao and Han, 2009; Jimenez-Lopez et al., 2010; Shen et al., 2012; Missihoun et al., 2018), this work contributes to more focused research and applications of these multifunctional enzymes in plants exposed to flooding. It highlights *ALDHs* as a functional element of hypoxic systemic responses, and identifies *ALDH* candidates involved in a metabolic switch induced by hypoxia and post-hypoxia conditions.

## Methods and materials:

### Characterization and phylogenetic analysis of the ALDH superfamily in *A. thaliana*

To retrieve the AtALDH superfamily gene and protein sequences the TAIR (https://www.arabidopsis.org/index.jsp), NCBI (https://www.ncbi.nlm.nih.gov/) and Sol genomics network (https://solgenomics.net/) databases were used, with the obtained sequences confirmed by the Hidden Markov Model (HMM). The AtALDH gene and protein nomenclature was adopted from the TAIR database and a previous study by Kirch et al. (2004). The presence of AtALDHs glutamic acid and cysteine activity sites was confirmed using PROSITE (https://prosite.expasy.org/). Multiple sequence alignments of 163 AtALDH protein sequences from selected crop plants: *Arabidopsis thaliana*(16 proteins), *Oryza sativa* (20 proteins), *Glycine max* (53 proteins), *Solanum lycopersicum* (29 proteins), *Solanum tuberosum* (22 proteins) and *Zea mays* (23 proteins) were performed using Clustal-W with default parameters in Clusal Omega (https://www.ebi.ac.uk/Tools/msa). MEGA7’s Maximum-likelihood method with 1000 bootstrap replicates was used to calculate genetic distances and construct the phylogenetic tree, while the obtained results were visualized by iTOL (https://itol.embl.de/).

### Chromosomal localization, collinearity analysis, gene structure and protein sequence analyses

The genome annotation file (GTF) containing the locations of the *AtALDH* genes in the genome and their structural information was extracted from the EnsemblPlants database (https://plants.ensembl.org/index.html) and TBtools (https://github.com/CJ-Chen/TBtools/releases) was used with default parameters to localize *AtALDHs* in chromosomal regions. Gene duplication events were analyzed using the Multiple Collinearity Scan toolkit (MCScanX) in TBtools with the default parameters. For the syntenic relationship between Arabidopsis and other plant species (*O. sativa, G. max, S. lycopersicum*, *S. tuberosum* and *Z. mays*) *ALDHs*, One step MCScanX toolkit analysis was carried out and visualized using the Dual Synsteny Plot in TBtools. Synonymous rate (d_S_), non-synonymous rate (d_N_), and evolutionary constraint (d_N_/d_S_) were calculated using the MEGA7 codon-based Z-test of selection (https://www.megasoftware.net/). The gene structure (intron/exon organization) of the *AtALDH* gene family was displayed by the Gene Structure Displayer Server 2.0 (http://gsds.gao-lab.org/). Conserved motifs in the protein sequences were predicted using the Multiple Expectation Maximization for Motif Elicitation program (MEME https://meme-suite.org/), with the number of motifs set at 10, while the distribution of motifs was based on “zero or one occurrence per sequence (zoops)”. Pfam (http://pfam.xfam.org/) and NCBI CDD (https://www.ncbi.nlm.nih.gov/Structure/cdd/cdd.shtml) were applied for AtALDH proteins to examine protein conserved domains and TBtools visualized obtained results.

### Analysis of *cis*-acting regulatory elements in the promoter region of the *AtALDH* genes

The 1500 bp genomic DNA sequence upstream of the initiation codon (ATG) was extracted from the Eukaryotic Promoter Database (https://epd.epfl.ch/), while the obtained sequences were then submitted to the PLACE database (http://www.dna.affrc.go.jp/PLACE/signalscan.html) and PlantCARE database (https://bioinformatics.psb.ugent.be/webtools/plantcare/html/) to search for potential *cis*-acting regulatory elements in the promoter regions of the *AtALDH* genes.

### Functional characterization analysis of *AtALDH* genes

Gene symbols of the *AtALDH* genes were submitted to the DAVID Bioinformatics Resources (https://david.ncifcrf.gov/home.jsp) with the default parameter and the obtained results were visualized by Hiplot (https://hiplot.com.cn).

### Gene expression profile during development stage and hypoxia stress of *AtALDHs*

*AtALDH* gene expression data during development were extracted from the developmental map of the Arabidopsis eFP Browser (https://www.bioinformatics.nl/efp/cgi-bin/efpWeb.cgi). The gene expression data were downloaded as expression levels, followed by calculation with the log 10 value. The expression levels of *AtALDHs* were visualized using a heatmap in TBtools. For the gene expression profiles of *AtALDHs* under hypoxia treatments, array- and sequence-based datasets were obtained from the NCBI-GEO repository (https://www.ncbi.nlm.nih.gov/geo/) and analyzed with GEO2R with default parameters. The heatmap diagram was constructed with logFC values using TBtools.

### Plant material, hypoxia stress and recovery procedure

The Arabidopsis ecotype Col-0 was used in this study. Seeds were sterilized with 1% sodium hypochlorite for 5 minutes and then rinsed at least 3 times with sterile water. The seeds were sown on peat pellets and incubated at 4°C for 72 hours in dark. Further plant growth was performed in a climate chamber (Binder, Germany) with a photoperiod of 16 h, temperature 23°C, relative humidity 70%, light intensity 180 μmol × m^-2^ × s^-1^ until ready for use.

The 4-week-old plants were submerged in sterile water tanks approximately 1 cm above peat pellets to assess expression levels of *AtALDHs* under hypoxic conditions, whereas untreated plants served as blank controls (0h). Arabidopsis leaves were collected at 24, 48 and 72 hours after hypoxia treatment and subsequently stored at -80°C. Further, plants that had undergone 24h hypoxia were reoxygenated to normal conditions, and samples were collected at 24 and 48 hours to analyze the gene expression during the recovery phase after hypoxia treatment.

### RNA isolation, reverse transcription and qRT-PCR gene expression analysis

According to the user’s guide, total RNA from Arabidopsis leaves was extracted using the TRI reagent (Sigma-Aldrich, USA). The RevertAid First-Strand cDNA Synthesis Kit (Thermo Scientific, USA) was used to synthesize first strand cDNA. Primers for the *AtALDH* gene superfamily were designed by Primer Blast (https://www.ncbi.nlm.nih.gov/tools/primer-blast/), each primer sequence was tested with a blast search in the genome of Arabidopsis for specific hits and for its septicity to yield a single amplicon on 3% agarose gel. All the primers were synthesized commercially at Genomed (https://www.genomed.pl/) and listed in **Supplementary Table S2**. The SYBR Green PCR Master Mix (Applied Biosystems, USA) and the Quant Studio 3 Real-Time PCR system (Applied Biosystems, USA) were used to perform qRT-PCR. The reaction consisted of denaturation at 95 °C for 10 s, primer annealing at 56 °C for 20s and primer extension at 72°C for 30s. For the entire qRT-PCR reaction 55 cycles were performed. After the data were collected from the Quant Studio 3 Real-Time PCR system, the CT value was determined by the PCR Miner Program (Zhao and Fernald, 2005). The relative gene expression was calculated using the Pfaffl method (Pfaffl, 2001). Obtained results were calculated based on the reference gene *Actin2* (At3g18780). All the results were based on three biological replicates and three technical replicates. The analysis of variance was conducted and the least significant differences (LSDs) between means were determined using Tukey’s test at the level of significance α=0.05.

### Structural feature analysis and homology modeling of AtALDH proteins

To predict the secondary structure of AtALDH proteins, SOPMA (https://npsa-prabi.ibcp.fr/cgi-bin/npsa_automat.pl?page=/NPSA/npsa_sopma.html) was used with default parameters. N-glycosylation sites of AtALDH proteins were predicted by NetNGlyc-1.0-services (https://services.healthtech.dtu.dk/service.php?NetNGlyc-1.0). Three hypoxia responsive AtALDH proteins AtALDH2B7; AtALDH3H1; and AtALDH5F1 were selected for the homology modelling using suitable homologous templates from the PDB database (http://ncbi.nlm.nih.gov/). ALDH protein models were built by the top PDB closed template *via* the target-template input using the SWISS-MODEL of the ExPASy web server (https://swissmodel.expasy.org/). Additionally, the PROCHECK test was used to inspect the 3D structure of AtALDH proteins in the SAVES server (http://nihserver.mbi.ucla.edu/SAVES/).

## Results

### Genome-wide characterization of ALDH in *A. thaliana*

**Figure 1:**
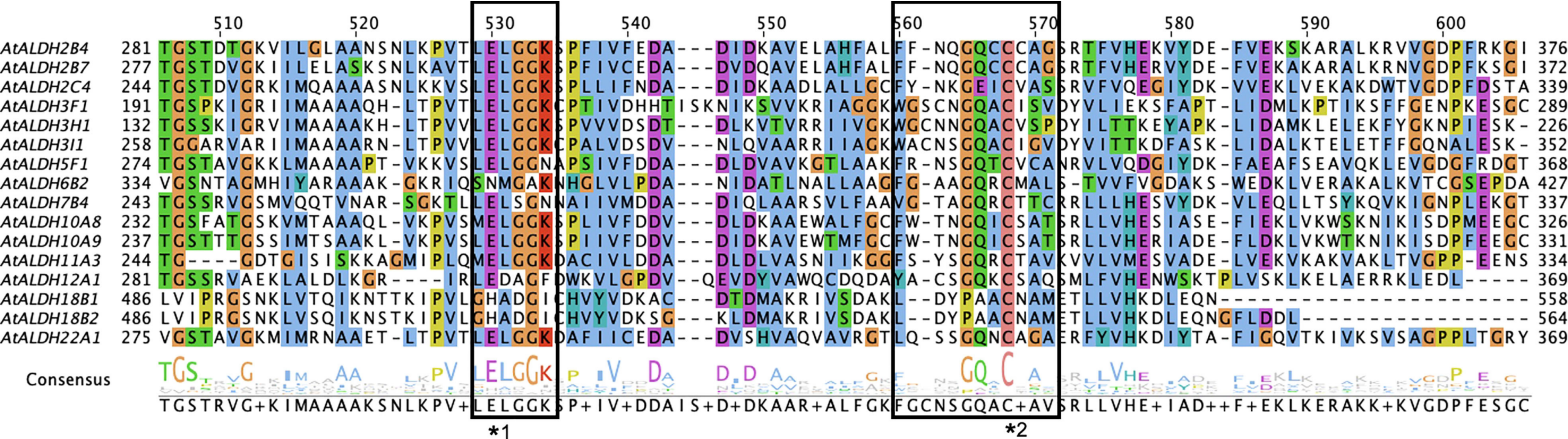
Multiple sequence alignments of the ALDH domain of all 16 AtALDH proteins. All 16 AtALDH protein sequences were analysed, the boxes represent conserved active sites for AtALDH proteins. *1 indicates a glutamic acid active site (PS00687) and *2 indicates a cysteine acid active site (PS00070).

In total, 16 members of the ALDH superfamily are present in *A. thaliana* belonging to ten subfamilies (ALDH2, 3, 5, 6, 7, 10, 11, 12, 18 and 22). Among these ten subfamilies, AtALDH2 and AtALDH3 are largest in number (with three members each), while AtALDH10 and AtALDH18 have two members each, followed by one member in the rest of the subfamilies. The distribution of cysteine and glutamic activity sites was determined by PROSITE (https://prosite.expasy.org/scanprosite/). Among the 16 members of AtALDH protein, both glutamic acid and cysteine acid active sites were present in 8 proteins (AtALDH2B4, AtALDH2B7, AtALDH2C4, AtALDH5F1, AtALDH10A8, AtALDH10A9, AtALDH11A3, At ALDH22A1), whereas 2 proteins (AtALDH3H1, AtALDH7B4) had only a glutamic active site and 3 proteins had only a cysteine active site (AtALDH3I1, AtALDH6B2, AtALDH12A1). Interestingly, no active sites were observed in AtALDH3F1, AtALDH18B1, and ALDH18B2 (**Figure 1**). The largest protein among the AtALDH superfamily was AtALDH18B2, which length was 726aa and molecular weight was 78.88 kDa. Conversely, the smallest protein in the AtALDH superfamily was ALDH3H1, with a length of 484aa and molecular weight of 53.16 kDa.

### Localization of *AtALDH* superfamily genes on chromosomes

**Figure 2:**
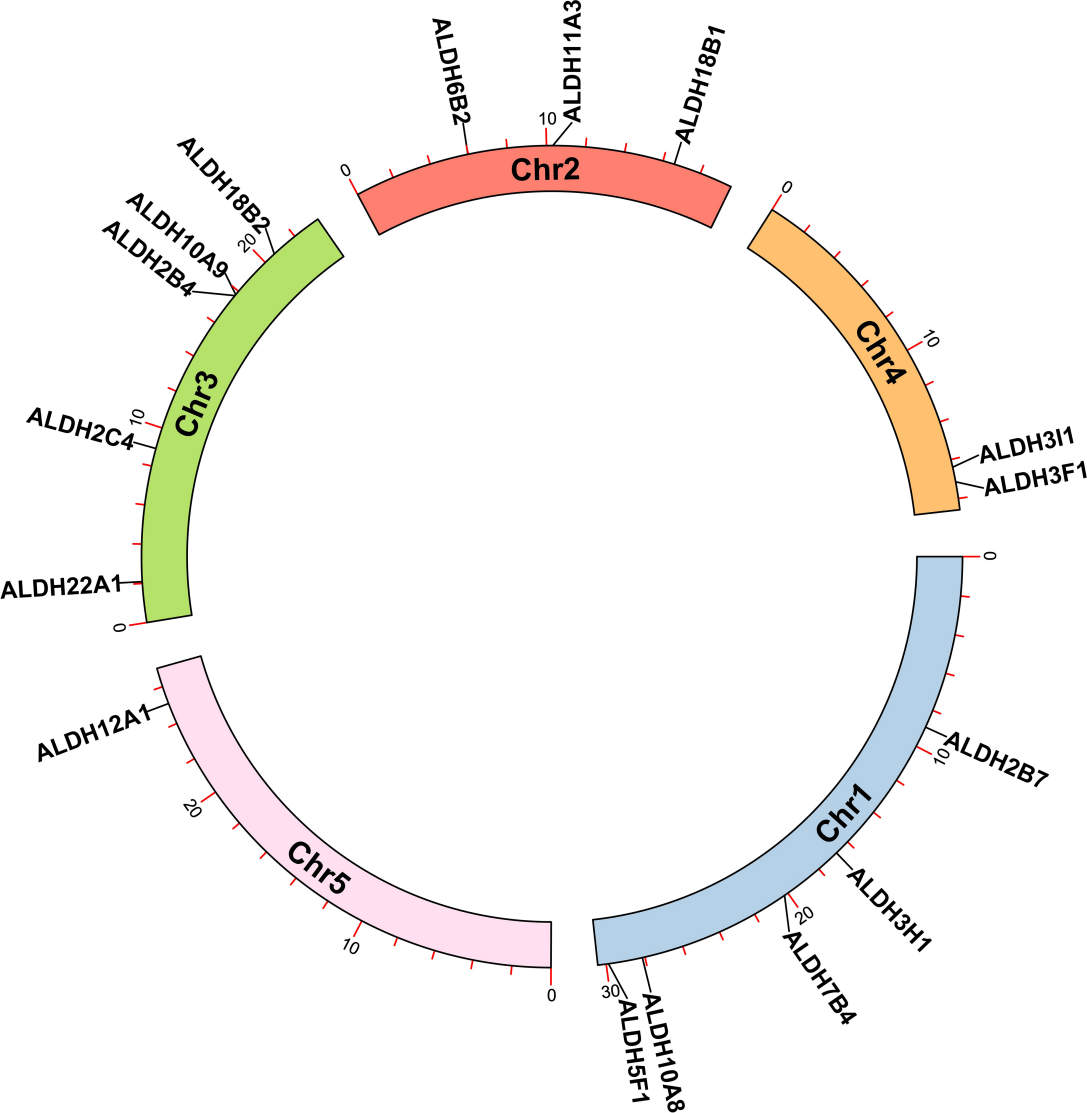
Chromosomal localization of *ALDH* genes in Arabidopsis. All 16 *AtALDH* genes were indicated on 5 different chromosomes of Arabidopsis with a black label. Arabidopsis chromosome length and *AtALDH* gene localization on chromosomes were derived from Ensemblplants (http://plants.ensembl.org/index.html).

**Figure 3:**
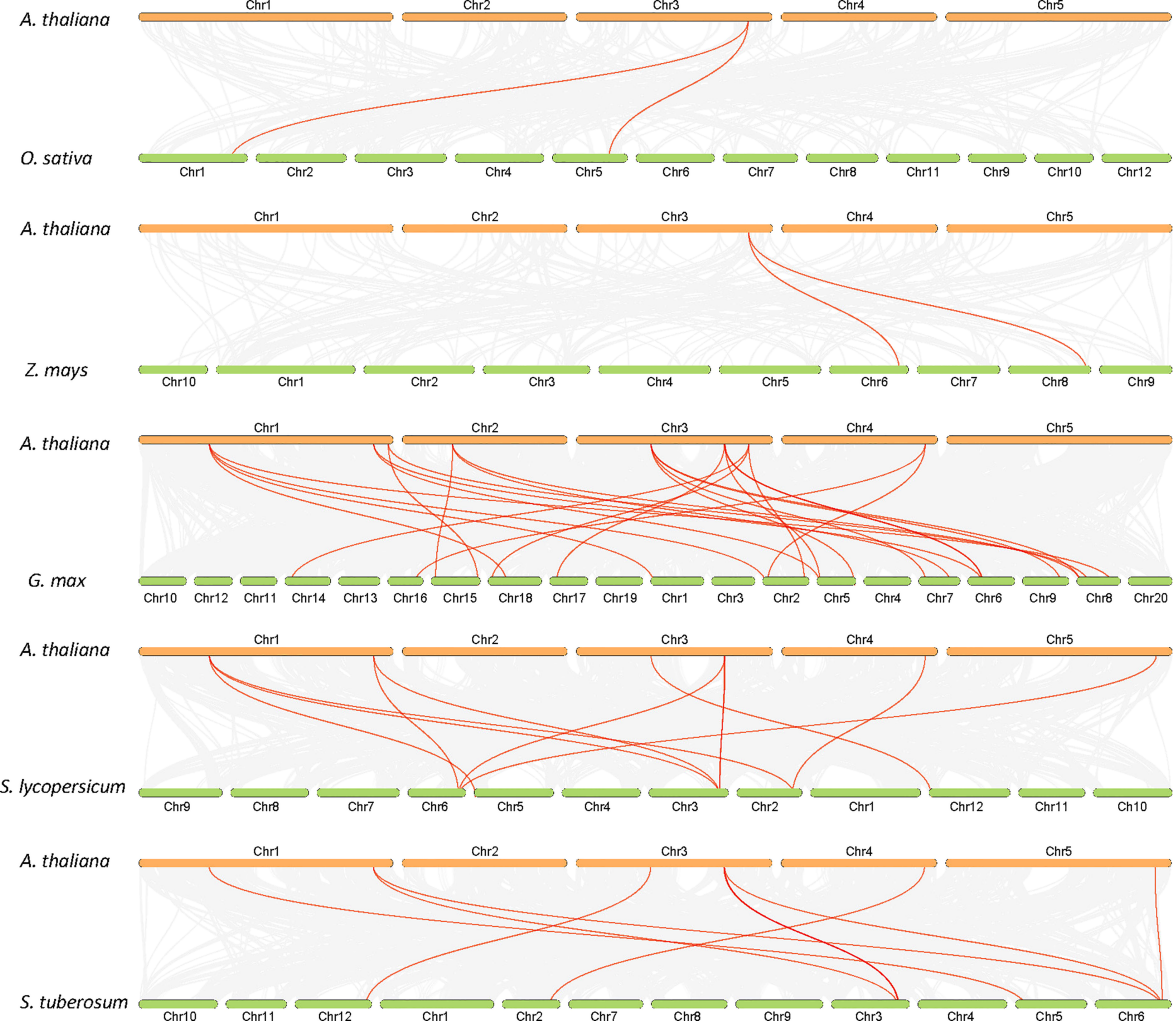
Synteny analysis of *ALDH* genes in Arabidopsis and other plants (*G. max*, *O. sativa*, *S. lycopersicum*, *Z. mays* and *S. tuberosum*). Gray lines in the background indicate collinearity blocks, whereas syntenic *ALDH* genes are shown as red lines.

The analysis of the chromosomal location of the *AtALDH* gene superfamily indicated that all the 16 *AtALDH* genes are located at 5 different chromosomes (**Figure 2**). Chromosomes 1 and 3 contained the greatest number of *AtALDH* genes, 5 *AtALDH* genes were located on chromosome 1 (*AtALDH2B7*; *AtALDH3H1; AtALDH7B4; AtALDH10A8; AtALDH5F1*) as well as chromosome 3 (*AtALDH22A1; AtALDH2C4; AtALDH2B4; AtALDH10A9; AtALDH18B2*), followed by Chromosome 2, which contained three *AtALDH* genes (*AtALDH6B2; AtALDH11A3; AtALDH18B1*). Chromosome 4 harbored two *AtALDH* genes (*AtALDH3I1; AtALDH3F1*) and chromosome 5 had only one *AtALDH* gene (*AtALDH12A1*). Interestingly, no gene duplication was found in the Arabidopsis *AtALDH* genes. Additionally, to further understand the evolutionary relationship of ALDH family members, collinearity analyses were conducted between *A. thaliana*, the model dicot and monocot crop plant species such as *G. max*, *O. sativa*, *S. lycopersicum*, *Z. mays* and *S. tubersoum*. A total of 10 AtALDHs (62.5%) were identified with collinearity relationships to ALDHs in other plant species, of which 9, 7, 7, 1, and 1 orthologous gene pairs were identified from *A. thaliana* - *G. max*, *A. thaliana* - *S. lycopersicum*, *A. thaliana* - *S. tuberosum*, *A. thaliana* - *Z. mays*, and *A. thaliana* - *O. sativa*, respectively (**Figure 3**).

### Exon-intron structure, conserved domain, motif distribution and phylogenetic analysis of AtALDHs

**Figure 4:**
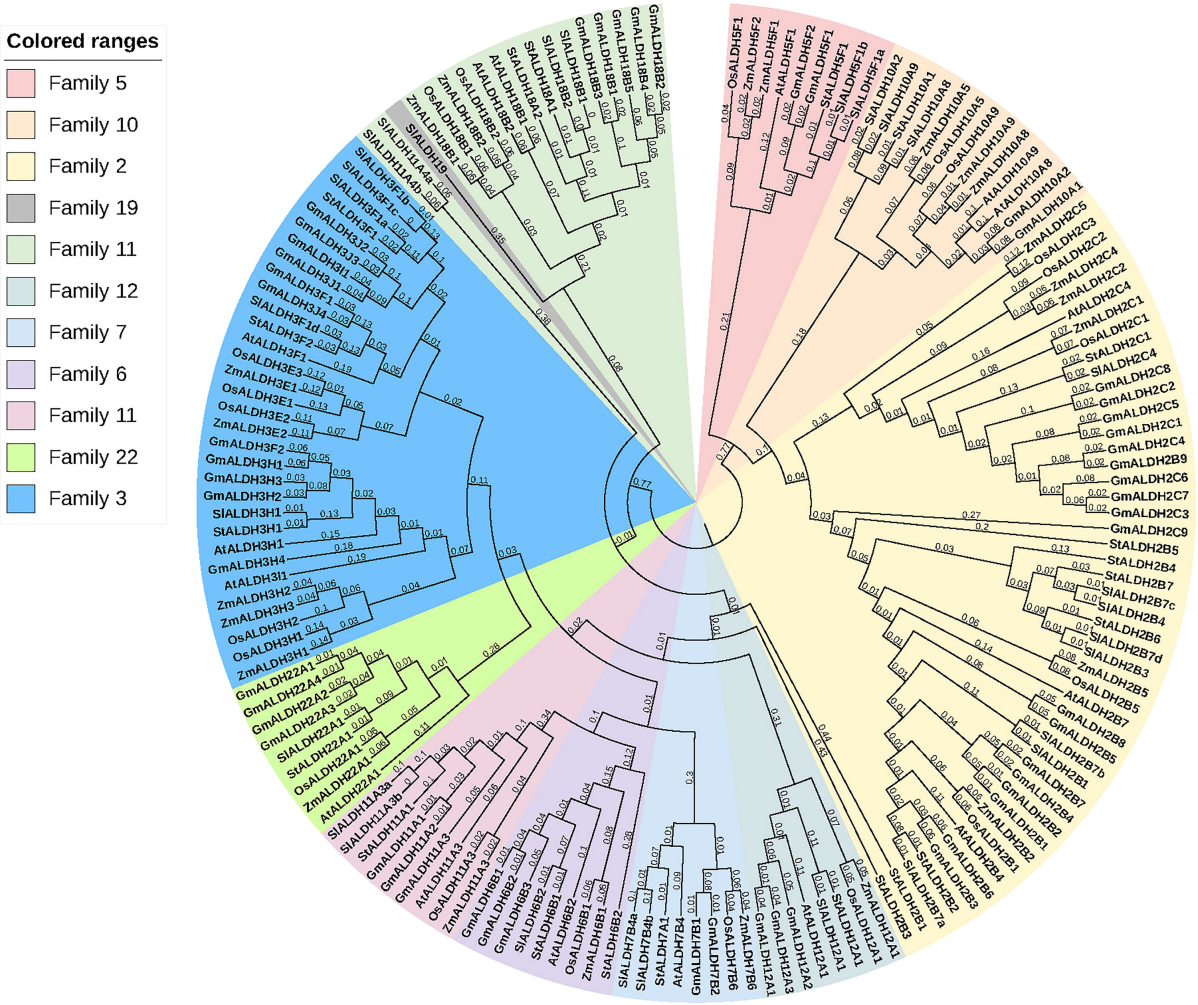
Phylogenetic analysis of ALDH superfamily proteins from various plant species. The subfamilies are highlighted in different colors. *At; A. thaliana, Os; O. sativa, St; S. tuberosum, Sl; S. lycopersicum, Zm; Z. mays, Gm; G. max*. The tree was constructed using the neighbor-joining method with 1000 bootstrap replications in MEGA 7.

**Figure 5:**
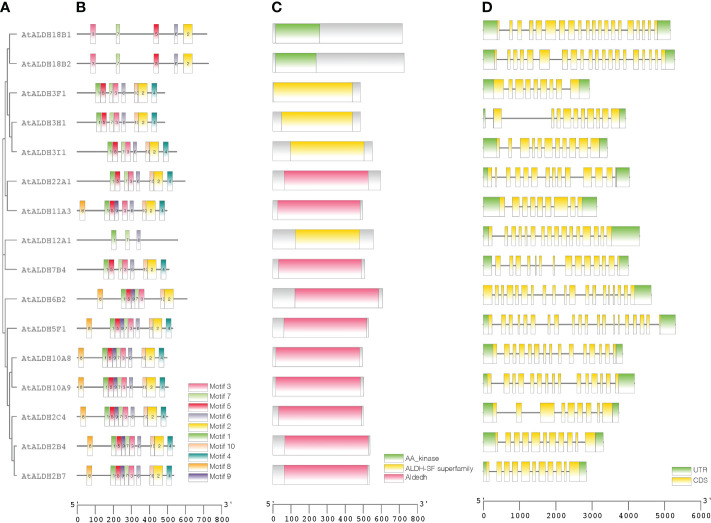
Protein sequence and gene structure analysis of ALDHs in Arabidopsis. Phylogenetic relationships, architecture of conserved protein motifs, and gene structure of *AtALDH* genes. **(A)** A phylogenetic tree was constructed based on AtALDH protein sequences, MEGA7's Maximum-likelihood method with 1000 bootstrap replicates was used to calculate genetic distances. **(B)** Motif composition of AtALDH proteins. The motifs, numbers 1-10, are displayed in different colored boxes. **(C)** Conserved domains of AtALDHs. **(D)** Exon-intron structure of *AtALDH* genes. Green boxes indicate untranslated 5’-UTR and 3’-UTR regions; yellow boxes indicate CDS regions; black lines indicate introns.

A phylogenetic tree was created using the protein sequences encoded by the *ALDH* genes to study the evolution and phylogeny of ALDH proteins. Protein sequences of ALDH from *A. thaliana* (16), *O. sativa* (20), *S. tuberosum* (22), *S. lycopersicum* (29), *Z. mays* (23) and *G. max* (53) were utilized to construct a phylogenetic tree (**Figure 4**). All the proteins were grouped into 11 subfamilies (ALDH2, 3, 5, 6, 7, 9, 10, 11, 12, 18, 19, 22). Surprisingly, most proteins from the same family were grouped together regardless of the reference species; however, ALDH19 was exclusively found in *S. lycopersicum*. ALDH 2 and 3 constituted the largest clusters among all the crop plants in this study, whereas ALDH 5, 12, and 22 had the fewest members. To further determine the evolutionary relationships of AtALDH proteins, using the MEGA-7 a phylogenetic tree was constructed applying the maximum likelihood method. The *AtALDH* genes from the particular subfamily clustered together in the phylogenetic tree (**Figure 5A**). The analysis of exon-intron structures of the *AtALDH* genes (**Figure 5D**) revealed that all the *AtALDH* genes have 5’-UTR and 3’-UTR, while the length of genomic DNA ranges from 2836 bp (*AtALDH2B7*) to 6494 bp (*AtALDH5F1*). The number of exons varies between individual members; among the 16 *AtALDH* genes we found that *AtALDH2C4* and *AtALDH3F1* had the lowest number of exons (nine each), while *AtALDH5F1*, *AtALDH18B1* and *AtALDH18B2* had the highest number of exons (20 each). The subfamilies may have similar numbers of exons, which may indicate functional similarity. The *ALDH10* subfamilies had equal numbers of exons.

To determine the presence of conserved protein domains of ALDHs, studies were performed using NCBI-CDD (https://www.ncbi.nlm.nih.gov/Structure/cdd/cdd.shtml). As shown in **Figure 5C**, 10 of the 16 AtALDH proteins (AtALDH2B4, AtALDH2B7, AtALDH2C4, AtALDH5F1, AtALDH6B2, AtALDH7B4, AtALDH10A8, AtALDH10A9, AtALDH11A3 and AtALDH22A1) had Aldedh (pfam00171), while 4 of the 16 AtALDH proteins (AtALDH3H1, AtALDH3I1, AtALDH3F1 and AtALDH12A1) had an ALDH-SF domain (cl11961). AtALDH18B1 and AtALDH18B2 had the AA_kinase (pfam00696) domain.

The MEME-suite was used to analyze the conserved motifs in the AtALDH proteins and as a result 10 motifs were determined. As can be seen in **Figure 5B**, motifs 8, 1, 5, 9, 7, 3, 6, 10 and 2 are present in AtALDH6B2, AtALDH5F1, AtALDH10A8, AtALDH10A9, AtALDH2B4, AtALDH2B7 and AtALDH2C4. The arrangement of conserved motifs was similar in most of the AtALDH proteins, except for AtALDH12A1 and the AtALDH18 subfamily. AtALDH12A1 contained 3 motifs (1, 7 and 6), while the AtALDH18 members comprised 5 motifs (3, 7, 5, 6, and 2).

### Analysis of *cis*-acting regulatory elements in the promoters of *AtALDH* genes

**Figure 6:**
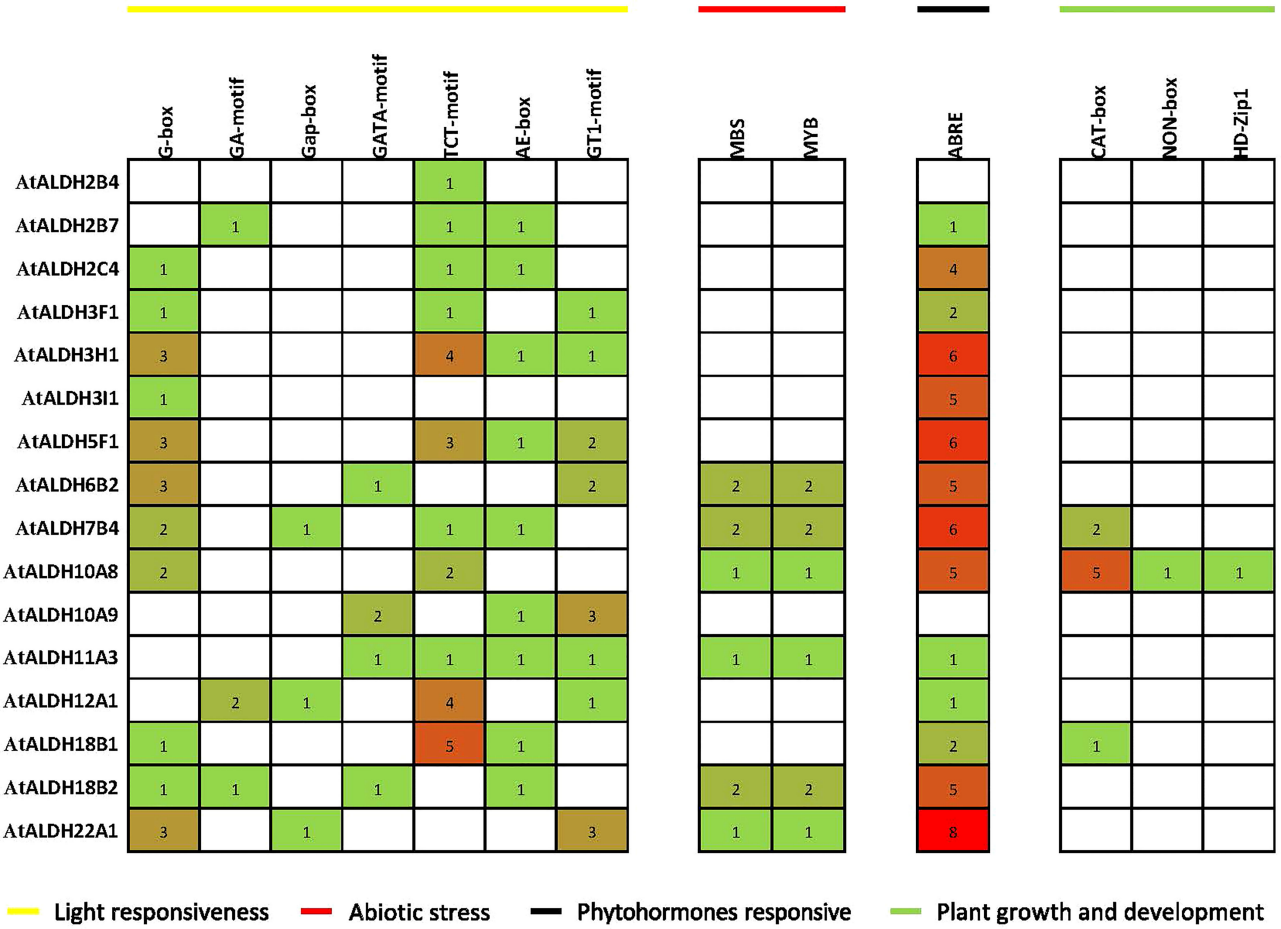
Distribution of *cis*-acting regulatory elements (CRE) in promoter sequences of *AtALDH* genes. The 1500 bp genomic DNA sequence upstream of the initiation codon (ATG) was extracted from the Eukaryotic Promoter Database (https://epd.epfl.ch/), potential CREs were analyzed by the PLACE database (http://www.dna.affrc.go.jp/PLACE/signalscan.html). The obtained data were visualized by PowerPoint (https://www.microsoft.com/en-us/microsoft-365/powerpoint). The yellow line indicates light responsiveness CREs; the red line indicates abiotic stress CREs; the black line indicates phytohormone responsive CREs; green line indicates plant growth and development CREs. The number in squares indicates the presence of CREs.

The PlantCARE database was used to detect the *cis*-acting regulatory elements (CREs) upstream 1500 bp of the Arabidopsis *ALDH* genes family. We identified a total number of 706 CREs in the promoter regions of the *AtALDH* genes (**Supplementary Material S3**). Based on functional categorization, the selected CREs (176) were further categorized as involved in plant development and growth, plant hormone response, light response, and stress responses (**Figure 6**).

The identified CREs were composed of a higher proportion of light-responsive and abiotic-responsive components, as well as a larger portion of TATA-box and CAAT-box basic elements (**Supplementary Material S3**). Light-responsive elements ranging from 1 to 5 included AE-boxes, G-boxes, TCT-motifs, G-box, Gap-box and GA-motifs. There were also substantial numbers of ABRE elements involved in the abscisic acid (ABA) responsiveness, ranging from 1 to 8. Plant growth and development elements ranged from 1 to 5 and they were engaged in meristem expression and palisade mesophyll cell proliferation. In the abiotic stress category MBS and MYB were the main elements implicated in drought-inducibility, ranging from 1 to 2.

### Functional annotation of *ALDH* genes in Arabidopsis

**Figure 7:**
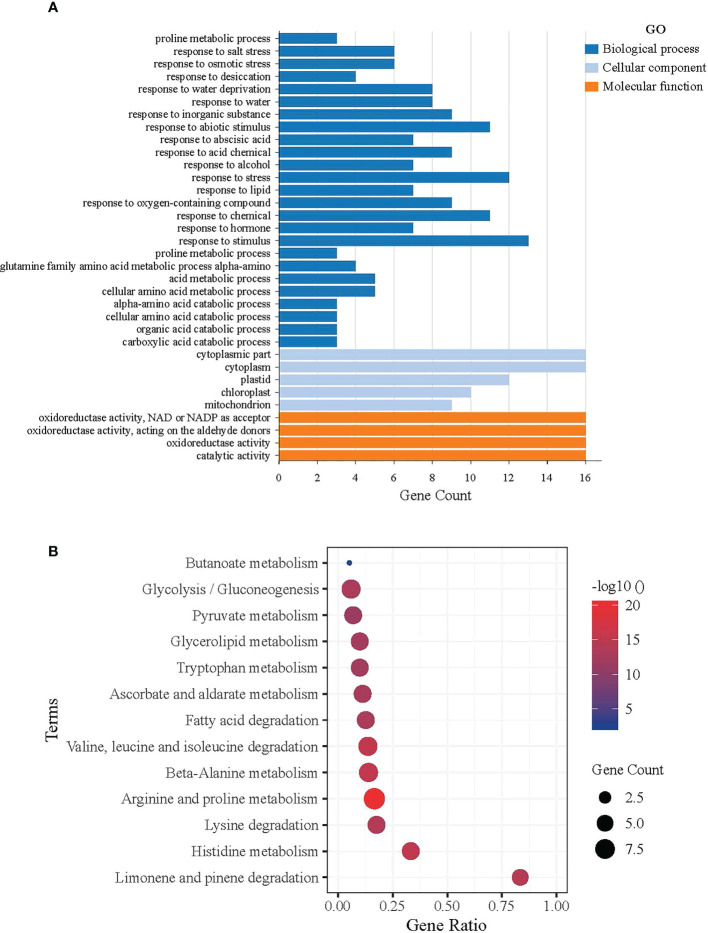
Functional analysis of *ALDH* in Arabidopsis. **(A)** GO functional annotation results, Molecular Function (MF), Cell Component (CC), and Biological Process (BP). The horizontal axis shows the gene count of *AtALDH* genes, while the vertical axis represents the biological process, molecular function, and cellular component, respectively. **(B)** Significantly Enriched Kyoto Encyclopedia of Genes and Genomes (KEGG) pathways. The horizontal axis shows the gene ratio of *AtALDH* genes, while the vertical axis represents the significant biological pathway. The Database for Annotation, Visualization and Integrated Discovery (https://david.ncifcrf.gov/home.jsp) was used for both GO annotation and KEGG pathway analysis of ALDH in Arabidopsis, with default parameters.

The David Functional Annotation tool was used to annotate the *AtALDH* genes with functional databases for the GO (gene ontology) and KEGG pathways. **Figure 7A** shows the results of the *AtALDH* genes annotated in the GO database. The *AtALDH* genes were divided into three categories: molecular function (MF), cell component (CC), and biological process (BP). The largest proportion of the molecular function was aldehyde dehydrogenase activity (NAD) and oxidoreductase activity. In the biological process categories the most significant proportion was distributed in the cellular aldehyde metabolic process. In the category of cell components, the highest proportion of *ALDHs* was distributed in the cytosol.

KOBAS was used to test the statistical enrichment of the KEGG pathways. **Figure 7B** shows the results of the *AtALDH* genes in 13 pathways in the KEGG database. The *AtALDH* genes enriched pathway in limonene and pinene degradation had the largest numbers of the *AtALDH* genes. Meanwhile, the other *AtALDH* genes enriched pathways were histidine metabolism, lysine degradation, as well as arginine and proline metabolism.

### Expression analysis of *AtALDH* genes during development stages

**Figure 8:**
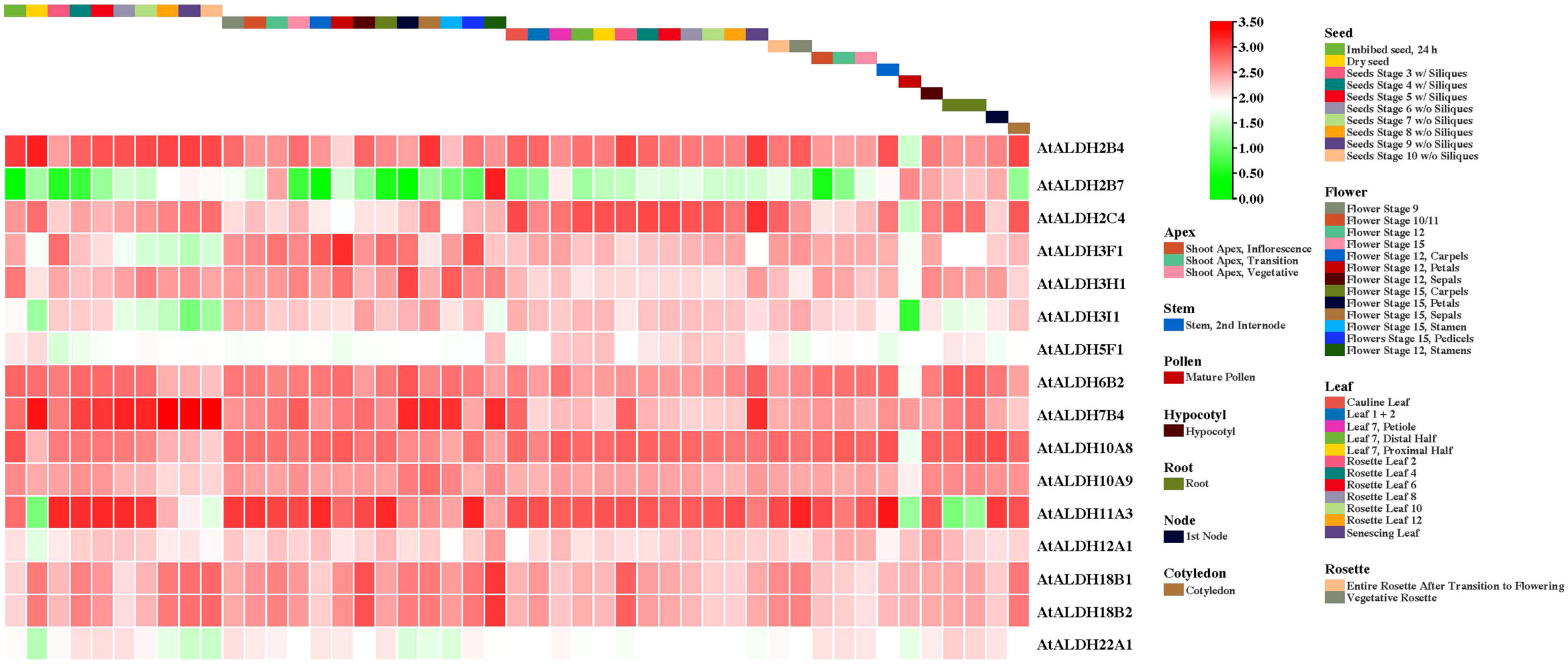
The *AtALDH* gene expression profile during plant growth and development stages of Arabidopsis. The *AtALDH* gene expression profile was extracted from the Arabidopsis eFP browser (http://bar.utoronto.ca/efp//cgi-bin/efpWeb.cgi ). Regarding growth and development stages of Arabidopsis, the expression profiles of 16 *AtALDH* genes were investigated during different stages of development (seed, flower, leaf, rosette, apex, stem, pollen, hypocotyl, root, node, and cotyledon). The scale of figure indicates the fold change of *AtALDH* genes.

All the *AtALDH* genes were investigated in various organs, according to the developmental map of the *AtALDH* genes during different stages of development: seed, flower, leaf, rosette, apex, stem, pollen, hypocotyl, root, node, and cotyledon, respectively (**Figure 8**). As shown, almost every member of the *AtALDH* family is engaged in every stage of development. However, some *AtALDH* genes are tissue-specific, such as *ALDH2B7*, which is abundantly expressed in the flower stage of 12 stamens. Similarly, *AtALDH7B4* is primarily involved throughout the seed stages.

### Expression profile of *AtALDH* genes under hypoxia stress

**Figure 9:**
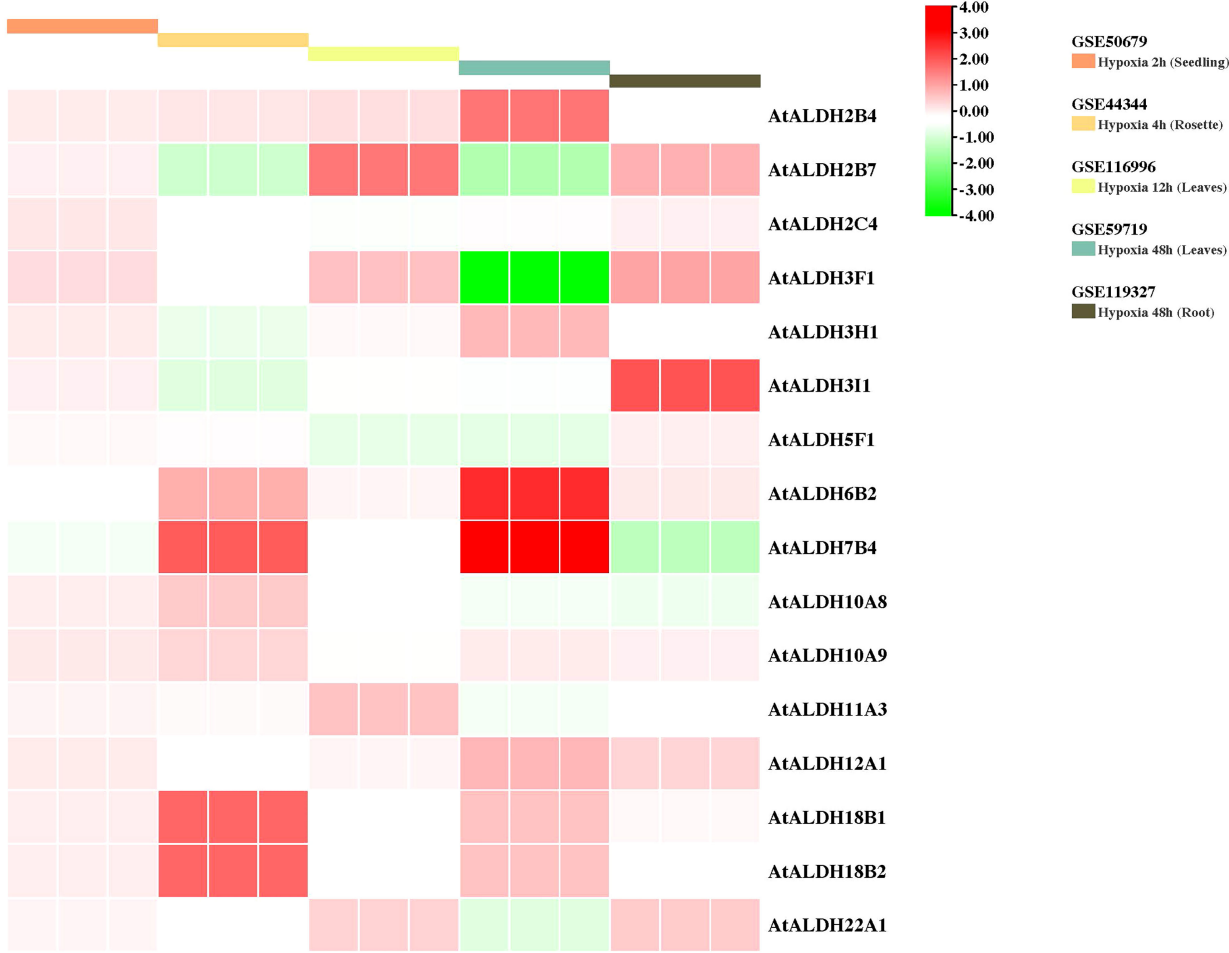
Gene expression profiles of *AtALDH* genes in Arabidopsis under hypoxia stress. The microarray datasets used in this analysis are presented in coloured rectangular boxes. Five microarrays related to hypoxia stress in Arabidopsis were extracted from the GEO database (https://www.ncbi.nlm.nih.gov/geo/ ), GSE50679 (Arabidopsis seedling with 2h hypoxia treatment); GSE44344 (Arabidopsis rosette with 4h hypoxia treatment); GSE116996 (Arabidopsis leaves with 12h hypoxia treatment); GSE59719 (Arabidopsis leaves with 48h hypoxia treatment); and GSE119327 (Arabidopsis root with 48h hypoxia treatment). The scale of the graph represents the log fold change value for the *AtALDH* genes.

To gain further insight into the *AtALDH* gene responses to hypoxia stress, five microarrays available in the databases were used to identify the pattern of the *AtALDH* genes under multiple stages of hypoxia stress: 2h (seedling), 4h (rosette), 12h (leaves), and 48h (leaves and roots) (**Figure 9**). *AtALDH2B4* was shown to be upregulated as hypoxia severity increased, while *AtALDH2C4*, *AtALDH6B2*, *AtALDH7B4*, *AtALDH10A9*, *AtALDH12A1*, *AtALDH18B1* and *AtALDH18B2* showed a similar trend. After 48h hypoxia treatment among all the 16 *AtALDH* genes *AtALDH6B2* and *AtALDH7B4* demonstrated the most significant upregulation, while *AtALDH3F1* was shown to be the most downregulated. Importantly, expression of the selected *AtALDH* genes at 48h of stress in leaves and roots revealed opposing tendencies, e.g. *AtALDH3F1* and *AtALDH22A1* were upregulated in leaves, whereas they were downregulated in roots. This may refer to the abundance of the *ALDH* genes in different tissue types. Obtained results indicated that *AtALDH3F1*, *AtALDH6B2* and *AtALDH7B4* were the most hypoxia-responsive.

### qRT-PCR analysis of *AtALDH* genes under hypoxia in *A. thaliana* leaves

**Figure 10:**
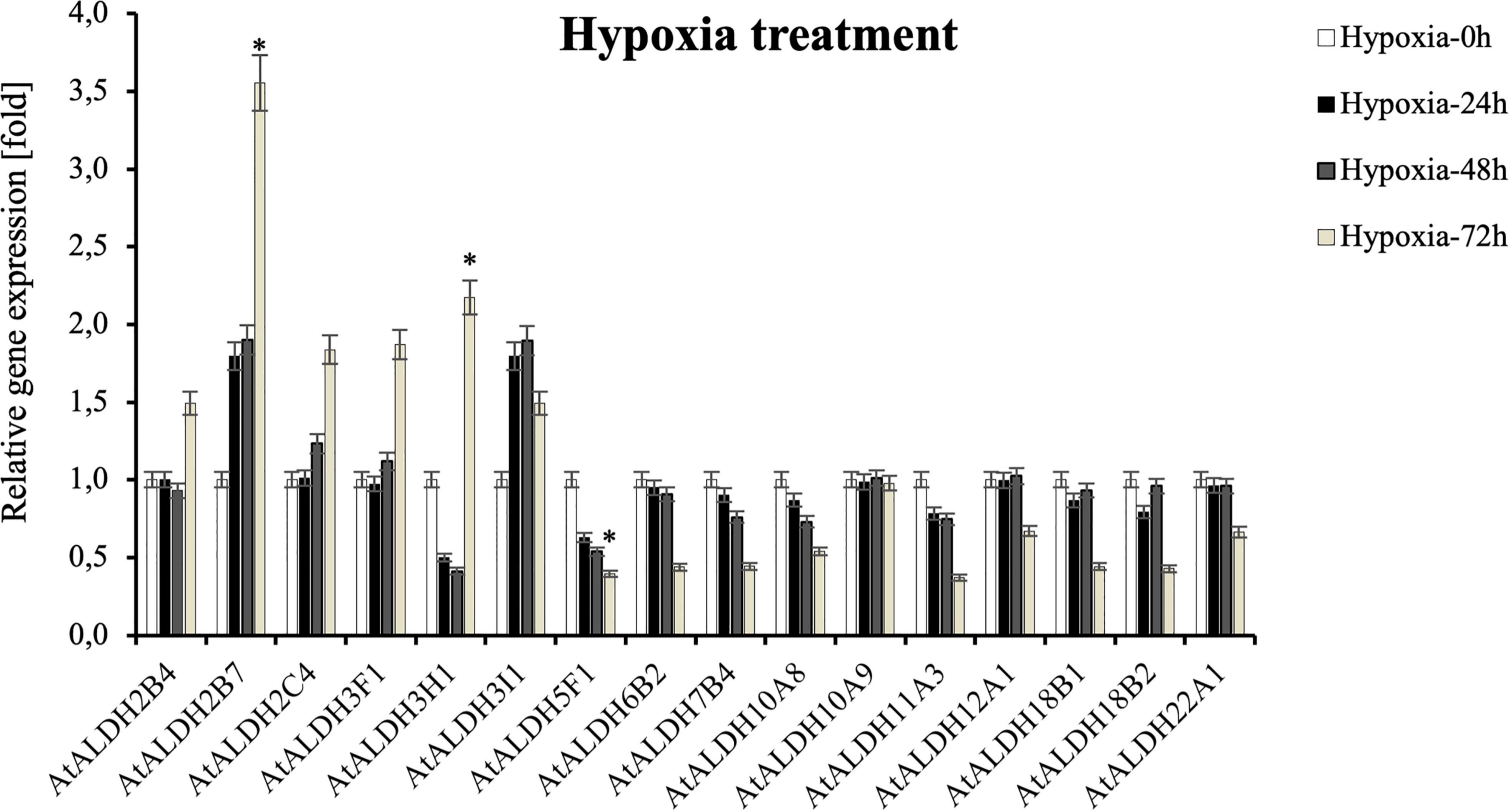
Relative gene expression of *AtALDHs* under hypoxia. Analyses were performed in Arabidopsis leaves of plants exposed to stress at selected time points: 0h (control), 24h, 48h, and 72h. All values represent the means of data ± SD of at least three independent experiments (*n*=9). Asterisks (*) indicate values that differ significantly after hypoxia treatment as compared to 0h (control) at α<0.05. The analysis of variance was conducted and the least significant differences (LSDs) between means were determined using Tukey’s test at the level of significance α=0.05.

Since flooding creates a natural hypoxia condition, Arabidopsis was partially submerged in water to evaluate the impact of the stress on *AtALDHs* in leaves. As shown in **Figure 10**, hypoxia at 72h caused the most significant modifications in *ALDH* expression. The most impressive transcript accumulation was observed in the case of *AtALDH2B7*, which increased almost 3.5-fold at 72h of hypoxia in comparison to the control. In general, stress conditions provoked upregulation of *AtALDH2B4, AtALDH2C4, AtALDH3F1, AtALDH3H1* and *AtALDH3I1.* Diminished transcript accumulation was observed for *AtALDH5F1*. A similar tendency was observed in the case of *AtALDH6B2, AtALDH7B4, AtALDH10A8, AtALDH11A3, AtALDH12A1, AtALDH18B1, AtALDH18B2* and *AtALDH22A1*. Interestingly, expression of *AtALDH10A9* did not alter much during hypoxia.

### qRT-PCR analysis of *AtALDH* genes under recovery phase in *A. thaliana* leaves

**Figure 11:**
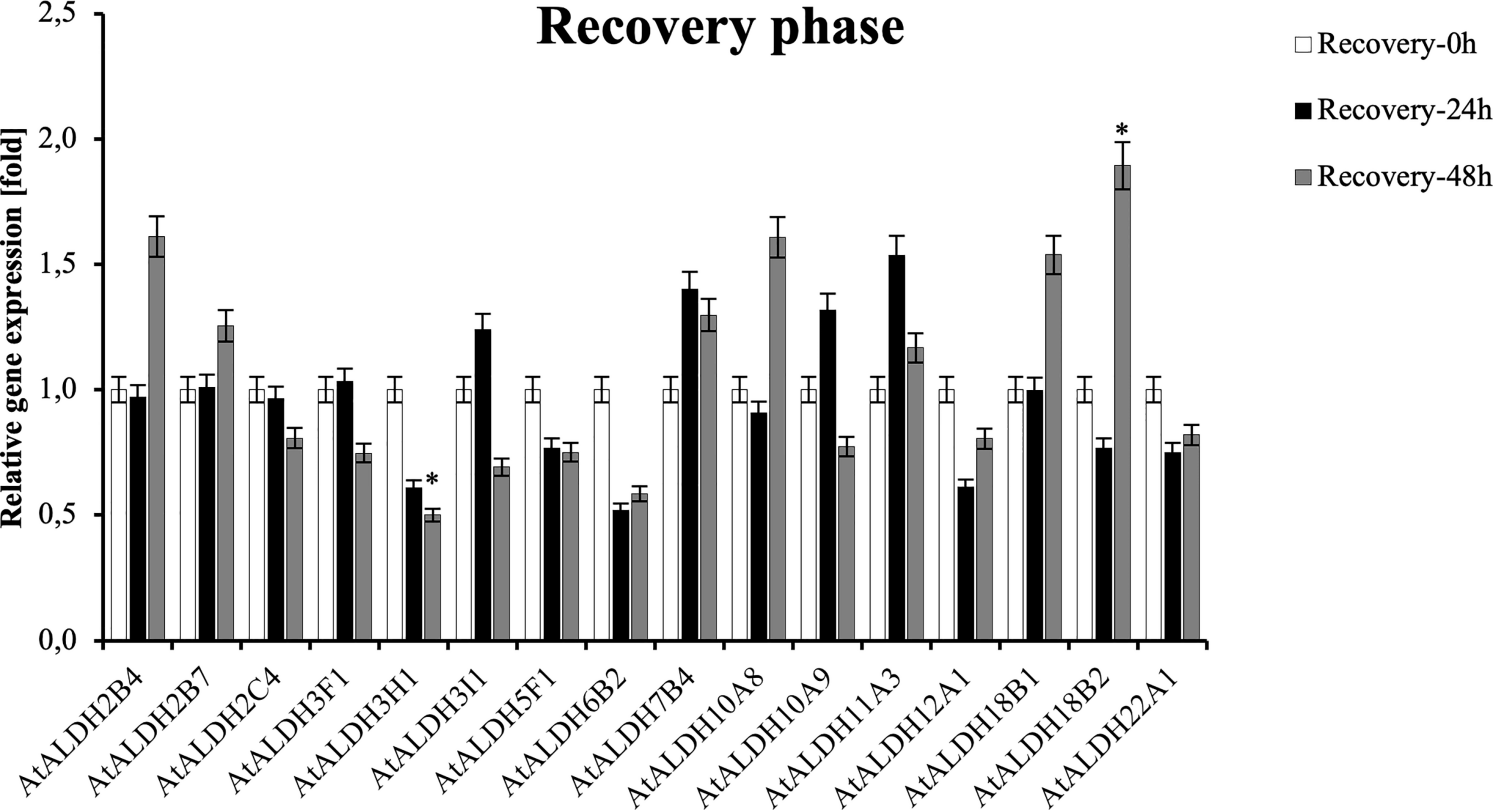
Relative gene expression of *AtALDHs* under recovery phase. Analyses were performed in Arabidopsis leaves of plants recovered from 24 h hypoxia stress at selected time points: 0h (control), 24h, 48h. All values represent means of data ± SD of at least three independent experiments (*n*=9). Asterisks (*) indicate values that differ significantly during recovery phase as compared to the control at α<0.05, respectively. The analysis of variance was conducted and the least significant differences (LSDs) between means were determined using Tukey’s test at the level of significance α=0.05.

To investigate the expression pattern of the *ALDH* genes during post-hypoxia reoxygenation (recovery phase), Arabidopsis plants were partially submerged in water for 24h, followed by recovery for 24 h and 48 h. As shown in **Figure 11**, *ALDH18B2* was the most upregulated gene after 48 h recovery, nearly 2-fold when compared with the control. Generally, the recovery phase led to the upregulation of *ALDH2B4, ALDH2B7, ALDH3I1, ALDH7B4, ALDH10A8, ALDH10A9, ALDH11A3, ALDH18B1, ALDH18B2* and *ALDH22A1*. The most decreased transcript accumulation was observed for *ALDH3H1* after 48h recovery, while a similar tendency was also observed in *ALDH2C4, ALDH3F1, ALDH3I1, ALDH5F1, ALDH6B2, ALDH12A1* and *ALDH22A1*. Interestingly, the expression of the *ALDH3I1* and *ALDH10A9* genes was upregulated at 24 h recovery and downregulated as the recovery phase progressed to 48 h.

### Homology modelling of AtALDH proteins encoded by the hypoxia responsive genes

**Figure 12:**
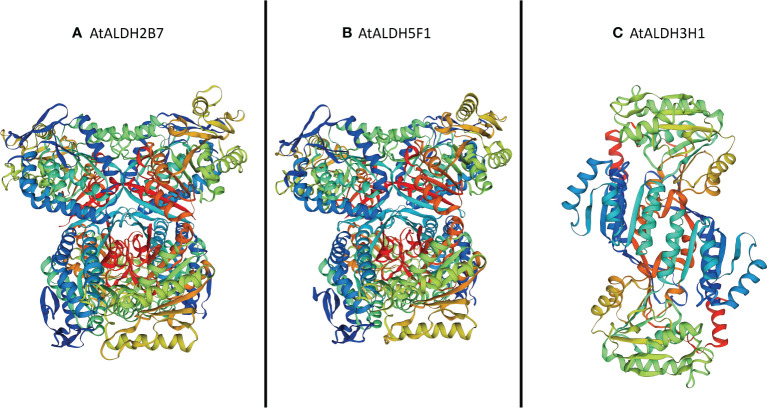
Three-dimensional structure analysis of the proteins encoded by three hypoxia responsive *AtALDH* genes. **(A)** AtALDH2B7; **(B)** AtALDH5F1; and **(C)** AtALDH3H1. All the structures were visualized by rainbow color from N to C terminus. Coils and smooths represent alpha helices and beta sheets, respectively.

The SOPMA (self-optimized prediction method with alignment) was employed to predict the ratio of alpha helices, extended strands, beta turns, and random coil in all the AtALDH proteins (**Table S8**). Among all secondary structure prediction of AtALDH proteins, the alpha helix predominates, ranging from 33.44% to 50.96%, followed by the random coil (24.66%-41.01%), extend strand (12.58%-19.60%) and beta turn (4.53%-8.90%). Meanwhile, protein glycosylation of AtALDHs was also determined in this study, as shown in **Table S6**. The results indicated that 13 out of 16 AtALDH proteins have N-glycosylation sites, with AtALDH18B1 having the largest number of N-glycosylation sites, i.e. 7. The derived homology models were verified using the Procheck Ramachandran plot analysis. The majority of the residues of AtALDH2B7, AtALDH3H1, and AtALDH5F1 were located in the preferred region of 93.0%, 91.8%, and 93.0%, respectively (**Table S9**). The homology model revealed that the overall structure of AtALDH2B7 and AtALDH5F1 was very similar in terms of common strands and helices in the Rossmann folding type (**Figure 12**).

## Discussion

Aldehyde dehydrogenases are a group of enzymes involved in NAD^+^/NADP^+^ dependent conversion of various aldehydes to their non-toxic carboxylic acids (Brocker et al., 2013). Plant *ALDH* genes play an important role not only in seed germination and developmental stages, but also in oxidative stress responses under plant dehydration or high salinity (Guo et al., 2020; Islam et al., 2021; Islam and Ghosh, 2022). Flooding caused by excessive or persistent rainfall in a region with poorly drained soil is one of the most serious environmental stresses. According to estimates from the Food and Agriculture Organization of the United Nations (FAO), between 2008 and 2018 floods caused losses of around US$ 21 billion to agriculture in the developing countries (https://www.fao.org/resources/digital-reports/disasters-in-agriculture/en/). Flooding caused by waterlogging or submersion frequently results in hypoxia in plants. In general, hypoxia, the oxygen (O_2_) depletion due to water’s lower oxygen availability than that of air, disrupts metabolic processes leading in consequence to plant growth inhibition and cell death. ALDH has been recognized to be an aldehyde scavenger that eliminates toxic aldehydes induced by oxidative stress, but its involvement in unique metabolic conversions induced by hypoxia is still poorly understood. A comprehensive investigation of the *ALDH* superfamily genes in a model plant Arabidopsis was therefore undertaken in order to clarify the role of *ALDHs* in response to hypoxia stress.

In Arabidopsis a total of 16 *ALDH* genes have been identified, which were grouped into 10 subfamilies (Families 2, 3, 5, 6, 7, 10, 11, 12, 18 and 22). In order to evaluate the sequence resemblance and evolutionary relationships of the *ALDH* genes in Arabidopsis, *ALDHs* from five different crop plants (*Solanum lycopersicum, Solanum tuberosum, Zea mays, Oryza sativa, Glycine max*) were used. Phylogenetically, the ALDHs in the selected crop plants were grouped into 11 subfamilies (Families 2, 3, 5, 6, 7, 10, 11, 12, 18, 19 and 22). Interestingly, ALDH19 was found only in *S. lycopersicum*. Importantly, *A. thaliana* ALDH members were clustered together with five other crop plants into the same family. Phylogenetic analysis showed that the *ALDH* genes are highly conserved across the monocotyledonous and dicotyledonous plants. It implies that the plant *ALDH* genes evolved prior to the divergence of monocots and dicots. Previously, tandem duplication events had been recorded in *O. sativa* (*OsALDH2C1* and *OsALDH2C2*; *OsALDH3E1* and *OsALDH3E3*) (Gao and Han, 2009) and *S. tuberosum* (*StALDH2B4*, *StALDH2B4*, and *StALDH2B6*; *StALDH18B1* and *StALDH18B2*) (Islam et al., 2021). However, no gene duplication event was found in *A. thaliana*, which might be due to the relatively small genome size (approximately 135MB) of *A. thaliana* as compared with other plants (The Arabidopsis Genome Initiative, 2000). Meanwhile, synteny analysis of *ALDH* genes among Arabidopsis and other plants revealed that the correlation between *AtALDH* genes and *GmALDH* genes is similar, which is significant when exploring the relationship between species and forecasting gene functions. Furthermore, the Codon-based d_N_/d_S_ ratios (**Table S5**.) of all the *AtALDH* genes were less than one, which supports the evolution of *AtALDH* genes by purifying selection pressure during the evolutionary process. Moreover, phylogenetic analysis indicated that the *AtALDH* families 2, 5, 6 and 10 are the most closely related, while additionally *AtALDH* family 18 is the most phylogenetically distant. Due to the fact that AtALDH18B1 and AtALDH18B2 contained a completely separate conserved domain (AA kinase), it is possible that these two AtALDHs differ significantly from the other AtALDHs. Furthermore, in this study we also determined glutamic acid and cysteine acid activity sites in AtALDHs. A cysteine acid activity site is present in 10 out of 16 AtALDHs. A glutamic acid and a cysteine residue have been implicated in the catalytic activity of mammalian aldehyde dehydrogenases (Farres et al., 1995). It is worth noticing that the nitric oxide signaling through redox-based modification of protein cysteine residues known as S-nitrosylation can affect a broad range of proteins (Nakamura and Lipton, 2011; Wang et al., 2022). Thus, the presence of the cysteine acid active site in ALDH may constitute a target of nitric oxide dependent S-nitrosylation, and function as a metabolic sensor of nitric oxide signaling.

In this study we determined the *cis*-acting regulatory elements (CREs) in the promoter regions of the *AtALDH* genes. The identified CREs were found to be involved in growth and development, light, phytohormone and stress responses. We also analyzed the expression of the *AtALDH* genes in different organs using the publicly available database. The obtained results indicated that almost all the *AtALDH* genes were engaged in every stage of development (**Figure 8**).

Plant phytohormones play pivotal roles in stress adaptation of plants (Waadt et al., 2022). In previous studies phytohormones have been reported to regulate the expression of the *ALDH* genes (Wu et al., 2007). In Arabidopsis abscisic acid can regulate *ALDH3* gene expression (Brocker et al., 2013). In turn, in *Z. mays* the phytohormone elevated *ALDH22A1* gene expression (Huang et al., 2008). It was also documented that *ALDH6* was upregulated in *O. sativa* treated with auxin and gibberellin (Oguchi et al., 2004; Marchitti et al., 2008). A recent study indicated that hypoxia stress can led to the accumulation of ethylene, which may further regulate ABA catabolism to impact plant hypoxia tolerance (Wang et al., 2021). In our study we determined that the ABA-responsive element (ABRE) was present in 14 out of 16 *AtALDHs* (*ALDH2B7, ALDH2C4, ALDH3F1, ALDH3H1, ALDH3I1, ALDH5F1, ALDH6B2, ALDH7B4, ALDH10A8, ALDH11A3, ALDH12A1, ALDH18B1, ALDH18B2, ALDH22A1*), which can be induced by ABA in Arabidopsis. These results indicate that *ALDHs* might play an important role in response to hypoxia stress through the regulation of ABA.

Another crucial aspect that was taken into account when searching for the *AtALDHs* involvement in adaptation to hypoxia stress is the hypoxia condition. In this experimental context Tsuji et al. (2003) showed that the hypoxia condition can regulate the expression of the *ALDH2A* in rice. Based on the analysis of the expression profile of the *AtALDH* genes from the microarray datasets our analysis confirmed that the hypoxia condition could regulate the expression of the *AtALDH* genes. Moreover, data revealed that the *AtALDH* genes might be differently expressed in various organs with the same stressful scenario. For example, *ALDH7B4* was up-regulated in leaves, but down-regulated in roots after 48 h hypoxia stress. This finding was further supported by the GO and KEGG database annotations. Eight out of 16 *AtALDH* genes (*ALDH11A3, ALDH10A9, ALDH3I1, ALDH3H1, ALDH18B1, ALDH7B4, ALDH10A8, ALDH2B7*) have been found to be responsive to water, which also includes response to flooding and submergence in water. Plants encounter multiple challenges during hypoxia at flooding conditions. Under these circumstances the ability of plant cells to absorb CO_2_ for photosynthesis and O_2_ for respiration is severely hampered by the substantial drop in gas diffusion. Additionally, plants may experience cellular energy and glucose shortages due to the lower light availability under water (MOMMER and VISSER, 2005). Hence, plant cells switch from aerobic respiration to anaerobic fermentation, accumulating toxic metabolites such as lactic acid, acetaldehyde and ethanol, which cause further cell damage. During O_2_ deprivation pyruvate decarboxylase (PDC) converts pyruvate to acetaldehyde, which is metabolized by alcohol dehydrogenase (ADH) to ethanol, while regeneration of NAD+ sustains glycolysis. Ethanol production is benign owing to its rapid diffusion out of cells, whereas the intermediate acetaldehyde is toxic. Aldehyde dehydrogenase catalyzes the conversion of acetaldehyde to acetate, with the concomitant reduction of NAD+ to NADH. The mitochondrial ALDH is significantly induced by anoxia in coleoptiles of rice (Nakazono et al., 2000; Lasanthi-Kudahettige et al., 2007), in contrast to the Arabidopsis seedlings (Kürsteiner et al., 2003). ALDH activity correlates with the anaerobic germination capability of *Echinochloa crus-galli* under strict anoxia (Fukao et al., 2003). Under O_2_-limiting conditions ALDH also consumes NAD+ and may thereby limit glycolysis, whereas upon reoxygenation acetaldehyde is converted to acetate by mitochondrial ALDH that enters the tricarboxylic acid (TCA) cycle. Increasing evidence suggests that an oxidative burst resulting from the ROS production started immediately upon exposure of anaerobic plant tissues to normoxia leads to severe peroxidation of cellular components. The enzymatic scavenging of aldehydes derived from stress-related lipid peroxidation involves ALDHs. ALDHs are also engaged in a variety of other activities, such as (i) controlling secondary metabolism, particularly amino acid and retinoic acid metabolism (Zhang et al., 2012); (ii) generating osmoprotectants such as glycine betaine to protect against osmotic stress (Wani et al., 2013; Xiao and Loscalzo, 2020); and (iii) contributing to the maintenance of redox equilibrium. ALDHs also play an important role in cellular homeostasis by maintaining cellular redox equilibrium; for example, ALDHs may scavenge hydroxyl radicals *via* the thiol groups of their cysteine and methionine residues (Estey et al., 2007). Additionally, ALDH isozymes may contribute to the cellular antioxidant capacity by generating NAD(P)H, which is critical for the regeneration of GSH and as a direct antioxidant (Singh et al., 2013).

Although the root is the first organ that senses hypoxia caused by flooding, a set of physiological and biochemical changes are also induced in leaves. Leaves are usually fully aerobic tissues and also produce oxygen through photosynthesis. Leaves are the most variable organs in long-term adaptation to the environment. Therefore, in this study to verify *AtALDH* gene expression patterns under hypoxia conditions Arabidopsis plants were submerged in the water tank to assess the hypoxia effect on leaf metabolism adjustment. Obtained qRT-PCR results suggest that hypoxia for 72h provokes the most significant modifications in *AtALDH* gene expression (**Figure 10**). The expression of *AtALDH2B7* and *AtALDH3H1* was significantly upregulated during hypoxia and both of them were localized in the cytoplasm (Hou and Bartels, 2015). Additionally, the *AtALDH5F1* gene was downregulated during hypoxia; interestingly, ALDH5F1 is mitochondrial specific (Hou and Bartels, 2015). Thus, our investigation may indicate that different *AtALDH* genes have different expression patterns in an organelle-specific manner. Previous research showed that in Arabidopsis the *ALDH2B7* gene was expressed not only in anoxia, but also in response to drought, as evidenced by the downregulation of glycolysis and the stimulation of acetate production (Kim et al., 2017). *ALDH3H1* is constitutively expressed at a low level in leaves, but is activated in response to osmotic stress and after ABA treatment in roots (Kirch et al., 2004). Notably, it results from our *cis*-elements analysis that *AtALDH3H1* also has 6 ABRE elements in the promoter region. Plant hormones such as ethylene and ABA play crucial roles in the genetically controlled survival of plants to hypoxia under waterlogging or submergence (Phukan et al., 2016). Ethylene has been extensively characterized as a critical hormone in hypoxia-triggered responses (Geisler-Lee et al., 2010; Hartman et al., 2019; Waadt et al., 2022). Therefore, ALDH3H1 might play an essential role in regulating hypoxia responses through the regulatory network involving an interplay between ROS, ABA, and ethylene. Extended hypoxic conditions can result in decreased ATP synthesis and cytoplasmic acidosis, both of which can damage plant cells. However, plant cells may also be damaged during reoxygenation after hypoxia as a result of the formation of ROS and acetaldehyde (Tsuji et al., 2003). Hereby, the expression pattern of *ALDHs* under the recovery phase was also investigated in our study. During the reoxygenation, *ALDH18B1*, *ALDH18B2*, *ALDH2B4* and *ALDH10A8* were found to be upregulated; meanwhile, ALDH18B1, ALDH18B2 and ALDH2B4 have been reported to be localized in the mitochondria (Hou and Bartels, 2015). It should be noted that under O_2_-limiting conditions ALDH also consumes NAD+ and may limit glycolysis, whereas upon reoxygenation acetaldehyde is converted to acetate by mitochondrial ALDH and enters the tricarboxylic acid (TCA) cycle. ALDH10A8 is a leucoplastidial protein, which was found upregulated in response to abscisic acid, salinity, cold and oxidative stress in Arabidopsis (Missihoun et al., 2011). Compared with post-hypoxia treatment, *AtALDH3H1* was downregulated, while the expression of *AtALDH2B7* was not changed during reoxygenation. This implies that they are the most hypoxia-responsive *ALDH* genes. Additionally, the *AtALDH5F1* gene was downregulated during hypoxia and reoxygenation conditions. ALDH5F1 is mitochondrial specific. In a previous study the expression of the *ALDH5F1* gene was found to be regulated by waterlogging stress in sugarcane (Gomathi et al., 2015). Moreover, *ALDH5* Arabidopsis mutants presented ROS over-accumulation and cell death in response to light and heat stress (Bouche et al., 2003). ALDH5 was found to participate in the γ-aminobutyrate (GABA) ‘shunt’ pathway in bacteria, plants, and animals. It worth noting that in plants the non-protein amino acid GABA is associated with pollen-pistil interactions, herbivore deterrence, oxidative stress, and hypoxia, as a its high concentration enhances plant defence or tolerance responses (Brocker et al., 2013).

Cellular function is accomplished by 3D well-folded protein structures, protein-protein and protein-ligand interactions (Jimenez-Lopez et al., 2010). In this study we identified three hypoxia responsive *AtALDH* genes, and the encoded proteins were homology modelled using swiss-modelling. The 3D structures of all the proteins showed the number of residues > 90% in the most favoured region as per the Ramachandran plot analysis, which suggests the high accuracy of the structure prediction. Protein glycosylation is one of the crucial components of protein structure, which regulate a range of biological activities including protein folding, signalling, stability, conformation, and cell-cell interactions (Corfield, 2017). In this study, N-glycosylation sites were recognized for all the AtALDH proteins, while 13 out of 16 AtALDHs contained N-glycosylation sites. In eukaryotic cells protein N-glycosylation is one of the most important post-translational modifications. By altering protein functions, it mediates diverse biological processes, including intercellular communication (Chen et al., 2020). The numerous functions of N-glycosylation in regulating plant stress tolerance and development were previously reported (Kaulfürst-Soboll et al., 2021). The glycosylation in multiple ways is involved in maintaining the redox homeostasis during the plant’s response to oxidative stress, including i) modification of a molecule’s antioxidant property, as in the case of flavonoids, ii) promoting the biosynthesis of its corresponding aglycone by changing its subcellular location, and iii) influencing the phytohormone translocation, signalling capacity, and downstream gene expression regulation (Behr et al., 2020). Therefore, in our study, it can be assumed that after hypoxia stress, during recovery, the ALDHs might be associated with redox-related patterns and signalling pathways for glycol-redox interplay in response to oxidative stress.

## Conclusion

We characterized the *ALDH* genes in Arabidopsis at the whole-genome scale to provide insight into their genomic and structural organization, regulatory framework, physicochemical properties, phylogenetic and evolutionary relationships, as well as expression profiles during developmental stages and under abiotic stresses. In addition, functional validation of the *ALDH* genes in Arabidopsis leaves carried out by qRT-PCR analysis help indicate that the candidate genes are responsive to hypoxia and post-hypoxia reoxygenation. Thus, a future detailed characterization of the selected genes will provide comprehensive understanding of the ALDH role in mechanisms of hypoxia tolerance and post-hypoxia recovery. Notably, different patterns of *ALDHs* expression observed during stress and recovery phases indicated that plant ALDH is a crucial element of the oxygen-dependent metabolic switch in cells. The results broaden our knowledge on plant ALDHs and provide valuable information for future genetic improvement programs in crop plants potentially challenged by hypoxia stress. It worth noting that hypoxia in nature can also be caused both by pathogen infestation or occur sequentially with flooding events (Tang and Liu, 2021). Thus, the recognition of most potent ALDHs may provide effective defence strategies to cope also with microbes.

## Data availability statement

The datasets presented in this study can be found in online repositories. The names of the repository/repositories and accession number(s) can be found in the article/**Supplementary Material.**

## Author contributions

MA-J, ES-N and JF-W conceived and designed the experiments. YG performed the experiments and most of bioinformatic analysis with assistance of UKT. YG and UKT drafted the manuscript. MA-J, ES-N and JF-W supervised the individual stages of the study, interpreted the results, contributed to writing, and revised the manuscript. All authors contributed to the article and approved the submitted version.

## Funding

This work was supported by grants from the National Science Centre, project no. NCN 2017/26/E/NZ4/00226.

## Conflict of interest

The authors declare that the research was conducted in the absence of any commercial or financial relationships that could be construed as a potential conflict of interest.

## Publisher’s note

All claims expressed in this article are solely those of the authors and do not necessarily represent those of their affiliated organizations, or those of the publisher, the editors and the reviewers. Any product that may be evaluated in this article, or claim that may be made by its manufacturer, is not guaranteed or endorsed by the publisher.
