# Supplementary material for: Comparative genomic analysis of the aldehyde dehydrogenase gene superfamily in Arabidopsis thaliana – searching for the functional key to hypoxia tolerance
Source: Front Plant Sci. 2022 Nov 17;13:1000024. doi: 10.3389/fpls.2022.1000024 (PMC9714362; doi:10.3389/fpls.2022.1000024)

| Family | Gene name | Other names | NCBI gene ID | NCBI protein ID | Phytozome ID | Splice variants | Chrm | AA | Protein molecular weight (kDa) |
| --- | --- | --- | --- | --- | --- | --- | --- | --- | --- |
| ALDH2 | ALDH2B4 | ALDH2; ALDH2A | 823955 | NP_190383.1 | AT3G48000 | 0 | 3 | 538 | 58.60 |
|  | ALDH2B7 | ALDH2B | 838991 | NP_564204.1 | AT1G23800 | 2 | 1 | 534 | 58.16 |
|  | ALDH2C4 | ALDH1A; REF1 | 822042 | NP_566749.1 | AT3G24503 | 0 | 3 | 501 | 54.37 |
| ALDH3 | ALDH3F1 | F23E13.140; F23E13_140 | 829782 | NP_195348.2 | AT4G36250 | 0 | 4 | 484 | 53.62 |
|  | ALDH3H1 | ALDH4 | 841020 | NP_175081.1 | AT1G44170 | 3 | 1 | 484 | 53.16 |
|  | ALDH3I1 | ALDH3 | 829573 | NP_567962.1 | AT4G34240 | 4 | 4 | 550 | 60.18 |
| ALDH5 | ALDH5F1 | ENF1; SSADH; SSADH1 | 844282 | NP_178062.1 | AT1G79440 | 0 | 1 | 528 | 56.57 |
| ALDH6 | ALDH6B2 | MMSD | 815903 | NP_179032.1 | AT2G14170 | 3 | 2 | 607 | 65.94 |
| ALDH7 | ALDH7B4 | F15I1.19; F15I1_19 | 841849 | NP_175812.1 | AT1G54100 | 2 | 1 | 508 | 54.22 |
| ALDH10 | ALDH10A8 | F25A4.11; F25A4_11 | 843831 | NP_001185399.1 | AT1G74920 | 2 | 1 | 496 | 53.98 |
|  | ALDH10A9 | BADH | 823972 | NP_190400.1 | AT3G48170 | 0 | 3 | 503 | 54.92 |
| ALDH11 | ALDH11A3 | F27D4.18; F27D4_18; NP-GAPDH | 816962 | NP_001189589.1 | AT2G24270 | 4 | 2 | 496 | 53.07 |
| ALDH12 | ALDH12A1 | ATP5CDH; K19B1.14; K19B1_14; P5CDH | 836373 | NP_568955.1 | AT5G62530 | 0 | 5 | 556 | 61.78 |
| ALDH18 | ALDH18B1 | TP5CS; T5I7.10; T5I7_10 | 818566 | NP_181510.1 | AT2G39800 | 4 | 2 | 717 | 77.71 |
|  | ALDH18B2 | P5CS2 | 824727 | NP_191120.2 | AT3G55610 | 2 | 3 | 726 | 78.88 |
| ALDH22 | ALDH22A1 | T8E24.4; T8E24_4 | 819849 | NP_974242.1 | AT3G66658 | 2 | 3 | 596 | 66.01 |

Table. S1. *Arabidopsis thaliana* ALDH superfamily.

Table. S2. Primers used in this study.

| Gene | Sequence (5’->3’) | | Accession Number |
| --- | --- | --- | --- |
| *AtALDH2B4* | Forward primer | CTAGAGACATGGGACAATGG | AT3G48000 |
|  | Reverse primer | AGTCCATGAATCTTATCCGC |  |
| *AtALDH2B7* | Forward primer | CCAGTGAAAGTTGAACACAC | AT1G23800 |
|  | Reverse primer | TTCAGACACTTGAGCAATCA |  |
| *AtALDH2C4* | Forward primer | GTGCAGCAGATAAAATCCAC | AT3G24503 |
|  | Reverse primer | GGGAAATTCCAAGGGATGAT |  |
| *AtALDH3F1* | Forward primer | AAATACCGTGCTTCTCAAGT | AT4G36250 |
|  | Reverse primer | ATCAGGTCCTCCTTCGATAA |  |
| *AtALDH3H1* | Forward primer | GACTCGGATACCGATTTGAA | AT1G44170 |
|  | Reverse primer | AAGATATAGTCCGGCGAAAC |  |
| *AtALDH3I1* | Forward primer | CCCAGTTTGAAAAATCGACC | AT4G34240 |
|  | Reverse primer | AACTCTGAGATAACGTCGTG |  |
| *AtALDH5F1* | Forward primer | AAGGTCTAATTGGAGGCAAG | AT1G79440 |
|  | Reverse primer | TTCCCATACATGCAACATCA |  |
| *AtALDH6B2* | Forward primer | TGACGGAAGAGATATTGTGG | AT2G14170 |
|  | Reverse primer | TTGTAACACTCCATGTCTGG |  |
| *AtALDH7B4* | Forward primer | ACACGCATTCCCTTAGTATC | AT1G54100 |
|  | Reverse primer | TTGCATTGTTTCCACTCAAC |  |
| *AtALDH10A8* | Forward primer | TTCTTGTTCACGAAAGCATC | AT1G74920 |
|  | Reverse primer | TTGCTAACTACAGGACCAAG |  |
| *AtALDH10A9* | Forward primer | TGGTCCTGTTGTCAGTAAAG | AT3G48170 |
|  | Reverse primer | TAAATGCTCAGGACGAACTC |  |
| *AtALDH11A3* | Forward primer | TACTGCGAATAAAGGGAGTG | AT2G24270 |
|  | Reverse primer | AACCAAACCTCTCACTCATC |  |
| *AtALDH12A1* | Forward primer | TTTCCGTGTGAGGGAATAAG | AT5G62530 |
|  | Reverse primer | CTGGTGGACTGAGTTTTACA |  |
| *AtALDH18B1* | Forward primer | ATATGGAAGATCCAATCGGC | AT2G39800 |
|  | Reverse primer | GAAGTACGCCTAATGGTGAT |  |
| *AtALDH18B2* | Forward primer | CGACTAAGTTGACTCGTTCT | AT3G55610 |
|  | Reverse primer | CGATACGCTTAACGTCTTTG |  |
| *AtALDH22A1* | Forward primer | CTCATGGGAAAGATACGGAG | AT3G66658 |
|  | Reverse primer | GTGGCAGGTTCATAACATTG |  |
| *Actin-2* | Forward primer | TTGGGATGAACCAGAAGGAT | At3G18780 |
|  | Reverse primer | TCCATATCATCCCAGTTGCT |  |

Table. S4. *Arabidopsis thaliana* ALDH protein superfamily localization.

| Gene name | Localization | Reference |
| --- | --- | --- |
| ALDH2B4 | Mitochondria | Wei et al. (2009) |
| ALDH2B7 | Mitochondria |  |
| ALDH2C4 | Cytosol | Nair et al. (2004) |
| ALDH3F1 | Cytosol | Nair et al. (2011) |
| ALDH3H1 | Cytosol | Nair et al. (2011) |
| ALDH3I1 | Chloroplasts | Sunkar et al. (2003); Kotchoni et al. (2006) |
| ALDH5F1 | Mitochondria | Bouché et al. (2003); Bouché and Fromm (2004); Toyokura et al. (2011) |
| ALDH6B2 | Mitochondria | - |
| ALDH7B4 | Cytosol | Kotchoni et al. (2006) |
| ALDH10A8 | Leucoplasts | Missihoun et al. (2011) |
| ALDH10A9 | Peroxisomes | Missihoun et al. (2011) |
| ALDH11A3 | - |  |
| ALDH12A1 | Mitochondria | Deuschle et al. (2001, 2004); Miller et al. (2009) |
| ALDH18B1 | - |  |
| ALDH18B2 | - |  |
| ALDH22A1 | Cytosol | Krich et al. (2005) |

|  | ALDH2B4 | ALDH2B7 | ALDH2C4 | ALDH3F1 | ALDH3H1 | ALDH3I1 | ALDH5F1 | ALDH6B2 | ALDH7B4 | ALDH10A8 | ALDH10A9 | ALDH11A3 | ALDH12A1 | ALDH18B1 | ALDH18B2 | ALDH22A1 |
| --- | --- | --- | --- | --- | --- | --- | --- | --- | --- | --- | --- | --- | --- | --- | --- | --- |
| ALDH2B4 |  |  |  |  |  |  |  |  |  |  |  |  |  |  |  |  |
| ALDH2B7 | 0,0748631178 |  |  |  |  |  |  |  |  |  |  |  |  |  |  |  |
| ALDH2C4 | 0,7581100652 | 0,6457847964 |  |  |  |  |  |  |  |  |  |  |  |  |  |  |
| ALDH3F1 | 0,6476852116 | 0,4680559594 | 0,7503595256 |  |  |  |  |  |  |  |  |  |  |  |  |  |
| ALDH3H1 | 0,7029452971 | 0,2217818903 | 0,0937698039 | 0,0005916589 |  |  |  |  |  |  |  |  |  |  |  |  |
| ALDH3I1 | 0,3659853653 | 0,6548067857 | 0,1545439050 | 0,0725075196 | 0,0000080073 |  |  |  |  |  |  |  |  |  |  |  |
| ALDH5F1 | 0,0064546770 | 0,4049227517 | 0,0000000000 | 0,2864619838 | 0,5211936780 | 0,8518367934 |  |  |  |  |  |  |  |  |  |  |
| ALDH6B2 | 0,0008234884 | 0,7502807481 | 0,0000000000 | 0,4068152318 | 0,5166255755 | 0,7758042807 | 0,0000093515 |  |  |  |  |  |  |  |  |  |
| ALDH7B4 | 0,0064282148 | 0,0009796858 | 0,0000000000 | 0,6055557855 | 0,2484368541 | 0,1383307152 | 0,0000000000 | 0,0000001807 |  |  |  |  |  |  |  |  |
| ALDH10A8 | 0,0688918701 | 0,0041170748 | 0,0000000000 | 0,7342902406 | 0,0581338239 | 0,0428840250 | 0,0000000000 | 0,0000004422 | 0,0000000299 |  |  |  |  |  |  |  |
| ALDH10A9 | 0,0312153021 | 0,6866566369 | 0,0000000000 | 0,2742937502 | 0,1180396226 | 0,1940114444 | 0,0000000000 | 0,0000479423 | 0,0000000000 | 0,0000000000 |  |  |  |  |  |  |
| ALDH11A3 | 0,0280190340 | 0,5421288455 | 0,0000000000 | 0,7866485865 | 0,8405086515 | 0,8404209614 | 0,0000000070 | 0,0000018723 | 0,0000000483 | 0,0000000000 | 0,0002330063 |  |  |  |  |  |
| ALDH12A1 | 0,0002848217 | 0,8647462978 | 0,0071624157 | 0,9087779503 | 0,4545404221 | 0,2595822008 | 0,0087720526 | 0,0013363144 | 0,0001805334 | 0,0073564969 | 0,0075157256 | 0,0087892843 |  |  |  |  |
| ALDH18B1 | 0,0483819120 | 0,5134339279 | 0,0081017850 | 0,9881065411 | 0,4232049854 | 0,9000673477 | 0,7112620242 | 0,6829958998 | 0,9072712670 | 0,1111250796 | 0,2564578227 | 0,9215577136 | 0,1071841456 |  |  |  |
| ALDH18B2 | 0,5395711814 | 0,4950795390 | 0,1369108808 | 0,2356686916 | 0,0631480235 | 0,0949548207 | 0,4793970344 | 0,9043756190 | 0,6685419088 | 0,3902499540 | 0,9072281154 | 0,6789423449 | 0,5241029971 | 0,8731796332 |  |  |
| ALDH22A1 | 0,0851185769 | 0,6656832385 | 0,3691796711 | 0,9982662159 | 0,9393735787 | 0,4856387053 | 0,0598678011 | 0,3295065292 | 0,1981599679 | 0,8027995797 | 0,6196974428 | 0,4815724809 | 0,9379807695 | 0,2906885086 | 0,5027529552 |  |

Table. S5. Evolutionary constraint (d_N_/d_S_).

Table. S6. All the sequences of used for phylogenetic tree construction.

>AtALDH2B4

MAARRVSSLLSRSFSASSPLLFRSQGRNCYNGGILRRFGTSSAAAEEIINPSVQVSHTQLLINGNFVDSASGKTFPTLDP

RTGEVIAHVAEGDAEDINRAVKAARTAFDEGPWPKMSAYERSRVLLRFADLVEKHSEELASLETWDNGKPYQQSLTAEIP

MFARLFRYYAGWADKIHGLTIPADGNYQVHTLHEPIGVAGQIIPWNFPLLMFAWKVGPALACGNTIVLKTAEQTPLTAFY

AGKLFLEAGLPPGVLNIVSGFGATAGAALASHMDVDKLAFTGSTDTGKVILGLAANSNLKPVTLELGGKSPFIVFEDADI

DKAVELAHFALFFNQGQCCCAGSRTFVHEKVYDEFVEKSKARALKRVVGDPFRKGIEQGPQIDLKQFEKVMKYIKSGIES

NATLECGGDQIGDKGYFIQPTVFSNVKDDMLIAQDEIFGPVQSILKFSDVDEVIKRANETKYGLAAGVFTKNLDTANRVS

RALKAGTVWVNCFDVFDAAIPFGGYKMSGNGREKGIYSLNNYLQIKAVVTALNKPAWI

>AtALDH2B7

MASRRVSSLLSRSFMSSSRSIFSLRGMNRGAQRYSNLAAAVENTITPPVKVEHTQLLIGGRFVDAVSGKTFPTLDPRNGE

VIAQVSEGDAEDVNRAVAAARKAFDEGPWPKMTAYERSKILFRFADLIEKHNDEIAALETWDNGKPYEQSAQIEVPMLAR

VFRYYAGWADKIHGMTMPGDGPHHVQTLHEPIGVAGQIIPWNFPLLMLSWKLGPALACGNTVVLKTAEQTPLSALLVGKL

LHEAGLPDGVVNIVSGFGATAGAAIASHMDVDKVAFTGSTDVGKIILELASKSNLKAVTLELEESHHSFVCEDADVDQAV

ELAHFALFFNQGQCCCAGSRTFVHERVYDEFVEKAKARALKRNVGDPFKSGIEQGPQVDSEQFNKILKYIKHGVEAGATL

QAGGDRLGSKGYYIQPTVFSDVKDDMLIATDEIFGPVQTILKFKDLDEVIARANNSRYGLAAGVFTQNLDTAHRLMRALR

VGTVWINCFDVLDASIPFGGYKMSGIGREKGIYSLNNYLQVKAVVTSLKNPAWL

>AtALDH2C4

MENGKCNGATTVKLPEIKFTKLFINGQFIDAASGKTFETIDPRNGEVIATIAEGDKEDVDLAVNAARYAFDHGPWPRMTG

FERAKLINKFADLIEENIEELAKLDAVDGGKLFQLGKYADIPATAGHFRYNAGAADKIHGETLKMTRQSLFGYTLKEPIG

VVGNIIPWNFPSIMFATKVAPAMAAGCTMVVKPAEQTSLSALFYAHLSKEAGIPDGVLNIVTGFGSTAGAAIASHMDVDK

VSFTGSTDVGRKIMQAAAASNLKKVSLELGGKSPLLIFNDADIDKAADLALLGCFYNKGEICVASSRVFVQEGIYDKVVE

KLVEKAKDWTVGDPFDSTARQGPQVDKRQFEKILSYIEHGKNEGATLLTGGKAIGDKGYFIQPTIFADVTEDMKIYQDEI

FGPVMSLMKFKTVEEGIKCANNTKYGLAAGILSQDIDLINTVSRSIKAGIIWVNCYFGFDLDCPYGGYKMSGNCRESGMD

ALDNYLQTKSVVMPLHNSPWM

>AtALDH3F1

MEAMKETVEESLREMRETFASGRTRSLKWRKAQIGAIYEMVKDNEDKICNALFQDLGKLSTEAFRDELGVVLRTATVAIN

CLDKWAVPKHSKLPLLFYPAKGKVISEPYGTVLVLSSWNFPISLSLDPLIGAIAAGNTVLLKSSELSPNASAFLAKTIPA

YLDTKAIKVIEGGPDVATILLQHQWDKIFFTGSPKIGRIIMAAAAQHLTPVTLELGGKCPTIVDHTISKNIKSVVKRIAG

GKWGSCNGQACISVDYVLIEKSFAPTLIDMLKPTIKSFFGENPKESGCLSRIANKHHVQRLSRLLSDPRVQASIVYGGSI

DEDKLYVEPTILLDPPLDSEIMNEEIFGPILPIITVRDIQESIGIINTKPKPLAIYAFTNDENLKTRILSETSSGSVTFN

DVMIQYMCDALPFGGVGESGIGRYHGKYSFDCFSHEKAIMEGSLGMDLEARYPPWNNFKLTFIRLAFREAYFKLILLMLG

LKR

>AtALDH3H1

MAAKKVFGSAEASNLVTELRRSFDDGVTRGYEWRVTQLKKLMIICDNHEPEIVAALRDDLGKPELESSVYEVSLLRNSIK

LALKQLKNWMAPEKAKTSLTTFPASAEIVSEPLGVVLVISAWNYPFLLSIDPVIGAISAGNAVVLKPSELAPASSALLTK

LLEQYLDPSAVRVVEGAVTETSALLEQKWDKIFYTGSSKIGRVIMAAAAKHLTPVVLELGGKSPVVVDSDTDLKVTVRRI

IVGKWGCNNGQACVSPDYILTTKEYAPKLIDAMKLELEKFYGKNPIESKDMSRIVNSNHFDRLSKLLDEKEVSDKIVYGG

EKDRENLKIAPTILLDVPLDSLIMSEEIFGPLLPILTLNNLEESFDVIRSRPKPLAAYLFTHNKKLKERFAATVSAGGIV

VNDIAVHLALHTLPFGGVGESGMGAYHGKFSFDAFSHKKAVLYRSLFGDSAVRYPPYSRGKLRLLKALVDSNIFDLFKVL

LGLA

>AtALDH3I1

MTKLLEINHIQTLCFAKGFSPARLNVATSPFLISRRGGGGYCSNACIPYRLKFTCYATLSAVVKEQASDFRGKEAALLVD

ELRSNFNSGRTKSYEWRISQLQNIARMIDEKEKCITEALYQDLSKPELEAFLAEISNTKSSCMLAIKELKNWMAPETVKT

SVTTFPSSAQIVSEPLGVVLVISAWNFPFLLSVEPVIGAIAAGNAVVLKPSEIAPAASSLLAKLFSEYLDNTTIRVIEGG

VPETTALLDQKWDKIFFTGGARVARIIMAAAARNLTPVVLELGGKCPALVDSDVNLQVAARRIIAGKWACNSGQACIGVD

YVITTKDFASKLIDALKTELETFFGQNALESKDLSRIVNSFHFKRLESMLKENGVANKIVHGGRITEDKLKISPTILLDV

PEASSMMQEEIFGPLLPIITVQKIEDGFQVIRSKSKPLAAYLFTNNKELEKQFVQDVSAGGITINDTVLHVTVKDLPFGG

VGESGIGAYHGKFSYETFSHKKGVLYRSFSGDADLRYPPYTPKKKMVLKALLSSNMFAAILAFFGFSKDS

>AtALDH5F1

MVIGAAARVAIGGCRKLISSHTSLLLVSSQCRQMSMDAQSVSEKLRSSGLLRTQGLIGGKWLDSYDNKTIKVNNPATGEI

IADVACMGTKETNDAIASSYEAFTSWSRLTAGERSKVLRRWYDLLIAHKEELGQLITLEQGKPLKEAIGEVAYGASFIEY

YAEEAKRVYGDIIPPNLSDRRLLVLKQPVGVVGAITPWNFPLAMITRKVGPALASGCTVVVKPSELTPLTALAAAELALQ

AGVPPGALNVVMGNAPEIGDALLTSPQVRKITFTGSTAVGKKLMAAAAPTVKKVSLELGGNAPSIVFDDADLDVAVKGTL

AAKFRNSGQTCVCANRVLVQDGIYDKFAEAFSEAVQKLEVGDGFRDGTTQGPLINDAAVQKVETFVQDAVSKGAKIIIGG

KRHSLGMTFYEPTVIRDVSDNMIMSKEEIFGPVAPLIRFKTEEDAIRIANDTIAGLAAYIFTNSVQRSWRVFEALEYGLV

GVNEGLISTEVAPFGGVKQSGLGREGSKYGMDEYLEIKYVCLGDMNRH

>AtALDH6B2

MVRVKQKNLESYRSNGTYPPTWRNPTTSFAPDQHRVSIHSSLKSKTKRRRLYKEADDNTKLRSSSSTTTTTTTMLLRISG

NNLRPLRPQFLALRSSWLSTSPEQSTQPQMPPRVPNLIGGSFVESQSSSFIDVINPATQEVVSKVPLTTNEEFKAAVSAA

KQAFPLWRNTPITTRQRVMLKFQELIRKNMDKLAMNITTEQGKTLKDSHGDIFRGLEVVEHACGMATLQMGEYLPNVSNG

VDTYSIREPLGVCAGICPFNFPAMIPLWMFPVAVTCGNTFILKPSEKDPGASVILAELAMEAGLPDGVLNIVHGTNDTVN

AICDDEDIRAVSFVGSNTAGMHIYARAAAKGKRIQSNMGAKNHGLVLPDANIDATLNALLAAGFGAAGQRCMALSTVVFV

GDAKSWEDKLVERAKALKVTCGSEPDADLGPVISKQAKERICRLIQSGVDDGAKLLLDGRDIVVPGYEKGNFIGPTILSG

VTPDMECYKEEIFGPVLVCMQANSFDEAISIINKNKYGNGAAIFTSSGAAARKFQMDIEAGQIGINVPIPVPLPFFSFTG

NKASFAGDLNFYGKAGVDFFTQIKTVTQQWKDIPTSVSLAMPTSQKQ

>AtALDH7B4 A new protein sequence entered manually

MGSANNEYEFLSEIGLTSHNLGSYVAGKWQANGPLVSTLNPANNQPIAQVVEASLEDYEQGLKACEEAAKIWMQVTAPKR

GDIVRQIGDALRSKLDYLGRLLSLEMGKILAEGIGEVQEVIDMCDFAVGLSRQLNGSVIPSERPNHMMLEMWNPLGIVGV

ITAFNFPCAVLGWNACIALVCGNCVVWKGAPTTPLITIAMTKLVAEVLEKNNLPGAIFTAMCGGAEIGEAIAKDTRIPLV

SFTGSSRVGSMVQQTVNARSGKTLLELSGNNAIIVMDDADIQLAARSVLFAAVGTAGQRCTTCRRLLLHESVYDKVLEQL

LTSYKQVKIGNPLEKGTLLGPLHTPESKKNFEKGIEVIKSQGGKILTGGKAVEGEGNFVEPTIIEISADAAVVKEELFAP

VLYVLKFKSFGEAVAINNSVPQGLSSSIFTRNPENIFRWIGPLGSDCGIVNVNIPTNGAEIGGAFGGEKATGGGREAGSD

SWKQYMRRSTCTINYGNELPLAQGINFG

>AtALDH10A8

MAIPMPTRQLFIDGEWREPILKKRIPIVNPATEEVIGDIPAATTEDVDVAVNAARRALSRNKGKDWAKAPGAVRAKYLRA

IAAKVNERKTDLAKLEALDCGKPLDEAVWDMDDVAGCFEFYADLAEGLDAKQKAPVSLPMESFKSYVLKQPLGVVGLITP

WNYPLLMAVWKVAPSLAAGCTAILKPSELASVTCLELADICREVGLPPGVLNVLTGFGSEAGAPLASHPGVDKIAFTGSF

ATGSKVMTAAAQLVKPVSMELGGKSPLIVFDDVDLDKAAEWALFGCFWTNGQICSATSRLLVHESIASEFIEKLVKWSKN

IKISDPMEEGCRLGPVVSKGQYEKILKFISTAKSEGATILHGGSRPEHLEKGFFIEPTIITDVTTSMQIWREEVFGPVLC

VKTFASEDEAIELANDSHYGLGAAVISNDTERCDRISEAFEAGIVWINCSQPCFTQAPWGGVKRSGFGRELGEWGLDNYL

SVKQVTLYTSNDPWGWYKSPN

>AtALDH10A9

MAITVPRRQLFIGGQWTEPVLRKTLPVVNPATEDIIGYIPAATSEDVELAVEAARKAFTRNNGKDWARATGAVRAKYLRA

IAAKVIERKSELANLEAIDCGKPLDEAAWDMDDVAGCFEYYADLAEGLDAKQKTPLSLPMDTFKGYILKEPIGVVGMITP

WNYPLLMAVWKVAPSLAAGCTAILKPSELASLTCLELADICREVGLPPGVLNILTGLGTEAGAPLASHPHVDKIVFTGST

TTGSSIMTSAAKLVKPVSLELGGKSPIIVFDDVDIDKAVEWTMFGCFWTNGQICSATSRLLVHERIADEFLDKLVKWTKN

IKISDPFEEGCRLGPVVSKGQYERVLKFVSNARNEGATVLCGGVRPEHLKKGYFVEPAIVSNVTTSMEIWREEVFGPALC

VKTFSTEDEAIQLANDSQYGLAGAVLSNDLERCDRVSKAFQAGIVWVNCSQPCFCQAPWGGTKRSGFGRELGEWGLENYL

SVKQVTQYISDEPWGWYKPPSKL

>AtALDH11A3

MAGTGLFAEILDGEVYKYYADGEWKTSSSGKSVAIMNPATRKTQYKVQACTQEEVNAVMELAKSAQKSWAKTPLWKRAEL

LHKAAAILKDNKAPMAESLVKEIAKPAKDSVTEVVRSGDLISYCAEEGVRILGEGKFLLSDSFPGNDRTKYCLTSKIPLG

VVLAIPPFNYPVNLAVSKIAPALIAGNSLVLKPPTQGAVSCLHMVHCFHLAGFPKGLISCITGKGSEIGDFLTMHPAVNC

ISFTGGDTGISISKKAGMIPLQMELGGKDACIVLDDADLDLVASNIIKGGFSYSGQRCTAVKVVLVMESVADELVEKVKA

KVAKLTVGPPEENSDITAVVSESSANFIEGLVMDAKEKGATFCQEYKREGNLIWPLLLDNVRPDMRIAWEEPFGPVVPVL

RINSVEEGINHCNASNFGLQGCVFTKDINKAILISDAMETGTVQINSAPARGPDHFPFQGLKDSGIGSQGVTNSINLMTK

VKTTVINLPTPSYSMG

>AtALDH12A1

MYRVFASRALRAKSLCDKSSTSLASLTLSRLNHSIPFATVDAEELSGSHPAEVQSFVQGKWIGSSNHNTLLDPLNGEPFI

KVAEVDESGTQPFVDSLSQCPKHGLHNPFKSPERYLLYGDISTKAAHMLALPKVADFFARLIQRVAPKSYQQAAGEVFVT

RKFLENFCGDQVRFLARSFAIPGNHLGQQSHGYRWPYGPVTIVTPFNFPLEIPLLQLMGALYMGNKPLLKVDSKVSIVME

QMMRLLHYCGLPAEDVDFINSDGKTMNKILLEANPRMTLFTGSSRVAEKLALDLKGRIRLEDAGFDWKVLGPDVQEVDYV

AWQCDQDAYACSGQKCSAQSMLFVHENWSKTPLVSKLKELAERRKLEDLTIGPVLTFTTEAMLEHMENLLQIPGSKLLFG

GKELKNHSIPSIYGALEPTAVYVPIEEILKDNKTYELVTKEIFGPFQIVTEYKKDQLPLVLDALERMHAHLTAAVVSNDP

IFLQEVIGNSVNGTTYAGLRGRTTGAPQNHWFGPAGDPRGAGIGTPEAIKLVWSCHREVIYDYGPVPQGWELPPST

>AtALDH18B1

MEELDRSRAFARDVKRIVVKVGTAVVTGKGGRLALGRLGALCEQLAELNSDGFEVILVSSGAVGLGRQRLRYRQLVNSSF

ADLQKPQTELDGKACAGVGQSSLMAYYETMFDQLDVTAAQLLVNDSSFRDKDFRKQLNETVKSMLDLRVIPIFNENDAIS

TRRAPYQDSSGIFWDNDSLAALLALELKADLLILLSDVEGLYTGPPSDPNSKLIHTFVKEKHQDEITFGDKSRLGRGGMT

AKVKAAVNAAYAGIPVIITSGYSAENIDKVLRGLRVGTLFHQDARLWAPITDSNARDMAVAARESSRKLQALSSEDRKKI

LLDIADALEANVTTIKAENELDVASAQEAGLEESMVARLVMTPGKISSLAASVRKLADMEDPIGRVLKKTEVADGLVLEK

TSSPLGVLLIVFESRPDALVQIASLAIRSGNGLLLKGGKEARRSNAILHKVITDAIPETVGGKLIGLVTSREEIPDLLKL

DDVIDLVIPRGSNKLVTQIKNTTKIPVLGHADGICHVYVDKACDTDMAKRIVSDAKLDYPAACNAMETLLVHKDLEQNAV

LNELIFALQSNGVTLYGGPRASKILNIPEARSFNHEYCAKACTVEVVEDVYGAIDHIHRHGSAHTDCIVTEDHEVAELFL

RQVDSAAVFHNASTRFSDGFRFGLGAEVGVSTGRIHARGPVGVEGLLTTRWIMRGKGQVVDGDNGIVYTHQDIPIQA

>AtALDH18B2

MTEIDRSRAFAKDVKRIVVKVGTAVVTGKGGRLALGRLGAICEQLAELNSDGFEVILVSSGAVGLGRQRLRYRQLVNSSF

ADLQKPQMELDGKACAGVGQSSLMAYYETMFDQLDVTVAQMLVTDSSFRDKDFRKQLSETVKAMLRMRVIPVFNENDAIS

TRRAPYKDSTGIFWDNDSLAALLSLELKADLLILLSDVEGLYTGPPSDSTSKLIHTFIKEKHQDEITFGEKSKLGRGGMT

AKVKAAVNAAYGGVPVIITSGYAAENISKVLRGLRVGTLFHQDAHLWAPVVDTTSRDMAVAARESSRKLQALSSEDRKQI

LHDIANALEVNEKTIKAENDLDVAAAQEAGYEESLVARLVMKPGKISSLAASVRQLAEMEDPIGRVLKKTQVADDLILEK

TSSPIGVLLIVFESRPDALVQIASLAIRSGNGLLLKGGKEARRSNAILHKVITDAIPETVGGKLIGLVTSREEIPDLLKL

DDVIDLVIPRGSNKLVSQIKNSTKIPVLGHADGICHVYVDKSGKLDMAKRIVSDAKLDYPAACNAMETLLVHKDLEQNGF

LDDLIYVLQTKGVTLYGGPRASAKLNIPETKSFHHEYSSKACTVEIVEDVYGAIDHIHQHGSAHTDCIVTEDSEVAEIFL

RQVDSAAVFHNASTRFSDGFRFGLGAEVGISTSRIHARGPVGVEGLLTTRWIMRGKGQVVDGDNGIVYTHKDLPVLQRTE

AVENGI

>AtALDH22A1

MPFWWPLIVLAFAYAICKFLLMLIPPNVPSIDVDASDVLAHGKDTEENSFIYIPPRGRSQQSDKKVQCYEPATMKYLGYF

PALSPTEVEERVTLSRKAQKTWAQSSFKLRRQFLRILLKYIIEHQELICEVSSRDTGKTMVDASLGEIMTTCEKITWLLS

EGERWLKPESRSSGRAMLHKVSRVEFHPLGVIGAIVPWNYPFHNIFNPMLAAVFSGNGIVIKVSEHASWSGCFYFRIIQA

ALAAVGAPENLVDVITGFAETGEALVSSVDKMIFVGSTAVGKMIMRNAAETLTPVTLELGGKDAFIICEDADVSHVAQVA

VRGTLQSSGQNCAGAERFYVHKDIYTAFIGQVTKIVKSVSAGPPLTGRYDMGAICLQEHSEHLQSLVNDALDKGAEIAVR

GSFGHLGEDAVDQYFPPTVLINVNHNMKIMKEEAFGPIMPIMQFSTDEEVIKLANDSRYALGCAVFSGSKHRAKQIASQI

QCGVAAINDFASNYMCQSLPFGGVKDSGFGRFAGIEGLRACCLVKSVVEDRFWPLIKTKIPKPIQYPVAENAFEFQEALV

ETLYGLNIWDRLRSLIDVLKFLTDQSSNVSRTRKSH

>GmALDH2B1

MASSMRISRLLSRSFLSASTTPLFSRGGSGALGAGLSKFSTAAAIEEPIKPPVKVEHTQLLIDGKFVDAATGKTFPTLDP

RTGDVISHVAEGDHEDVDRAVAAARKAFDHGPWPKMTAYERQRILLRAADLFEKHNDELAALETWDNGKPYEQSAQIEIP

MLVRLFRYYAGWADKIHGLTVPADGPYHVQTLHEPIGVAGQIIPWNFPLVMFAWKVGPALACGNTIVLKTAEQTPLSALY

ASKLLHEAGLPPGVLNVISGFGPTAGAAIASHMDIDKLAFTGSTETGKVVLELAARSNLKPVTLELGGKSPFIVCEDADV

DEAVELAHFALFFNQGQCCCAGSRTFVHERVYDEFIEKAKARALKRAVGDPFKGGIEQGPQIDSEQFQKILKYIRSGVES

GATLETGGDRFGNSGFYIQPTVFSNVKDDMLIAKEEIFGPVQTILKFKDLDDVIQRANNTHYGLAAGVFTKNINTANTLT

RALRVGTVWINCFDTFDAAIPFGGYKMSGQGREKGEYSLKNYLQVKAVVTSLKNPAWL

>GmALDH2B2

MASSLRISRLLSRSFLSASTTTPLFSRGGSGALGAGLSKFSTAAAIEEPIKPPLKVEHTQLLIDGKFVDAATGKTFPTLD

PRTGDVISHVAEGDHEDVDRAVAAARKAFDRGPWPKMTAYERQRILLRAADLFEKHNDDLAALETWDNGKPYEQSAQIEI

PMLVRLFRYYAGWADKIHGLTVPADGPYHVQTLHEPIGVAGQIIPWNFPLVMFAWKVGPALACGNTIVLKTAEQTPLSAL

YASKLLHEAGLPPGVLNIISGFGPTAGAAIASHMDIDKLAFTGSTETGKIVLELAARSNLKPVTLELGGKSPFIVCEDAD

VDEAVELAHFALFFNQGQCCCAGSRTFVHERVYDEFIEKAKARALKRAVGDPFKGGIEQGPQIDSEQFQKILKYIRSGVE

SGATLETGGDRFGNSGFYIQPTVFSNVKDDMLIAKEEIFGPVQSILKFKDLDDVIQRANNTHYGLAAGVFTKNINTANTL

TRALRAGTVWVNCFDTFDAAIPFGGYKMSGQGREKGEYSLKNYLQVKAVVTSLKNPAWL

>GmALDH2B3

MATRRLSLLLSRSLSSTSFQAASLLHSLGRNSGKWGNFNRFSTAAAVEDLITPQVPITYTKHLINGQFVDAASGKTFPTY

DPRTGEVIAQVAEGDAEDINRAVSAARKAFDEGPWPKLTAYERCKIILRFADLVEKHGDELAALETWNNGKPYEQSATAE

LPTFVRLFRYYAGWADKIHGLTVPADGNYHVETLHEPIGVAGQIIPWNFPLLMFAWKVGPALACGNTVILKTAEQTPLTA

LYVAKLFHEAGLPPGVLNVVSGYGPTAGAALASHMDVDKLAFTGSTETGKVVLGLAAQSNLKPVTLELGGKSPFIVCEDA

DVDQAVELAHFALFFNQGQCCCAGSRTFVHEHIYDEFLEKAKARALKRVVGDPFKKGVEQGPQIDVEQFQKVLRYIKSGI

ESKATLECGGDQIGSKGFFVQPTVFSNVQDDMLIAKDEIFGPVQTILKFKDIDEVIRRSNATHYGLAAGVFTKNVHTANT

LMRALRVGTVWINCFDVFDAAIPFGGYKMSGIGREKGIYSLNNYLQVKAVVSPVKKPAWL

>GmALDH2B4

MASSLRISRLISRSFSSTSFFSRGGNGFLGSRQSKFSTSAAIEEEPIKPSIQVEHTQLLIDGKFVDAASGKTFQTLDPRT

GEVIAHVAEGHSEDVDRAVSAARKAFDHGPWPKMTAYERQRILLRVADLIEKHNDELAALETWDNGKPYEQAAKIEVPML

VRLIRYYAGWADKIHGLTVPADGPYHVQTLHEPIGVAGQIIPWNFPLLMFAWKVGPALACGNTIVLKTAEQTPLSALYAA

KLFHEAGLPAGVLNVVSGFGPTAGAALASHMEVDKLAFTGSTDTGKVVLELAAKSNLKPVTLELGGKSPFIVCEDADVDQ

AVELAHFALFFNQGQCCCAGSRTFVHENVYEEFVQKAKARALRRVVGDPFKGGIEQGPQIDSDQFEKILRYIRSGVESGA

TLETGGDKLGNKGFYIQPTVFSNVKDGMLIAKDEIFGPVQSILKFKDLGEVVQRANNTRYGLAAGVFTKNMDTANTLTRA

LRVGTVWINCFDTFDAAIPFGGYKMSGQGREKGEYSLKNYLQVKAVVNPLKNPAWL

>GmALDH2B5

MQVMASRILSTLHYVCSSSASATKRCLGLYSHWQRSISGIAASVVADVEPSIAPVQIDQSQLLIDGKFVDAASGKTFPTF

DPRTGDVIANVAEGDAEDVNRAVHAARKAFDEGPWPKMTAYERSRIILRFADLLEKHNDEVAAIETWDSGKTYEQAANVE

IPMVVRLFRYYAGWADKIHGLTVPADGPYHVQTLHEPIGVAGQIVPWNFPLLIFSWKVAPALACGNTVVMKTAEQTPLSA

LYVSKLFLEAGLPPGVLNVISGFGPTAGAALCSHMDVDKLAFTGSTSTGKRVLELSAHSNLKPVTLELGGKSPFIVCKDA

DVDAAVEASHFALFFNQGQCCCAGSRTFVHESIYGEFVEKAKARALKRVVGDPFKNGVEQGPQIDSVQFEKIMKYIRSGV

ESGAQLESGGQRIGSKGYYIQPTVFSNVQDNMLIAKDEIFGPVQSILKFKDLEEVIRRANATSYGLAAGVFTKNMDTANT

LMRALQAGTVWINCYDVFDAAIPFGGYKMSGQGRVRGIYSLRSYLQVKAVVTALKNPAWL

>GmALDH2B6

MLLKGMLKISTRAVSAARKAFDEGPWPKMTAYERSRILLRFADLVEKHSDELAALETWNNGKTYEQAAKTELPMFVRLFH

YYAGWADKIHGLTVPADGDYHVQTLHEPIGVAGQIIPWNFPLVMFAWKVGPALACGNTIVLKTAEQTPLTALFVAKLFHE

AGLPDGVLNVVSGYGPTAGAALASHMDVDKLAFTGSTDTGKVVLELAARSNLKPVTLELGGKSPFIICEDADVDKAVELA

HFALFFNQGQCCCAGSRTFVHERVYDEFLEKSKKRALRRVVGDPFKKGVEQGPQIDVEQFEKVLRYIRSGIESHATLECG

GDRLGSKGFFVQPTVFSNVQDDMLIAQDEIFGPVQSILKFKDIDEVIRRANKTRYGLAAGVFTKNVSTANTLMRALRAGT

VWINCFDVFDAAIPFGGYKMSGIGREKGIYSLHNYLQVKAVVSPVKNPAWL

>GmALDH2B7

MASSLRISRLISRSFSSTSFFSRGGNGFLGSRHCKYSTSSAIEEEPVKPSVQVEHTQLLIDGKFVDAASGKTFPTLDPRT

GEVIAHVAEGHSEDVDRAVAAARKAFDHGPWPKMTAYERQRILLRAADLLEKHNDELAALETWDNGKPYEQAAKIEVPML

VRLIRYYAGWADKIHGLTVPADGPYHVQTLHEPIGVAGQIIPWNFPLLMFAWKVGPALACGNTIVLKTAEQTPLSALYAA

KLFHEAGLPAGVLNVVSGFGPTAGAALASHMEVDKLAFTGSTDTGKVVLELAAKSNLKPVTLELGGKSPFIVCEDADVDQ

AVELAHFALFFNQGQCCCAGSRTFVHESVYDEFVEKAKARALKRVVGDPFKGGIEQGPQIDSDQFEKILRYIRSGVESGA

TLETGGDKLGNKGFYIQPTVFSNVKDGMLIARDEIFGPVQSILKFKDLGEVVQRANNTRYGLAAGVFTTNMDTAYTLTRA

LRVGTVWINCFDTFDAAIPFGGYKMSGQGREKGEYSLKNYLQVKAVVNPLKNPAWL

>GmALDH2B8

MTSIRQCESDESSLKSAFEEVSTFSLCSHWHRSISGIGASAAADVEPSIAPVQIDHSQLLIDGQFVDAASGKTFPTFDPR

TGDVIANVAEGDTEDVNRAVRAARKAFDEGPWPKMTAYERSRIILRFADLLEKHNDEVAAIETWDSGKTYEQAAKVEIPM

VVRLFRYYAGWVDKIHGLTVPADGPYHVQTLHEPIGVAGQIVPWNFPLLIFSWMAAPALACGNTVVIKTSEQAPLSALYV

SKPFLEAGLPPGVLNVITGFGATAGASLCSHMDVDKLAFTGSTSTGKRQSEVTLELGGKSPFIVCEDADVDAAVEAAHFA

LFFNQGQCCCAGSRTFVHESIYDEFVEKAKARALKRVVGDPFKNGVEQGPQIDSAQFEKIMKYIRSGVENGATLESGGQR

IGSKGYYIQPTVFSNVQDNMLIAKDEIFGPVQSILKFKDLEEVIRRANATSYGLASGVFTQNMDTANTLMRALRVGTVWI

NCYDVFDAAIPFGGYKMSGQGRVRGIYSLRSYLQVKAVVTALKNPAWL

>GmALDH2B9

MTFNNGDAAAASLNKVPTVNFTKLFIDGHFVHSVSGKTFETIDPRTGDVIARISEGDKEDIDIAVKAARHAFDNGPWPRL

PGSERGRILLKWAELIEENAEELAALDAIDAGKLYHMCRNLEVPAAANTLRYYAGAADKIHGEVLKMSRDFHAYTLLEPL

GVVGHITPWNFPNTMFYIKVAPSLAAGCTMVLKPAEQTPLSALFNAHLAKLAGIPDGVINVVPGFGPTAGAALSSHMDVD

KVSFTGSTQTGREIMQAAAKSNLKQVSLELGGKSPLIIFDDADIDKAAELALLGILYNKGEVCVASSRVLVQEGIYDEFE

KKLVEKAKAWVVGDPFDPKVQQGPQVDKEQFEKVLSYIEHGKKEGATLLTGGKTVGNKGYFIEPTIFSNIREDMLIAQDE

IFGPVMALKKFKTIEEAIKSANNTKYGLAAGIVTKNLDTANTVSRSIRAGTIWINCYFAFGDDVPFGGYKMSGFGKDHGL

EALHKYLQVKSVVTPLYNSPWL

>GmALDH2C1

MFLSLHIVLINHIATFHLPTPSLRQPPFSLSLARMSALSNSSSSHGNSFLKMPAIKFTKLFINGDFVDSISGRTFETIDP

RKEEVIARVSEGDKEDIDIAVKAARQAFDSGPWPRLPGSERAKIMMKWADLVDENIEELAALDTIDAGKLYYINKVAEIP

SATNALRYYAGAADKIHGDVLKMNGDFHAYTLLEPIGVVGHIIPWNAPSLSFFIKVSPSLAAGCTMVLKPAEQTPLSALF

YAHLAKLAGIPDGVLNIVPGFGPTAGAAISSHMDIDAVSFTGSIEVGREVLQAAAWSNLKPVSLELGGKSPLIIFNDADI

DKASELALFGIMSNKGEICVAGSRVFVQEEIYDEFEKKLVEKAKSWVVGDPFDPKSLQGPQADRNQLEKILSYIEHGKRE

GATLLTGGNTVGNKGYYIEPTIFSNVKEDMLIARDEIFGPVLALMKFKTMEEAIKSANNTKYGLAAGIVTKNLDTANTMS

RSIRAGIVWINCYFTVGSDVPFGGYKMSGFGRDLGLQALHKYLQVKSVVTPIHNSPWL

>GmALDH2C2

MENLSNGHLESFVKIPTIKFTKLFINGEFLDSVSGKTFETVDPRTEEVIAEIAEANKEDVDIAVKAAREAFDCGPWPRMP

GAERAKIMLKWSELIEQNAEEIAALDTIDGGKLFSWCKAVDVPEASNILRYYAGAADKIHGDVFKTSRDLHLYSLMEPVG

VVGHIIPWNFPTVMFFAKVAPALAAGCTMVIKPAEQTPLSSLFYAHLARLAGIPDGVLNVVPGFGSIAGAAISSHMDIDA

VSFTGSTETGRKIMQAAALSNLKPVSLELGGKSPVLIFDDADVDKAVDLALFGILHNKGEICVAFSRVYVQEGIYDEFEK

KVVEKAKTWVVGDPFDPKVQQGPQTSKAQYDKIISYIEHGKSEGATLLTGGKPAGNKGYYIEPTIFVNVKEDMLIAQEEI

FGPVMTLSKFKTIEDAIKKANNSKYGLAAGIVTKNLDIANTVSRSIRAGIIWINCFFAFDIDCPFGGYKMSGFGRDYGLE

ALHKFLKVKSVATPIYDSPWL

>GmALDH2C3

MAALSNGHDASFFKMPSIKFTKLFINGEFVDSLSGKEFETIDPRTGEVITRIAEGAKEDIDVAVKAARDAFDYGPWPRMP

GAERAKIMMKWADLIDQNIEEIAALDAIDAGKLYHWCKAVDIPAAANTIRYYAGAADKIHGEVLKASREFHAYTLLEPIG

VVGHIIPWNFPSTMFVAKVSPSLAAGCTMVLKPAEQTPLSALFYAHLAKLAGIPDGVLNVVPGFGQTAGAAISSHMDIDK

VSFTGSTEVGREVMRAAANSNLKPVSLELGGKSPVIVFDDADVDKAAGLALMGILFNKGEICVAGSRVLVQEGIYDEFEK

KLVEKANAWVVGDPFDPKVQQGPQVDKKQFEKILSYIEHGKKEGATLLTGGKRVGNKGYYIEPTIFSNVKEDMLIVQDEI

FGPVMALMKFKTIEDAIKIANNTRYGLASGIVTKSLDTANTVSRSIRAGIVWINCYFAFGDDIPYGGYKMSGFGRDFGME

ALHKYLQVKSVVTPIYNSPWL

>GmALDH2C4

MTSLTNGDAGSLNKVPTIKFTKLFINGDFVDSLSGKTFETIDPRTGDVIARISEGDKEDIDIAVKAARHAFDNGPWPRLP

GSERARILLKWAEIIEENAEELAALDAIDAGKLYHMCRNVEVPAAANTLRYYAGAADKIHGEVLKMSREFHAYTLLEPLG

VVGHITPWNFPNTMFYIKVAPSLAAGCTMVLKPAEQTPLSALFSAHLAKLAGIPDGVINVVPGFGPTAGAALSSHMDVDK

VSFTGSTQTGRVIMQAAAKSNLKQVSLELGGKSPLIIFDDADIDKATELALLGILYNKGEVCVASSRVFVQEGIYDEFEK

KLVEKAKAWVVGDPFDPKVQQGPQVDKEQFEKVLSYIEHGKKEGATLLTGGKTVGNKGYFIEPTIFSNIREDMLIAQDEI

FGPVMALKKFKTTEEAIKSANNTKYGLAAGIVTKNLDTANTVSRSIRAGTIWINCYFAFGDDVPFGGYKMSGFGKDHGLE

ALHKYLQVKSVVTPLYNSPWL

>GmALDH2C5

MSSLSNNSSSSHGNSFLQMPPIKFTKLFINGDFVDSLSGRTFETIDPRTEEVIARVSEGDKEDIDIAVKAARQAFDSGPW

PRLPASERAKIMMKWADLIDENIEELAALDTVDAGKLNYINKVVEIPSATNALRYYAGAADKIHGEVLKMNGDFHAYTLL

EPIGVVGHIIPWNAPSLSFFIKVSPSLAAGCTMVLKPAEQTPLSALFYAHLAKLAGIPDGVLNIVPGFGPTAGAAISSHM

DIDVVSFTGSIEVGREVMQAAARSNLKPVSLELGGKSPLIIFNDADIDKAAQLALFGIMSNKGEICVASSRVFVQEEIYD

EFEKKLVEKAKSWVVGDPFDPKSLQGPQADRNQLEKILSYIEHGKREGATLLTGGNTVGNKGYYIEPTIFCNVKEDMLIA

RDEIFGPVLALMKFKTMEEAIKSANNTKYGLAAGIVTKNLDTANTMSRSIRAGIVWINCYLTVGSDVPFGGYKMSGFGRD

LGLQALHKYLQVKSVVTPIHNSPWL

>GmALDH2C6

MNSNGYPASSFKIPTVKFTKLFINGHFVDSLSGGEFETIDPRTGEVIARIAEGTKEDIDLAVKASRLAFDHGPWPRMPAV

ERARIMMKWADLIDQHVEEIAALDAIDAGKLYHMLKAIEIPATANTIRYYAGAADKIHGEVLKPAREFHAYTLLEPVGVV

GHIIPWNFPSIMFVSKVSPCLAAGCTMVLKPAEQTPLSALFYAHLAKLAGIPDGVLNVVPGFGATAGAAICSDMDIDKVS

FTGSTEVGREVMRAAANSNLKPVSLELGGKSPFIIFDDADLDKAVELALMAVVYNKQQHIFISDNYLLLLSGFQGEVCAA

GSRVFVQEGIYDEFEKRLVEKAKAWVVGDPFDPNVQQGPQVDKKQFEKILSYIEHGKREGATLLTGGKRVGNKGYYIEPT

IFSNVKEDMLIAQDEIFGPVIALMKFKTIEEAIKSANNSRYGLVAGVVTKSLDTANTMSRSIRAGVVWINCYFAFENDIP

YGGCKMSGFGKDSGLEALHKYLHVKSVVTPIYNSPWL

>GmALDH2C7

MAALSNGHGSSFFKMPPIKFTKLFINGEFVDSLSGREFETRDPRTGEVITRIAEGAKEDVDVAVKAARAAFDYGPWPRMP

GAERAKIMMKWADLVDQNIEEIAALDAIDAGKLYHWCKAVDIPAAASTIRYYAGAADKIHGEVLKASREFHAYTLLEPIG

VVGHIIPWNFPSTMFVAKVSPSLAAGCTMVLKPAEQTPLSALFYAHLAKLAGIPDGVLNVVPGFGQTAGVAISLHMDIDK

VSFTGSTEVGREVMRAAANSNLKPVSLELGGKSPVIVFDDADVDKAAELALLGILFNKGEICVAGSRVLVQEGIYDEFEK

KLVEKAKAWVVGDPFDPKVQQGPQVDKKQFEKILSYIEQGKKEGATLLTGGKRVGNKGYYIEPTIFSNVKEDMLIVQDEI

FGPVMALMKFKTIEDAIKIANNTRYGLASGIVTKSLDTANTVSRSIRAGIVWINCYFAFGNDIPYGGYKMSGFGRDFGME

ALHKYLQVKSVVTPIYNSPWL

>GmALDH2C8

MANLSNSHSESFVKIPTVKFAKLFINGEFLDSVSGKTFETVDPRTEEVIAEIAEANKEDVDIAVKAAREAFDFGPWPRIP

GAERAKIMLKWSQLIEQNAEEIAALDTIDGGKLFSWCKAVDVPEASNILRYYAGAADKIHGDVFKTSRNLHLYSLMEPVG

VVGHIIPWNFPTVMFFAKVAPALAAGCTVVIKPSEQTPLSSLFYAHLSKLAGIPDGVLNVVPGFGSIAGAAISSHMDIDA

VSFTGSTETGRKIMQAAALSNLKPVSLELGGKSPLLIFDDADVDKAVDLALFGILHNKGEICVAFSRVYVQKGIYDEFEK

KVVEKAKTWVVGDPFDPKVQQGPQTSKAQYDKILSYIEHGKSEGATLLTGGNPAGNKGYYIEPTIFANVKEDMLIAQEEI

FGPVMTLSKFKTIEDGIKKANSSKYGLAAGIVTKNLDIANTVSRSIRAGIIWINCFFAFDIDCPFGGYKMSGFGRDYGLE

ALHKFLKVKSVATPIYNSPWL

>GmALDH2C9

MAVFNSLHKTSTRFFVPTGRNSGKWGNVNRFSTAAAVEELIIPQVPITYTKHLINGQFVDADAASGKTFPTYDPRTGEVI

ARVAEGDAEDINRAVSAARKAFDEGPWPKMTAYERCQIILRFADLTWNNGKPYEQWATSELPTFVRLFRYYAADKIHGLT

VPADGNYHVETLHEPIGVAGQIIPWNFPLLMFAWKVGPALACGNTVILKTAEQTPLTALYVAKAGLPPGVLNVVSGYGPT

AGAALASHMDVDKLAFTGSTETGKVVLELAARSNLKPVILNLEGNLLSLGQCCCAGSRTFVHERIYDEFLEKAKARALKR

VVGDPFIKGVEQGPQVCFASTLRQNIIDCVLSYHFCSFYSYYKATLECGGDRIGSKGFFVQPTVFSNVQRVGTVWINCFD

VFDAAIPFGGYKMSGISREKGIYSLNNYLQVKAVVSPVKNPAWL

>GmALDH3F1

MDIGGGVEEPVRELRQYFKTGKTKSVTWRKNQLTSLIDLVHENEDAIFKALHKDLGKHPVEAYRDEVGGVEKSASKALSC

VEKWMAPKKSDIPFLFFPAKGEVLSEPLGVVLIISSWNFPIILALDPIIGAISAGNVVVIKPSEQAPACSSFLANTIPRY

LDSNAIKVIEGGEDVCEQLLRQKWDKIFFTGSPRVASVVMSAAAKNLTPVTLELGGKCPAILDSLPNPSEFELAVKRIVG

GKWGPCSGQACIGIDYLLVEEKFSSAVIKLLKKFIRRFYGENPVESKVISRIINKQHFERLCNLLKDPLVAASIVHGGSV

DEENLFIEPTILLDPPLDSEIMAEEIFGPLLPIITLDKIQESIEFINAKPKPLAIYAFTKDETFKRKILSETSSGSVVFN

DTMVQFLCDTLPFGGVGQSGLGRYHGKYSFDTFSHEKAVMHRKLFLEIEPRYPPWNKFKLEFIRLAYRLNYFGLVLHMLG

LKRYN

>GmALDH3F2

MSGEETQRNVFGAETASSLVKELRDNFGKGTTRSYEWRVSQVKALLKAVVENEDQIVGALCSDLAKPPLETVVYEIGMFQ

NSCEVILKELKHWMTPEKVKTSIRTFPSSAEIVPEPLGVVLVISAWNYPILLSLDPVVGAIAAGNAVVLKPSEIAPATSS

VLAKLIEKYMDNSFVRVVEGAVDETTALLQQKWNKIFYTGNGRVGKIVMTAAAKHLTPVVLELGGKSPVVVDSNNNLLVA

ARRIIAGKWGLNNGQACISPDYVITTKDYAPKLVDTLKTELESFYGRNPLESEDLSRIVSSNHFARLSKLLNDDKVSGKI

VYGGEKDEKKLRIAPTILLDVPQDSSIMGEEIFGPLLPIITVNKLEESIDVINSGAKPLAAYVFTTDNKFKEQFVKNVSA

GGLLVNDTALHLVVDTLPFGGVGESGMGAYHGKFSFDAFTHKKAVLYRSFAGDSAIRYPPYTDTKLRLMKALVGGRILGI

IRALFGWS

>GmALDH3H1

MSVEEMQSQKRNVFDAETASSLVKELRDNFGSGRTRSYEWRVSQVKALLKAVVDNEEQIVDALRSDLAKPPLETIVYEVG

MFKNSCEVILKELKQWMKPEKVKTSIRTFPSSAEIVPEPLGVVLVISAWNYPILLSLDPVVGAIAAGNAVVLKPSEIAPA

SSSLLLKLIEKYCDNSFIRVVEGAVDETTALLQQKWDKIFYTGNGKVGRIVMTAAAKHLTPVVLELGGKSPVVVDSNVDL

QIAARRIISGKWGLNNGQACISPDYVITTKDCAPKLVDALKTELEKCYGKNPLESEDLSRIVTSNHFARLSKLLDDDKVA

GKIVYGGEKDEKKLRIAPTLLLDVPRDSLIMGEEIFGPLLPIITVNKVEESIDLINSGTKPLAAYIFTTNKKLKEQFVMN

VPAGGLLVNDTVLHLVVDTLPFGGVGESGMGAYHGKFSFDAFTHKKAVLYRSFAGDSSLRYPPYTDTKLRLMKALIGGRF

LGIIRALFGWS

>GmALDH3H2

MSSTPQDSVKTTASAKNTAFDAEAASRLVNELRRNFASNKTRSYEWRLSQLNALEKLVVVHEQEIVDALRNDLGKPPLET

VAYEIAMLKNSCRIALKELKHWMTPEKVKTSIATFPSSAEIVSEPLGVVLVISAWNYPFLLSLDPVVGAIAAGNAVVLKP

SEIAPATSSLLAKLIGDYLDNSCIRVVEGAVDETSALLQQKWDKIFYTGNGRVARIVMAAASKHLTPVVLELGGKSPVVV

DSNINLKVATRRIIAGKWGSNNGQACISPDYIITTKDYAPKLVDALKTELEKFYGKNPLESKDLSRVVNSNHFNRLTKLL

DDDKVSGKIVYGGQKDENKLKISPTVLLDVPRDSLIMNEEIFGPLLPILTVDKLEESFDVINSGPKPLAAYIFTNNKKLK

EQFVMTISAGGLVVNDTTLHLAVHTLPFGGVGESGVGAYHGKFSFEAFSHKKAVLYRKFIGDAPVRYPPYTNTKMRLLKA

IIGGGIHGIVRALFGW

>GmALDH3H3

MSSSTPDSDKTTTSSKKSAFDALAASRLVTELRGNFASGKTRSYEWRLLQLNAIAKLVVDHEQEIVDALRNDLGKPPLET

VAYEIAMLKNSCRIALKELKHWMTPEKVKTSIATFPSSAEIVSEPLGVVLVISAWNYPFLLSLDPVIGAIAAGNAVVLKP

SEIAPATSSLLAKLLGDYLDNSCIKVVEGAVDETSALLQQKWDKIFYTGNGRVARIVMAAASKHLTPVVLELGGKSPVVV

DSNINLKVATRRIIAGKWGSNNGQACISPDYIITTKDYAPKLVDALKTELEKFYGKNPLESKDLSRIVNSNHFNRLTKLL

DDDKVSGKIVYGGEKDESKLKISPTVLLDVPRDSLIMNEEIFGPLLPILTVDKIEESFDVINSGSKPLAAYIFTNTKKLK

EQFVMTISAGGLVVNDTTLHLAVHTLPFGGVGESGVGAYHGKFTFEAFSHKKAVLYRRFIGDAPVRYPPYTNTKMRLLKA

LIGGGILGIIRALFGW

>GmALDH3H4

MKSLCLGPFLAASAPVGRRAYGGHLSRKCFQKQLHFHSRCVAFSSFICSATISVMPELEEKQVFDGEKANLLVKDLRKSF

DSGMTKSYGWRVSQLEAIAKMLEEKEKEITEALYKDLGKPRLEAFITEISQAKSSCSEALKELKEWMKPEKVNTSITTYP

SSAEIVPEPLGVVLVISTWNFPFLLSMDPVIGAISAGNAVVLKPSEISPATSSLLANLIEQYLDNSTIRVVEGAIPETSA

LLDQKWDKILYTGSARVGRIVMAAAAKHLTPVILELGGKCPAVVESDVNLQVTARRIIAGKWACNSGQACISVDYIITRK

EFAPKLVDALKEELEQFFGKDPMESKDMSRIVSPNQFARLVNLLDEDKVSDKIVLGGQRDEKKLKIAPTIILGVPEDAMI

MQEEIFGPIMPIVTVDNIEDCYSIIKSKPKPLAAYLFTNNEQLKKDYVDKISSGGMLINDAVIHVATRGLPFGGVEESGM

GCYHGKFSFDSFSHRKSVLYRSFDADSTIRYPPYTPQKEKLLKALISGNIVQIILSLLGWS

>GmALDH3I1

MEITMQTLERDLNDTRGYYESGKTKEESWRESQLKGLRRFLLEKQVDIMNALMHDLGKHQLEAFRDEIGTLIKTVNLALK

SLKDWMSGKKAALPQLALLTSAEIVPEPLGLVLIISSWNFPIGISLEPLIGAVAAGNAAVLKPSELSPACSSLLASSLPT

YLDDKAIKVIQGGPQETQQLLEQRWDKIFFTGSARVGRIVMSSAVKHLTPVTLELGGKCPAVVDSLSSSWDKEVTVKRII

VGKYGTCAGQACITIDYVLVEKGYCLKLVELMKVWIKKMFGQNPRKSKTIAKIVNKHHFSRLKNLLADKQVKGSVVYGGS

MDEQNLFIEPTILVDPPLEAAIMSEEIFGPLLPIITVEKIEDSIKFINARPKPLALYVFTKNHTLQRRMISETSSGSVTI

NDAVLQYAADTIPFGGVGESGFGMYHGKFSFDTFSHQKAIVRRSFLTDFWYRYPPWTLNKLQLLEVSYNYDYLGLLLVLL

GLKRPSKRLIADHV

>GmALDH3J1

MEIIMPSLERDLNDTRGYYESGKTKEASWRESQLKGLRRFLIEKQEDIMNALMHDLGKHQLEAFRDEIGTLIKTLNLALK

SLKHWMSGKKAALPQLALLTSAEIVPEPLGVVLIISSWNFPFGISLEPLIGAVAAGNAAVLKPSELSPACSSLLASNLST

YLDNKAIKVIQGGPKETQQLLEQRWDKIFFTGSAHVGKIVMSAAVKHLTPVTLELGGKCPAVVDSLSSSWNIEVAVKRII

VGKYGACAGQACIAIDYVLVEKVYCFKLVELMKVWIKKMCGENPQQSKTIAKIVNKHHFSRLKNLLADKKVKESVIYGGS

MDEQNLFIEPTILVDPPLEAAIMSEEIFGPLLPIITVEKIEDSIKFINSRPKPLALYVFTKNQTLQRRMISETSSGSVTI

NDAILQYAVDTVPFGGVGESGFGMYHGKFSFDTFSHQKAIVRRSFLTDFWYRYPPWTLNKLQLLEVSYNYDYLGLLLVLL

GLKRPSKRLISDHV

>GmALDH3J2

MKYTGEALGRDLENVRKYYGSGKTKEASWRESQLKGLHNFLVEKEEEILRALKHDLGKHYVEAFRDEVGTLMKTLNLASK

SLKNWMAGKEAKLPRIALLSSAEIVPEPLGLVLIISSWNFPFGLSLEPLIGAIAAGNSVVLKPSELSPTCSSLLATFLPT

YLDNNAIKVIQGGPEVGELLLQQRWDKIFFTGSARVGRIVMSAAAVHLTPVTLELGGKCPAIIDSLSSSWDKEVAVKRIL

VAKFGACGGQACIAIDYVLVEKSFSSTLVTLMKEWIKKLFGENPKVSNTIARIVNKNHFMRLKNLLTEPRVKESVVYGGS

MDENDLFIEPTILLDPPLDSAIMAEEIFGPVLPIITVEKIEESVEFISSRPKALAIYAFTKNQTLQRRLVSETSSGSLVF

NDAILQYVADTLPFGGVGECGFGKYHGKFSFDAFSHHKAVARRSYLTDFWFRFPPWTLNKLQLLEVSYNLDYLGILLVLL

GLKKSKRSLFQACN

>GmALDH3J3

MEYSVETLERDLKNTRKYYGSGKTKEAPWRESQLKGLHNFLVEKEEEIVTALKHDLGKHYVEAFRDELGTLMKTLNLATK

SLKNWMAGKEAKLPRIALLSSAEIVPEPLGLVLIISSWNFPFGLSLEPLIGAVAAGNSVVLKPSELSPTCSSLLATFLPT

YLDNNAIKVIQGGPEVGKLLLQQRWDKIFFTGSARVGRIVMSAAAVHLTPVTLELGGKCPALIDSLSSSWDKEVAVKRIL

VAKFGSCAGQACIAIDYVLVEKSFSSTLVTLMKEWIKKMFGENPKASNSIARIVNKNHFMRLQNLLTEPRVKESVVYGGS

MDENDLFIEPTILLDPPLDSAVMAEEIFGPVLPIITLEKIEDSVEFISSRPKALAIYAFTKNQTLQRRMVSETSSGSLVF

NDAILQYVADTLPFGGVGECGFGKYHGKFSFDAFSHHKAVARRSYLTDFWFRFPPWTLDKLQLLEVSYNLDYLGILLVLL

GLKKSKRSLFQACN

>GmALDH3J4

MRPPHYKYSCPHEWEGSVEENKKEKENNQYDNFAILVVDQGRSIAMDIGGEVEETVRELRQYFKTGKTKSVTWRKNQLTA

LLDLVHENEDAIFKALHQDLGKHPVEAYRDEVGGVEKSASNALSCVEKWMAPKKSDIPFLFFPAKGEVLSEPLGVVLIFS

SWNFPIILTLDPIIGAISAGNVVVIKPSEQSPASSSFLATTIPRYLDSNAIKVIEGGPDVCEQLLLQKWDKIFFTGSPRV

ASVVMSAAAKNLTPVTLELGGKCPAILDSLPNPLEFKLAVKRIVGGKWGPCSGQACIAIDYLLVEKKFSYALIELLKKII

RRFYGENPVESKVISRILNKQHFERLCNLLKDPLVAASIVHGGSVDEENLFIEPTILLDPPLDSQIMSEEIFGPLLPIIT

MDKIQESIEFINAKPKPLAIYAFTKDETFKRNILSETSSGSVVFNDTMVQFLCDTLPFGGVGQSGFGRYHGKYSFDTFSH

EKAVMHRKLFLEIEPRYPPWSKFKLEFIRLAYRLNYFGLLLHMLGLKRYK

>GmALDH5F1

MAALNLCRMALRSSKLLYRPYNLLSVQLQMQMQPSSPPLTRKMSTDAQSIASQLNSSGLLRTQGLIAGKWSDAYDGKTIK

VYNPATGESVVDVACMGGRETNDAISAAYDAYGSWSKTTAAERSKLLRKWYDLLMVHKEELAQLITLEQGKPLKESVGEI

VYGAGFIEFAAEEAKRIYGDIVPAPFSDRRLFVLKQPVGVVGAITPWNFPLAMITRKVGPALACGCTVVIKPSELTPLTA

LAAVELSIQAGIPPGVVNVVMGNAPDIGDALLASPQVRKITFTGSTAVGKKLMAGSAETVKKVSLELGGNAPCIVFDDAD

LDVAVKGTLAAKFRNSGQTCVCANRIIVQEGIYEKFANALRDAVQNMKVGDGFSEGVSQGPLINEAAVKKVESLIHDATS

KGAKVILGGKRHSLGLTFYEPTVISDVNSDMHISREEAFGPVAPLLRFKTEEEAIRIANDTNAGLGSYVFTNSIQRSWRV

AEALEYGLVGVNEGVISTEVAPFGGFKQSGLGREGSKYGMDEYLEIKYVCFGNMNKE

>GmALDH5F2

MAALNLCRMALRSSKLLSRPYHRLSVQLQMQMQPSSPPLTRKMSMDAQSVASQLNSSGLLRTQGLIGGKWSDAYDGKTIK

VYNPATGESIVDVACMGGRETNDAISAAYDAYGSWSKTTAAERSKFLRKWYDLLMVHKEELAQLITLEQGKPLKESVGEI

NYGAGFIEFAAEEAKRIYGDIIPAPLSDRRLFVLKQPVGVVGAITPWNFPLAMITRKVGPALACGCTVVIKPSELTPLTA

LAAAELSIQAGIPPGVVNVVMGNAPDIGDALLASPQVRKITFTGSTAVGKKLMAGSAETVKKVSLELGGNAPCIVFDDAD

LDVAVKGTLAAKFRNSGQTCVCANRIIVQEGIYEKFANALRDTVQNMKVGDGFSEGVAQGPLINEAAVKKVESLIHDATS

KGAKVILGGKRHSLGFTFYEPTVISDVNSDMRISREEAFGPVAPLLRFKTEEDAIRIANDTNAGLGSYIFTNSIQRSWRV

AEALEYGLVGVNEGVISTEVAPFGGFKQSGLGREGSKYGMDEYLEIKYVCLGNMHKA

>GmALDH6B1

MLRLSIQRVRKLNFLSPQISALGRSHLSTAAEPSSSKSNPPRVPNLIGGSFVDSKASTVIDVINPATQEVVSQVPLSTDE

EFKEAVSAAKKAFPSWRNTPITTRQRVMLKLQELIRRDMDKLALNVTTEQGKTLKDAQGDVFRGLEVVEHACGMATLQMG

EYVSNVSHGIDTYSIREPLGVCAGICPFNFPAMIPLWMFPMAITCGNTFVLKPSEKDPGASVMLAELALEAGLPEGVLNI

VHGTHDIVNAICDDDDIKAISFVGSNVAGMHIYSRAAAKGKRVQSNMGAKNHAIVMADANVDATLNALVAAGFGAAGQRC

MALSTVVFVGGSKPWEDKLLEHAKALKVNAGTEPDTDLGPVISKQAKERIHRLVQSGVESGARLLLDGRNIVVPGYESGN

FIGPTILSDINANMECYKEEIFGPVLLFMEADSLEEAINIINSNKYGNGASIFTTSGVAARKFQTEIEAGQVGINVPIPV

PLPFFSFTGNKASFAGDLNFYGKAGVNFYTQIKTITQQWKDSTGGSKINLAMPTSQK

>GmALDH6B2

METRMLRLSIQRVRKLNFLRPQISALGRSHLSTAAEPSSSKSNPPRVPNLIGGSFVDSKASTVIDVINPATQEVVSQVPL

STHEEFKAAVSAAKEAFPSWRNTPITTRQRVMLKLQELIRRDMDKLALNVTTEQGKTLKDAQGDVFRGLEVVEHACGMAT

LQMGEYVSNVSHGIDTYSIREPLGVCAGICPFNFPAMIPLWMFPMAVTCGNTFVLKPSEKDPGASVMLAELALEAGLPEG

VLNIVHGTHDIVNAICDDENIKAISFVGSNVAGMHIYSRAAAKGKRVQSNMGAKNHAIVMPDANVDATLNALVASGFGAA

GQRCMALSTVVFVGGSKPWEDKLLERAKALKVNAGTEPDTDLGPVISKQAKERIHRLVQSGVESGARLLLDGRNIVVPGY

ESGNFIGPTILSDINANMECYKEEIFGPVLLFMEADSLEEAINIINSNKYGNGASIFTTSGVAARKFQTEIEAGQVGINV

PIPVPLPFFSFTGNKASFAGDLNFYGKAGVNFYTQIKTITQQWKDSTGGSRINLAMPTSQK

>GmALDH6B3

MANSHLSTPSELFSRQHKPPRVPNLIGGSFLDSKSLTFIDVINPATQEVVSQVPCTTDEEFKAAVSAAKKAFPSWRKTPI

TKRQRVMLKFQELIRRDMDKLALNVTTEQGKTLKDAQGDVFRGLEVVEHACGMATLQMGEYVSDVSSGIDTYSIREPLGV

CAGICPFNFPAMIPLWMFPVAVTCGNTFILKPSEKVPGASVMLAELAMEAGLPEGVLNIVHGTHDIVNAICDDDDIKAIS

FVGSNVAGMHIYARAAAKGKRVQANMGAKNHAVVMPDASVDATVNALVAAGFGAAGQRCMALSTVVFVGDSKLWESKLVE

HAKALKVNVGTEPDADLGPVISKQAKERIHRLIQSGVESGARLVLDGRNIVVPGYESGNFIGPTILSDVTANMECYKEEI

FGPVLLLTEADNLEEAINIINENKYGNGASIFTTSGVAARKFQTEIEAGQVGINVPIPVPLPFFSFTGNKASFAGDLNFY

GKAGVNFYTQIKTVTQQWKDSASESKINLAMPTSQKS

>GmALDH7B1

MGSDNHQNLEFLKEIGLGSSNIGSYINGQWKATGSSVTSVNPSNNQSIAQVTEATLQDFEEGLRACSEAAKTWMTIPAPK

RGEIVRQIGEALRAKLDPLGRLVSLEMGKILPEGIGEVQEIIDMCDYCVGLSRQLNGSIIPSERPDHMMFEVWNPLGIVG

VISAFNFPCAVLGWNACIALVCGNCVVWKGAPTTPLITIAVTKLVAEVLERNKLPGAIFTSFCGGADIGQAIAKDTRIPL

VSFTGSSKVGLMVQQTVNERFGKCLLELSGNNAIIVMDDADIKLAVRSILFAAVGTTGQRCTTCRRLFLHESIYTDVLDQ

LVEVYKQVKIGNPLEKGTLVGPLHTRTSVENFQKGISVIKSQGGKILTGGSVLESGGNFVQPTIVEISPDAPVVKEELFG

PVLYVMKFQTLEEAIALNNSVPQGLSSSIFTQRPGTIFKWIGPRGSDCGIVNANIPTNGAEIGGAFGGEKATGGGREAGS

DSWKQYMRRSTCTINYGSELPLAQGINFG

>GmALDH7B2

MGSDNTNLEFLKEIGLGSSNIGSYINGQWKATGSSVTSVNPSNNQSIAQVTEATLQDYEEGLQACSEAAKTWMTIPAPKR

GEIVRQIGEALRAKLDPLGRLVSLEMGKILPEGIGEVQEIIDMCDYCVGLSRQLNGSIIPSERPDHMMFEVWNPLGIVGV

ITAFNFPCAVLGWNACIALVCGNCVVWKGAPTTPLITIAVTKLVAEVLERNKLPGAIFTSFCGGADIGQAIAKDTRIPLV

SFTGSSKVGLMVQQTVNERFGKCLLELSGNNAIIVMDDADIKLAVRSILFAAVGTAGQRCTTCRRLFLHESIYADVLDQL

IGVYKQVKIGNPLEKGTLVGPLHTPTSVENFQKGISVIKSQGGKILTGGSVLESAGNFVQPTIVEISPDAPVVKEELFGP

VLYVMKFQTLEEAIALNNSVPQGLSSSIFTQRPGTIFKWIGPRGSDCGIVNANIPTNGAEIGGAFGGEKATGGGREAGSD

SWKQYMRRSTCTINYGSELPLAQGINFG

>GmALDH10A1

MSIPIPHRQLFIDGDWKVPVLKNRIPIINPSTQHIIGDIPAATKEDVDLAVAAAKAALSRNKGADWASASGSVRARYLRA

IAAKITEKKPELAKLEAIDCGKPLDEAAWDIDDVAGCFEFYADLAEKLDAQQKAHVSLPMDTFKSYVLKEPIGVVALITP

WNYPLLMATWKVAPALAAGCAAILKPSELASVTCLELAEICKEVGLPPGVLNILTGLGPEAGAPLAAHPDVDKIAFTGSS

ATGSKIMTAAAQLIKPVSLELGGKSPIIVFEDVDLDKAAEWTIFGCFWTNGQICSATSRLIESIATEFLNRIVKWVKNIK

ISDPLEEGCRLGPIVSEGQYEKILKFISNAKSEGATILTGGSRPEHLKKGFFVDQLEEVFGPVLCVKTFSTEEEAIDLAN

DTVYGLGSAVISNDLERCERITKAFKAGIVWINCSQPCFTQAPWGGIKRSGFGRELGEWGLDNYLSVKQVTQYISDEPWG

WYQSPSRL

>GmALDH10A2

MAISIPSRQLFIDGEWKVPLLNNRFPIINPATEDIIGHIPAATKEDVDLAVDAAKRAFSHNKGKDWSSAPGSVRARYLRA

IASKITEKKDELGKLEAIDCGKPLDEALADLDDVIGCFNYYAELAEGLDAKQNAPVSLPMETFKSYVLKEPIGVVALITP

WNYPLLMATWKVAPALAAGCTAILKPSELASVTCLELAEICREVGLPPGVLNIVTGLGNEAGAPLSSHPDVDKISFTGSS

ATGSRIMTAAAQLTKPVSLELGGKSPIIVFEDVDLDKTAEWTIFGCFFTNGQICSATSRLIVHESIATEFVNRLVQWAKN

IKISDPFEEGCRLGPIVSEGQYKKVLNCISTAKSEGATILIGGSRPEHLKKGYFVEPTIITDVTTSMQIWREEVFGPVLC

VKTFSTEEEAIELANDTHYGLGSAVMSKDLERCERISKAIQAGIVWINCAQPSFIQAPWGGVKRSGFGRELGEWGLENYL

SVKQVTKYISDEPWGWYQSPSKL

>GmALDH11A1

MAAGTGLFAEILDGDAYKYYADGEWKKSASGKSVSIINPTTRKTQYKVQACSQEEVNKVMDLAKSAQKLWAKTPLWKRAE

LLHKAAAILKEHKTPIAECLVKEIAKPAKDAVMEVVRSGDLVSYTAEEGVRILGEGKFLVSDSFPGNERTKYCLTSKIPL

GVILAIPPFNYPVNLAVSKIAPALIAGNSIVLKPPTQGAVSALHMVHCFHLAGFPKGLINCVTGKGSEIGDFLTMHPGVN

CISFTGGDTGISISKKAGMIPLQMELGGKDACIVLEDADLDLVAANIIKGGFSYSGQRCTAVKVVLVMESVADALVEKVK

AKVAKLTVGPPEDDCDITPVVSESSANFIEGLVLDAKEKGATFCQEYKREGNLIWPLLLDNVRPDMRIAWEEPFGPVLPV

IRINSVEEGIHHCNASNFGLQGCVFTKDVNKAIMISDAMETGTVQINSAPARGPDHFPFQGIKDSGIGSQGITNSINMMT

KVKTTVINLPSPSYTMG

>GmALDH11A2

MAAGTGLFAEILDGDVYKYYADGEWKKSASGKSVAIINPTTRKTQYKVQACSQEEVNKVMDLAKSAQKLWAKTPLWKRAE

LLHKAAAILKEHKAPIAECLVKEIAKPAKDAVTEVVRSGDLVSYTAEEGVRILGEGKFLVSDSFPGNERTKYCLTSKIPL

GVILAIPPFNYPVNLAVSKIAPALIAGNSIVLKPPTQGAVSALHMVHCFHLAGFPKGLINCVTGKGSEIGDFLTMHPGVN

CISFTGGDTGIAISKKAGMIPLQMELGGKDACIVLEDADLDLVAANIIKGGFSYSGQRCTAVKVVLVMESAADALVEKVK

AKVAKLTVGPPEDDCDITPVVSESSANFIEGLVLDAKEKGATFCQEYKREGNLIWPLLLDNVRPDMRIAWEEPFGPVLPV

IRINSVEEGIHHCNASNFGLQGCVFTKDVNKAIMISDAMETGTVQINSAPARGPDHFPFQGIKDSGIGSQGITNSINMMT

KVKTTVINLPSPSYTMG

>GmALDH11A3

MAGSGTFAEIIDGDVFKYYAQGHWNKSSSGKFVPIINPTTRKTHFKVQACTQKEVNRVMESAKTAQKSWAKTPLWKRAEL

LHKAAAILKEHKAPIAECLVKEIAKPAKDAVTEVIRSGDLVSYCAEEGVRILGEGKFLVSDSFPGNERTKYCLTSKIPLG

VVLAIPPFNYPVNLAVSKIAPALIAGNSIVLKPPTQGAVAALHMVHCFHLAGFPEGLISCVTGKGSEIGDFLTMHPGVNC

ISFTGGDTGIAISKKAGMVPLQMELGGKDACIVLEDADLDLAAANIVKGGFSYSGQRCTAVKVALVMESVANTLVKRIND

KIAKLTVGPPEIDSDVTPVVTESSANFIEGLVMDAKEKGATFCQEYVREGNLIWPLLLDNVRPDMRIAWEEPFGPVLPVI

RINSVEEGIHHCNASNFGLQGCVFTRDINKAMLISDAMETGTVQINSAPARGPDHFPFQGLKDSGIGSQGITNSINMMTK

VKTTIINLPAPSYTMG

>GmALDH12A1

MCLLRVLSAEFIFTICRFAHSLPFATVQAEEISDSRPAEVLNLVQGKWAGSSNWNTVVDPLNGDSFIKVAEVDETGIQPF

VESLSSCPKHGVHNPFKAPERYLMFGEISAKAAHMLSLPKVSDFFTRLIQRVSPKSYQQAFGEVYVTQKFLENFCGDQVR

FLARSFGVPGNHLGQQSHGFRWPYGPVAIITPFNFPLEIPVLQLMGALYMGNKPVLKVDSKVSIVMDQMLRLLHNCGLPL

EDVDFINSDGKTMNKLLLEANPRMTLFTGSSRVAEKLAVDLKGRVKLEDAGFDWKILGPDVLQEDYIAWVCDQDAYACSG

QKCSAQSLLFMHENWSKTSLLSKLKDLADRRKLADLTVGPVLTVTTDSMLEHINKLLEIPGSKLLFGGQPLEDHSIPPIY

GAMKPTAVYVPLEEIMKAKNFELVTREIFGPFQIVTDYKSSQLSVVLDALERMHNHLTAAVVSNDPLFLQEVIGQSVNGT

AYAGLRARTTGAPQNHWFGPAGDARGAGIGTPEAIKLVWSCHREIIYDFGPVPKNWEVPPST

>GmALDH12A2

MFMFLVSRVTKDSISRNRNAFASFAFSSRCAHSLSFATVEAEEISGSRPAEVLNLVQGKWVGSSNWNTIADPLNGDSFIK

VAEVDETGIQPFIKSLSSCPKHGVHNPFKAPERYLMYGDISTKAAHMLSLPKVSDFFTKLIQRVSPKSYQQAFGEVYVTQ

KFLENFCGDQVRFLARSFGVPGNHLGQQSHGFRWPYGPVAIITPFNFPLEIPVLQLMGALYMGNKPVLKVDSKVSIVMEQ

MLRLLHTCGLPAEDVDFINSDGKTMNRLLLEANPRMTLFTGSSRVADKLAVDLKGRVKLEDAGFDWKILGPDVHQEDYIA

WVCDQDAYACSGQKCSAQSLLFMHENWSKTSLLSKLKDLAERRKLEDLTIGPVLTCTTGMMLEHKNKLLEIPGSKLLFGG

SPLENHSIPPIYGAIKPTAVYVPLEEIMKDKNFDLVTKEIFGPFQVITDYKNSQLSVVLDAVERMHNHLTAAVVSNDPLF

LQEVVGNSVNGTTYAGLRARTTGAPQNHWFGPAGDARGAGIGTPEAIKLVWSCHREVIYDFGPVPKDWKTPQST

>GmALDH12A3

MFKLLVSRAARVSTPHNHNAFASFAFSRYAHSLPFATVEAEEISGSRAAEVLNLVQGKWVGSSNWNTVVDPLNGDSFIKV

AEVDETGIQPFVESLSSCPKHGAHNPFKAPESLLGVNGMVVLILFRYLMFGEISAKAAHMLSLPKVLDFFTRLIQRVSPK

SYQQAFGEVYVTQKFLENFCGDQVRFLARSFAVPGNHLGQQSHGFRWPYGPVAIITPFNFPLEIPVLQLMGALYMGNKPV

LKVDSKVSIVMEQMLRLLHTCGLPLEDVDFINSDGKTMNKLLLEGNPRMTLFTGSSRVAEKLAVDLKGRVKLEDAGFDWK

ILGPDVHQEDYVAWVCDQDAYACSGQKCSAQSLLFMHENWSKTSLLSKLKDLAERRKLADLTIGPVLTVTTDSMLEHVNK

LLEIPGSKLLFGGSPLENHSIPPIYGAIKPTAVYVPLEEIMKDKNFELVTKEIFGPFQVITDYQNSQLAVVLDALERMHN

HLTAAVVSNDPLFLQEVIGKSVNGTTYAGLRARTTGAPQNHWFGPAGDARGAGIGTPEAIKLVWSCHREIIYDFGPVPKN

WEVPPST

>GmALDH18B1

MELLQNGHKNFVSIKPSELPLTNGAALTLLNSLSKTQYLGNIDPSRVFVTKVKRIIVKVGTAVVTRSDGRLALGRIGALC

EQLKELSSQGYEVILVTSGAVGLGRQRLRYRKLANSSFSDLQKPQEELDGKACAAVGQSSLMALYDTMFSQLDVTSSQLL

VNDGFFRDSGFRKQLSDTVNSLLDLRVIPIFNENDAVSTRKAPYEDSSGIFWDNDSLAGLLALELKADLLVLLSDVEGLY

SGPPSDPNSRLIHTYIKEKHQGEITFGDKSRLGRGGMTAKVNAAVCAAHAGIPVIITSGYATNNIIRVLQGERIGTVFHK

DAHLWTNIKEVSAREMAVAAREGSRRLQILKSEERRKILLAIADALETSESMIRHENEADVADAVATGYEKSLMSRLILK

QEKISSLAKSVRMLADMEEPIGQILKRTELVDKLILEKISCPLGVLLVIFESRPDALVQIAALAIRSGNGLLLKGGKEAR

RSNAILHKVITSVMPDTVGDKLIGLVTSRDEILDLLKLDDVIDLVVPRGSNKLVSQIKESTKIPVLGHADGICHVYVDKS

ANIDMAKQIVRDAKTDYPAACNAMETLLVHKDLSNNGGLHELVLELQREGVKMFGGPRASGLLNIAETNTFHHEYSSLAC

TVEIVEDVFAAIDHINQHGSAHTECIVTEDSEVAETFLSQVDSAAVFHNASTRFCDGARFGLGAEVGISTSRIHARGPVG

VEGLLTNRWILRGSGHVVDGDQGINYTYKELPLKA

>GmALDH18B2

MADPSRSFMKDVKRVIIKVGTAVVTREEGRLAVGRLGALCEQIKQLNSLGYDIILVSSGAVGIGRQRLRYRKLINSSFAD

LQKPQHELDGKACAAVGQNSLMALYDTLFTQLDVTSAQLLVTDNDFRDKDFRKQLTETVKSLLSLKVIPVFNENDAVSTR

KAPYEDSSGIFWDNDSLSALLALELKADLLVLLSDVEGLYSGPPSDPHSKLIHTYIKEKHQNEITFGDKSRVGRGGMTAK

VKAAVHAADAGIPVVITSGFAAENIINVLQGQRIGTLFHKDAHEWVQVKEVDAREMAVAARECSRRLQAISSEERNQILH

KIADALEANEKIIRTENEADIAVAQEAGYEKSLVARLAIKPGKIASLANNMRIIANMEDPIGQVLKRTELSDGLILEKTS

SPLGVLLIVFESRPDALVQIASLAIRSGNGLLLKGGKEARRSNAILHKVITEAIPDTVGGKLIGLVTSREEIPELLKLDD

VIDLVIPRGSNKLVSQIKSSTKIPVLGHADGVCHVYVDKSANVEMARRIVLDAKIDYPAACNAMETLLVHKDLIEKGWLN

DIVVDLRTEGVKLYGGPRASSLLNIPQAQTFHHEYSSLACTVEIVDDVYAAIDHINLYGSAHTDSIVAEDKEVANVFLRQ

VDSAAVFHNASTRFSDGARFGLGAEVGISTSRIHARGPVGVEGLLTTRWILKGSGQVVDGDKGIVYTHKDIAT

>GmALDH18B3

MELLQNGHKNLVSIKPSELPLLNGAALTLLNSLSETHEYYGNIDPSRVFVTKVKRIIVKVGTAVVTRSDGRLALGRIGAL

CEQLKELSSQGYEVILVTSGAVGLGRQRLRYRKLANSSFSDLQKPQGELDGKACAAVGQSSLMALYDTMFSQLDVTSSQL

LVNDGFFRDSGFRKQLSDTVNSLLDLRVIPIFNENDAVSTRKAPYEDSSGIFWDNDSLAGLLALELKADLLVLLSDVEGL

YSGPPSDPNSKLIHTYVKEKHQGEITFGDKSRLGRGGMTAKVNAAVCAAHAGIPVIITSGYATNNIIRVLQGERIGTVFH

KDAHLWTNIKEMSAREMAVAAREGSRQLQILKSEDRRKILLAIADALEKNESMIRHENEADVADAVVAGYEKSLISRLTL

KQEKISSLAKSVRLLADMEEPIGQILKRTELVDKLILEKTSCPLGVLLVIFESRPDALVQIAALAIRSGNGLLLKGGKEA

RRSNAILHKVITSVMPDTVGDKLIGLVTSRDEIPDLLKLDDVIDLVVPRGSNKLVSQIKESTKIPVLGHADGICHVYVDK

SANFDMAKQIVRDAKTDYPAACNAMETLLIHKDLSNNGGLNELVLELQREGVKMFGGPRASGLLNIAETNTFHHEYSSLA

CTVEIVEDVFAAIDHINQHGSAHTECIVTEDSEVAETFLSQVDSAAVFHNASTRFCDGARFGLGAEVGISTSRIHARGPV

GVEGLLTNRWILRGSGHVVDGDQGIDYTYKELPLKA

>GmALDH18B4

MADRSRSFMKDVKRVVIKVGTAVVTREEGRLAVGRLGALCEQIKQLNSLGYDIILVSSGAVGIGRQRLRYRKLINSSFAD

LQKPQLELDGKACAAVGQNSLMALYDILFTQLDVTSAQLLVTDNDFRDEDFRKQLTETVKSLLSLKVIPVFNENDAVSTR

KAPYEDSSGIFWDNDSLSALLALELKADLLVLLSDVEGLYSGPPSDPHSKLIHTYIKEKHQNEITFGDKSRVGRGGMTAK

VKAAVHAADAGIPVVITSGFAAENIINVLQGQRIGTLFHKDAHEWVQVKEVDAREMAVAARECSRRLQAISSEERKQILL

KIADDLEANEKIIRTENEADVAVAQQAGYENSLVARLALKPGKIASLANNVRIIANMEDPIGQVLKRTELSDGLILEKTS

SPLGVLLIVFESRPDALVQIASLAIRSGNGLLLKGGKEAKRSNAILHKVITEAIPDTVGGKLIGLVTSREEIPELLKLDD

VIDLVIPRGSNKLVSQIKSSTKIPVLGHADGVCHVYVDKSANVEMARGIVLDAKLDYPAACNAMETLLIHKDLIEKGWLN

DIVVDLRTEGVKLYGGPRASSLLNIPQAHSFHHEYSSLACTVEIVDDVYAAIEHINLYGSAHTDSIIAEDKEVANVFLRQ

VDSAAVFHNASTRFSDGARFGLGAEVGISTSRIHARGPVGVEGLLTTRWILKGSGQVVDGDKGIVYTHKDLAA

>GmALDH18B5

MENTDPCRHFLKDVKRIIIKVGTAVVTRQDGRLAVGKLGALCEQIKELNSLGYEIILVSSGAVGLGRQRLRYRKLINSSF

ADLQKPQVELDGKACAAVGQNSLMALYDVLFSQLDVTSAQLLVTDNDFRDKDFRMQLSETMKSLLALKVIPIFNENDAVS

TRKAPYEDSSGIFWDNDSLSALLALELKADLLILLSDVEGLYSGPPSDPRSKLIHTYIKEKHQSEITFGDKSRVGRGGMT

AKVKASIHAAEAGIPVIITSGYAAENIIKVLQGQRIGTLFHKDAHKWAPVKEVDAREMAVAARDCSRRLQALSSEERKQI

LLKIADALEAHQNEIRIENEADVADAKEAGYEKSLVARLVLKNEKLASLANNIRIIANMEDPIGRVLKRTELAEGLILEK

TSSSLGVLLIVFESRPDALVQIASLAIRSGNGLLLKGGKEAKRSNAILHKVITEAIPDIVGSKLIGLVTSRAEIPELLKL

DDVIDLVIPRGSNKLVTQIKSSTKIPVLGHADGICHVYVDKSADLEMARRIVLDAKIDYPAGCNAMETLLVHKDLVEKGW

LNSIIIDLRTEGVTLYGGPKASPLLNIPMARMLHHEYNSLACTVEIVDDVYAAIDHINLYGSAHTDSVVAEDHEVANVFL

RQVDSAAVFHNASTRFSDGARFGLGAEVGISTSRIHARGPVGVDGLLTTRWILKGSGQIVDGDKAVNYTHRDLSI

>GmALDH22A1

MAFWWPLLVLAFAYGICRFLLMLIPPKVPSIDVDTSDVLDDGNQAQENSFIYVPPRGTSQQSGKIVQCYEPATMKYLGYV

PALTHEEVKDRVSKVRKAQKMWAKSSFKQRRLFLRILLKYIIKHQALICEISSRDTGKTMVDASLGEIMTTCEKINWLLS

EGEQWLKPEYRSSGRSMLHKRAKVEFHPLGVIGAIVSWNYPFHNIFNPMLAAIFSGNGIVIKISEHASWSGCFYFRIIQS

ALAAIGAPEDLVEVITGFAETGEALVSSVDKVIFVGSPGVGKMIMNNASNTLIPVTLELGGKDAFIVCEDVDLDHVAQIA

VRAVLQSSGQNCAGAERFYVHREIYSSFVSKVTKIVKSVTAGPPLVGKYDMGALCMHEHSEKLEGLVNDALDKGAEIVAR

GNLGHIGEDAVDQYFPPTVIVNVNHTMRLMQEEAFGPIMPIMKFSSDEEVVRLANDSKYGLGCAVFSGNQSRAREIASQI

HAGVAAVNDFASTYMCQSLPFGGVKHSGFGRFGGVEGLRACCLVKAVVEDRWWPFVKTKIPKPIQYPVAENGFEFQESLV

EALYGLGIWDRLRALVNVLKMLTEQNPGGSSNKRRND

>GmALDH22A2

MAFWWPLLVLALAFAICKFLLILIPPKVPSIDVDASDVLDDGSQAQENSFIYVPPRGTAQQSSGKVQCYEPATMKYLGYV

PALTPDEAREQVEKVRKAQKMWAKTSFKKRRQFLRILLKYIIKHQALICEISSRDTGKTMVDASLGEIMTTCEKINWLLS

EGEQCLKPEYRSSGRAMLHKRAKVEFHPLGVIGAIVSWNYPFHNIFNPMLAAVFSGNGVVIKISEHASWSGCFYFRIIQS

ALAAIGAPEDLVEVITGFAETGEALVSSADKVIFVGSPGVGKMIMSNAAETLIPVTLELGGKDAFIVCEDVDVDLVAQIA

VRAALQSSGQNCAGAERFYVHRKIYASFGPPLAGKYDMGALCMHAHSEMLEALINDALDKGAEIIARGSFGPIGEDAVDQ

YFPPTVIVNVNHSMRLMQEEAFGPIMPIMKFSSDEEVVRLANDSKYGLGCNVFSGSQSRAREIASQIHCGLAAVNDFAST

YMCQSLPFGGVKNSGFGRFGGVEGLRACCLVKSVVEDRWWPFIKTVIPKPIQYPVAENGFEFQESLVEALYGLSVWDRLQ

ALVNVLKMLTEQNSTSGSRKKKND

>GmALDH22A3

MAFWWPLLVLALAFAICKFLLILIPPKVPSIDVDASDVLDDGSLTQENSFIYVPPRGTAQQSSGKVQCYEPATMKYLGYV

PALTPDEVKEQVEKVRKAQKMWAKTSFKKRRHFLRILLKYIIKHQALICEISSRDTGKTMVDASLGEIMTTCEKINWLLS

EGEQCLKPEYRSSGRAMLHKRSKVEFLPLGVIGAIVSWNYPFHNIFNPMLAAVFSGNGIVIKISEHASWSGCFYFRIIQS

ALAAIGAPEELVEVITGFAETGEALVASADKVIFVGSPGVGKMIMSNAAETLIPVTLELGGKDVFIVCEDADVDHVAQVA

VRAALQSSGQNCAGAERFYVHRNIYASFVSKVTKIIKSVTAGPPLAGKYDMGALCMHAHSEKLEALINDALDKGAEIIAR

GSFGHIGEDAVDQYFPPTVIVNVNHSMRLMQEEAFGPIMPIMKFSSDEEVVRLANDSKYGLGCNVFSGSQSRAREIASQI

HCGLAAVNDFAATYMCQSLPFGGVKNSGFGRFGGVEGLRACCLVKSVVEDRWWPFIKTVIPKPIQYPVAENGFEFQESLV

EALYGLSVWDRLQALVNVLKMLTEQNSTSGSRKKKND

>GmALDH22A4

MAFWWPLLVLAFAYGICRFLLMLIPPKVPSIDVDTSDVLDDGNQAQENSFIYVPPRGTSQQSGKIVQCYEPATMKYLGYV

PALTRDEVKDRVAKVRKAQKMWAKSSFKQRRLFLRILLKYIIKHQALICEISSRDTGKTMVDASLGEIMTTCEKINWLLS

EGEQWLKPEYRSSGRSMLHKRAKVEFHPLGVIGAIVSWNYPFHNIFNPMLAAIFSGNGIVIKISEHASWSGCFYFRIIQS

ALAAIGAPEDLVEVITGFAETGEALVSSVDKVIFVGSPGVGKMIMNNAANTLTPVTLELGGKDAFIVCEDVDLDHVAQIA

VRAVLQSSGQNCAGAERFYVHREIYSSFVSLVTKIVKSVTAGPPLVGKYDMGALCMHEHSEKLEGLVNDALDKGAEIVAR

GSFGHIGEDAVDQYFPPTVIVNVNHTMRLMQEEAFGPIMPIMKFSSDEEVVRLANESKYGLGCAVFSGNQSRAREIASQI

HAGVAAVNDFASTYMCQSLPFGGVKHSGFGRFGGVEGLRACCLVKAVAEDRWWPFVKTKIPKPIQYPVAENGFEFQESLV

EALYGIGIWDRLRALVNVLKMLTEQHPGGGGKRRND

>OsALDH2B1 Os06g0270900 [Oryza sativa Japonica Group]

MAAAAARRGSSLLSRCLLSRPAAAASPAVPSALRRADGTQGLLPGILQRFSTAAVAEEPISPPVQVNYTQLLIDGKFVDS

ASGKTFPTLDPRTGELIAHVAEGDAEDINRAVHAARKAFDEGPWPKMTAYERSRILLRFADLIEKHNDEIAALETWDNGK

PYAQAANIEVPMVARLMRYYAGWADKIHGLVVPADGPHHVQVLHEPIGVAGQIIPWNFPLLMFAWKVGPALACGNTVVLK

TAEQTPLSALFASKLLHEAGLPDGVVNVVSGFGPTAGAALASHMDVDKIAFTGSTDTGKVVLELAARSNLKSVTLELGGK

SPFIIMDDADVDHAVELAHFALFFNQGQCCCAGSRTFVHERIYDEFVEKAKARALKRVVGDPFKNGVEQGPQIDDEQFNK

ILRYIKYGVDSGANLVTGGDRLGDKGYYIQPTIFSDVQDNMRIAQEEIFGPVQSILKFNDLNEVIKRANASQYGLAAGVF

TNNLNTANTLTRALRVGTVWVNCFDVFDAAIPFGGYKQSGIGREKGIDSLKNYLQVKAVVTPIKNAAWL

>OsALDH2B5 Os02g0730000 [Oryza sativa Japonica Group]

MAARRAASSLLSRGLIARPSAASSTGDSAILGAGSARGFLPGSLHRFSAAPAAAATAAATEEPIQPPVDVKYTKLLINGN

FVDAASGKTFATVDPRTGDVIARVAEGDAEDVNRAVAAARRAFDEGPWPRMTAYERCRVLLRFADLIEQHADEIAALETW

DGGKTLEQTTGTEVPMVARYMRYYGGWADKIHGLVVPADGPHHVQVLHEPIGVAGQIIPWNFPLLMFAWKVGPALACGNA

VVLKTAEQTPLSALFVASLLHEAGLPDGVLNVVSGFGPTAGAALSSHMGVDKLAFTGSTGTGKIVLELAARSNLKPVTLE

LGGKSPFIVMDDADVDQAVELAHRALFFNQGQCCCAGSRTFVHERVYDEFVEKARARALQRVVGDPFRTGVEQGPQIDGE

QFKKILQYVKSGVDSGATLVAGGDRAGSRGFYIQPTVFADVEDEMKIAQEEIFGPVQSILKFSTVEEVVRRANATPYGLA

AGVFTQRLDAANTLARALRVGTVWVNTYDVFDAAVPFGGYKMSGVGREKGVYSLRNYLQTKAVVTPIKDAAWL

>OsALDH2C1 Os01g0591000 [Oryza sativa Japonica Group]

MAAANGGDSKGFEVPKLEIKFTKLFINGRFVDAVSGKTFETRDPRTGEVIAKIAEGDKADIDLAVKAAREAFDHGPWPRM

SGFARGRILHKFADLVEQHVEELAALDTVDAGKLFAMGKLVDIPGGANLLRYYAGAADKVHGETLKMARPCHGYTLKEPV

GVVGHIVPWNYPTTMFFFKASPALAAGCTMVVKPAEQTPLSALFYAHLAKLAGVPDGVLNVVPGFGPTAGAAISSHMDID

KVSFTGSTEVGRLVMEAAAKSNLKPVSLELGGKSPVIVFDDADLDTAVNLVHMASYTNKGEICVAGSRIYVQEGIYDAFV

KKATEMAKKSVVGDPFNPRVHQGPQIDKEQYEKILKYIDIGKREGATLVTGGKPCGENGYYIEPTIFTDVKEEMSIAQEE

IFGPVMALMKFKTVEEAIQKANSTRYGLAAGIVTKNIDVANTVSRSIRAGAIWINCYLGFDPDVPFGGYKMSGFGKDMGM

DALEKYLHTKAVVTPLYNTPWL

>OsALDH2C2 Os01g0591300 [Oryza sativa Japonica Group]

MGSTGDCGNGKAAAGGGGLVVPEIKFTKLFINGEFVDAASGKTFKTRDPRTGDVLAHIAEADKADVDLAVKAAREAFEHG

KWPRMSGYERSRVMNKLADLVEQHADELAALDGADAGKLLTLGKIIDMPAAAQMMRYYAGAADKIHGESLRVAGKYQGYT

LREPIGVVGVIIPWNFPTMMFFLKVSPALAAGCTIVVKPAEQTPLSALYYAHLAKLAGVPDGVINVVPGFGPTAGAALSS

HMDVDSVAFTGSAEIGRAIMESAARSNLKNVSLELGGKSPMIVFDDADVDMAVSLSSLAVFFNKGEICVAGSRVYVQEGI

YDEFVKKAVEAAKNWKVGDPFDAATNMGPQVDKVQFERVLKYIEIGKNEGATLLTGGKPTGDKGYYIEPTIFVDVKEEMT

IAQEEIFGPVMSLMKFKTVEEAIEKANCTKYGLAAGIVTKNLNIANMVSRSVRAGTVWVNCYFAFDPDAPFGGYKMSGFG

RDQGMVAMDKYLQVKTVITAVPDSPWY

>OsALDH2C3 putative cytosolic aldehyde dehydrogenase [Oryza sativa Japonica Group]

MASESESNGHHHAGDEARAENSGAGCLHGGDDGGGTPRTTPEIRYTKLFINGRFVDAASGKTFETRDPRTGDVIARVAEG

DKEDVDLAVKAAREAFDHGEWPRMSGSERGRVMAKYAEVVERHADELAALESLDAGKPLAAARAVDVGECVGILRYFAGA

ADKIHGETLKMSRQLQGYTLREPLGVAGLIVPWNFPAIMFFSKVSPALAAGCTVVVKPAEQTPLSALFLAHLSKQAGVPD

GVINVVTGFGPTAGAAISSHMDVDVVAFTGSTEVGRLIMEASAKSNLKPVALELGGKSPFIVFDDADLDKAVELAIGGNF

FNKGEACVAGSRVFVQEGIYDRFEQKLADTMKSWVVGDPFDPRVNQGPQVDKAQYERVLGYIEQGKAEGATVLTGGKPCG

KKGYYIEPTIFTNVKDDMVIAREEIFGPVMCLMKFKTVEEAIERANGTRYGLAAGLVTRDIDVANRMARSIRAGVVWVNC

YFAMDRSCPFGGRKMSGFGKDDSMHALDKFLAVKSVVTPVHGSPWF

>OsALDH3E1 Os02g0646500 [Oryza sativa Japonica Group]

MEEKPQHGSLGGLVAGVREEYESGRTKELEWRKAQLGGLIRMITEEEDAIFDALHDDLGKHRVESFRDEVGVLAKSVRNT

LQNLKKWASPEKVDVPLISFPCNARVVPEPIGVVLIFSCWNLPIGLALEPLSGAIAAGNAVVLKPSEFAPSTAAFLAANI

PKYLDANAVKVVQGGAEVGEELMEHRWDKVLFTGNARVGRIIMTKAAKHLTPVALELGSKCPCIVDCLDSKRECQVAVNR

IIGAKWSTCAGQACVAIDYILVEEQFAPFLIELLKSTLKRFFTEPEYMARILNEKHFHRLTNLLEDDQVKSSIVHGGNAD

PKTLWIEPTIVLNPPFDSDIMMEEIFGPLLPIITVKKTEDCIAFLKSKPKPLAIYAFTNNEKLKQRIVAETSSGSVLFND

AIVQYGLDSVPFGGIGESGFGQYHGKYTFELFSHRKAVVRRSLLVEFMFRYPPWDEYKMGMLRRVFRFDYVSLVLALLAF

WLLGIRR

>OsALDH3E2 Os04g0540600 [Oryza sativa Japonica Group]

MGRVAPSVEEVGGEQPPPALGPGETVSGTVAELRAAYESGRTRSLEWRQSQLRGLLRLLAEEEAAAFRALREDLGKHQAE

AYRDEIGVLVKSANAALREVGKWMAPEKVWVPLIAFPARAQLEPQPLGVILVFSCWNVPLGLSLEPLVGALAAGNAVALK

PSELAPATAKFLGDNVGKYMDATAVKVIQGGPEVGEQLMEHRWDKVLFTGSPRIARVVMAAAAKHLTPVALELGGKCPCI

FDTIGGSARDLQTAVNRVVGGKWSSCAGQACLAIDYVLVEERFVPVLIKALKSTLKKFFADSDHMARIVNARHFQRLSDL

LKDKSVAASVLHGGTLDAKNLCIEPTILLNPPLDSAIMTEEIFGPLLPIITVKKIEDSIAFVRARPRPLAVYAFTKNAAL

RRRIVEETSSGSVTFNDAVVQYGIDSLPFGGVGESGFGQYHGKYSFEMFSHKKAVLTRGYLIELTARYPPWDDSKISMMR

QLYRYNYVGFVLTFLGLKK

>OsALDH3E3 Os02g0647900 [Oryza sativa Japonica Group]

MAPAMVAAMGEKPKPAVVLGGMVSGLREVYESGRTKDLEWRQSQLKALIRLLTDKEEEIFAVLHDDLGKHRGESFRDELG

ILVKSIKYTLQNLKKWAASERAESPLVAFPATAMVVPEPLGVVLVFSCWNLPLGLALEPLSGAIAAGNAVVLKPSELAPS

TAAFLAANIPRYLDSRAVKVVLGGPNVGEELMEHRWDKVLFTGSARIGRIIMAKAVKHLTPVALELGSKCPCIVDWLDSK

RDRQIAVNRIIGAKWSTCAGQACIAIDHVIVEERFAPILIELLKSTLKRFMAKPGGMARILNAKHFERLSGYLEDNRVAA

SVVHGGYMDPKKLNIEPTLLLNPPADSDVMTEEVFGPILPIITVKKIEDCIAYLKSKPKPIAMYAFTNNERLKRRIVEET

SSGSVTFNDAVVQYALESVPFGGVGHSGFGQYHGKYSFELFSHKKAVFKRSFLIEFMFRYPPWDERKIGTLRHVFSYNYF

LLFFNLLGFRR

>OsALDH3H1 aldehyde dehydrogenase family protein, expressed [Oryza sativa Japonica Group]

MAAARSVGMEAEVAALRGRFAAGGTRGAEWRAAQLRGILRMAAEAEAEVCRALHADLAKPYTESYVHEIALVKSSCKFAL

KNLKKWMKPQKVTAPLMTFPSTARVAAEPLGVVLVISAWNYPFLLSIDPIIGAIAAGNAVVLKPSEVAPATSSLLAELLP

RYVDGSCIKVVEGGVAETTTLLEQKWDKIFYTGNGKVGRIVMASAAKHLTPVVLELGGKCPVVVDSNVNLHVTAKRIAAG

KWGCNNGQACISPDFIITTKSFAPKLLEALEKVLEKFYGRDPLRSSDLSRIVNSNHFNRLKKLMDDENVSDKIVFGGQRD

EHQLKIAPTIFMDVPLDSGIMKEEIFGPLLPIITVDKIHESFALINSMTKALAAYLFTKDSKLQEQYEAAISAGGMLVND

TAVHLTNQYLPFGGVGESGMGAYHGRFSFEAFSHKKAVLVRRFAGEAAARYPPYSPAKLKILRGVLKGNLGAMIKAILGF

PRGK

>OsALDH3H2 Os11g0186200 [Oryza sativa Japonica Group]

MAEEEVAAVVGELRGSFRSGRTRAAEWRAAQLRGIVRMVEEREGDISDALHSDLAKPRMESYLHEISLAKAACTFALKGL

KNWMKPEKVPAALTTFPSTAQIVSEPLGVVLVISAWNYPFLLSIDPVIGAIAAGNAVVLKPSEIAPATSALFAKLLPEYV

DSSCIKVVEGGVPETTALLEQKWDKIFYTGSGNVGRIVMAAAAKHLTPVALELGGKCPAIVDSNTDLHVTMKRLAVGKWG

CNNGQACIAPDYVITTKSFAPELVDSLKRVLKRFYGEDPLQSEDLSRIVNSNHFRRLTNLIEDKKVAQKIVYGGQTDEKQ

LKIAPTVLLDVPLDTTLMAEEIFGPLLPIVTVDKIEDSIQFINSRTKPLAAYLFTKDKKLQEEFVSNVPAGGMLVNDVAL

HLANPHLPFGGVGDSGIGSYHGKFSFDCFTHKKAVLIRGFGGEATARYPPYTIEKQKILRGLINGSFFALILALLGFPKE

RR

>OsALDH5F1 RecName: Full=Succinate-semialdehyde dehydrogenase, mitochondrial; AltName: Full=Aldehyde dehydrogenase family 5 member F1; AltName: Full=NAD(+)-dependent succinic semialdehyde dehydrogenase; Flags: Precursor

MAMAMAMRRAAALGARHILAASSTSSSGVLLRRHMSVDAGAAMEKVRAAGLLRTQGLIGGKWVDAYDGKTIEVQNPATGE

TLANVSCMGSKETSDAIASAHSTFYSWSKLTANERSKALRKWHDLIISHKEELALLMTLEQGKPMKEALVEVTYGASFIE

YFAEEAKRIYGDIIPPTLSDRRLLVLKQPVGVVGAVTPWNFPLAMITRKVGPALACGCTVVVKPSEFTPLTALAAADLAL

QAGIPAGAINVVMGNAPEIGDALLQSTQVRKITFTGSTAVGKKLMAGSANTVKKVSLELGGNAPCIVFDDADIDVAIKGS

LAAKFRNSGQTCVCANRILVQEGIYEKFASAFIKAVQSLKVGNGLEESTSQGPLINEAAVQKVEKFINDATSKGANIMLG

GKRHSLGMSFYEPTVVGNVSNDMLLFREEVFGPVAPLVPFKTEEDAIRMANDTNAGLAAYIFTKSIPRSWRVSEALEYGL

VGVNEGIISTEVAPFGGVKQSGLGREGSKYGMDEYLELKYICMGNLN

>OsALDH6B1 Os07g0188800 [Oryza sativa Japonica Group]

MLRAALLRSGSGLRRPPMAAPLSTAAAASWLSDSASSPPRVRLLIGGEFVESRADEHVDVTNPATQEVVSRIPLTTADEF

RAAVDAARTAFPGWRNTPVTTRQRIMLKYQELIRANMDKLAENITTEQGKTLKDAWGDVFRGLEVVEHACGMGTLQMGEY

VSNVSNGIDTFSIREPLGVCAGICPFNFPAMIPLWMFPIAVTCGNTFVLKPSEKDPGAAMMLAELAMEAGLPKGVLNIVH

GTHDVVNNICDDEDIKAVSFVGSNIAGMHIYSRASAKGKRVQSNMGAKNHAIILPDADRDATLNALIAAGFGAAGQRCMA

LSTAVFVGGSEPWEDELVKRASSLVVNSGMASDADLGPVISKQAKERICKLIQSGADNGARVLLDGRDIVVPNFENGNFV

GPTLLADVKSEMECYKEEIFGPVLLLMKAESLDDAIQIVNRNKYGNGASIFTTSGVSARKFQTDIEAGQVGINVPIPVPL

PFFSFTGSKASFAGDLNFYGKAGVQFFTQIKTVTQQWKESPAQRVSLSMPTSQK

>OsALDH7B6 Os09g0440300 [Oryza sativa Japonica Group]

MGSFARKEHQFLAELGLAPRNPGSFACGAWGGSGPVVTSTNPTNNQVIAEVVEASAREYEEGMRACYDAAKTWMAIPAPK

RGEIVRQIGDALRAKLHHLGRLVSLEMGKILPEGIGEVQEIIDMCDYAVGLSRQLNGSIIPSERPNHMMMEVWNPLGVVG

VITAFNFPCAVLGWNACIALVCGNCVVWKGAPTTPLITIAMTKIVASVLERNNLPGSIFTAFCGGADIGQAISLDTRIPL

VSFTGSTKVGLMVQQQVNARFGKCLLELSGNNAIIVMDDADIQLAVRSVLFAAVGTAGQRCTTCRRLLLHESIYRTFLDQ

LVEVYKQVRIGDPLENGTLLGPLHTPASRDAFLKGIQTIRSQGGKILYGGSAIESEGNFVQPTIVEISPSAPVVREELFG

PVLYVMKVQNLKEAVEINNSVPQGLSSSIFTKRPDIIFKWIGPHGSDCGIVNVNIPTNGAEIGGAFGGEKATGGGREAGS

DSWKQYMRRATCTINYGSELPLAQGINFG

>OsALDH10A5 Os04g0464200 [Oryza sativa Japonica Group]

MAAPSAIPRRGLFIGGGWREPSLGRRLPVVNPATEATIGDIPAATAEDVELAVSAARDAFGRDGGRHWSRAPGAVRAKYL

KAIAAKIKDKKSYLALLETLDSGKPLDEAAGDMEDVAACFEYYADLAEALDGKQRAPISLPMENFESYVLKEPIGVVGLI

TPWNYPLLMATWKVAPALAAGCTAVLKPSELASLTCLELGGICAEIGLPPGVLNIITGLGTEAGAPLASHPHVDKIAFTG

STETGKRIMITASQMVKPVSLELGGKSPLIVFDDVDIDKAVEWAMFGCFANAGQVCSATSRLLLHEKIAKRFLDRLVAWA

KSIKISDPLEEGCRLGSVVSEGQYQKIMKFISTARCEGATILYGGARPQHLKRGFFIEPTIITNVSTSMQIWREEVFGPV

ICVKEFRTEREAVELANDTHYGLAGAVISNDLERCERISKAIQSGIVWINCSQPCFVQAPWGGNKRSGFGRELGQWGLDN

YLSVKQVTKYCSDEPYGWYRPPSKL

>OsALDH10A9 Os08g0424500 [Oryza sativa Japonica Group]

MATAIPQRQLFVAGEWRAPALGRRLPVVNPATESPIGEIPAGTAEDVDAAVAAAREALKRNRGRDWARAPGAVRAKYLRA

IAAKIIERKSELARLETLDCGKPLDEAAWDMDDVAGCFEYFADLAESLDKRQNAPVSLPMENFKCYLRKEPIGVVGLITP

WNYPLLMATWKVAPALAAGCTAVLKPSELASVTCLELADVCKEVGLPSGVLNIVTGLGSEAGAPLSSHPGVDKVAFTGSY

ETGKKIMASAAPMVKPVSLELGGKSPIVVFDDVDVEKAVEWTLFGCFWTNGQICSATSRLILHKKIAKEFQERMVAWAKN

IKVSDPLEEGCRLGPVVSEGQYEKIKQFVSTAKSQGATILTGGVRPKHLEKGFYIEPTIITDVDTSMQIWREEVFGPVLC

VKEFSTEEEAIELANDTHYGLAGAVLSGDRERCQRLTEEIDAGIIWVNCSQPCFCQAPWGGNKRSGFGRELGEGGIDNYL

SVKQVTEYASDEPWGWYKSPSKL

>OsALDH11A3 Os08g0440800 [Oryza sativa Japonica Group]

MAAVAGTGVFAEILEGEVYRYYADGEWRVSASGKSVAIVNPTTRLTQYRVQACTQEEVNKVMETAKVAQKAWARTPLWKR

AELLHKAAAILKEHKTPIAECLVKEIAKPAKDAISEVVRSGDLVSYTAEEGVRILGEGKLLVSDSFPGNERNKYCLSSKV

PLGVVLAIPPFNYPVNLAVSKIGPALIAGNALVLKPPTQGAVAALHMVHCFHLAGFPKGLINCVTGKGSEIGDFLTMHPG

VNCISFTGGDTGIAISKKAGMVPLQMELGGKDACVVLEDADLDLVAANIVKGGFSYSGQRCTAVKVVLIMESVADIVVEK

VKAKLAKLTVGPPEADSDITPVVTESSANFIEGLVMDAKEKGATFCQEYRREGNLIWPLLLDHVRPDMRIAWEEPFGPVL

PVIRINSVEEGIHHCNASNFGLQGCVFTKDINKAIMISDAMETGTVQINSAPARGPDHFPFQGLKDSGIGSQGITNSINM

MTKVKSTVINLPSPSYTMG

>OsALDH12A1 hypothetical protein OsJ_19352 [Oryza sativa Japonica Group]

MSLILSRRRLAAAVRRSGPAALASRWMHTPPFATVSPQEISGSSPAEVQNFVQGSWTTSGNWNWLVDPLNGEKFIKVAEV

QEAEIKPFVESLSNCPKHGLHNPLKAPERYLMYGDISAKAANMLGQPVVSDFFAKLIQRVSPKSYQQALAEVQVSQKFLE

NFCGDQVRFLARSFAVPGNHLGQSSNGYRWPYGPVAIITPFNFPLEIPLLQLMGALYMGNKPVLKVDSKVSIVMDQMLRL

LHACGMPAEDVDFINSDGITMNKLLLEANPKMTLFTGSSRIAEKLAADLKGKIKLEDAGFDWKILGPDVQEVDYIAWVCD

QDAYACSGQKCSAQSILFMHKNWSSSGLLDKMKSLSERRKLEDLTIGPVLTVTTSSMIEHMKNLLKIPGSKVLFGGEPLE

NHSIPEIYGAFKPTAVFVPLSEILKSGNFELVTREIFGPFQVVTEYSDDELELVLEACERMNAHLTAAVVSNDPLFLQEV

LGRSVNGTTYAGIRARTTGAPQNHWFGPAGDPRGAGIGTPEAIKLVWSCHREIIYDIGPLPKNRALPSAT

>OsALDH18B1 Os05g0455500 [Oryza sativa Japonica Group]

MASVDPSRSFVRDVKRVIIKVGTAVVSRQDGRLALGRVGALCEQVKELNSLGYEVILVTSGAVGVGRQRLRYRKLVNSSF

ADLQKPQMELDGKACAAVGQSGLMALYDMLFNQLDVSSSQLLVTDSDFENPKFREQLTETVESLLDLKVIPIFNENDAIS

TRKAPYEDSSGIFWDNDSLAGLLALELKADLLILLSDVDGLYSGPPSEPSSKIIHTYIKEKHQQEITFGDKSRVGRGGMT

AKVKAAVLASNSGTPVVITSGFENRSILKVLHGEKIGTLFHKNANLWESSKDVSTREMAVAARDCSRHLQNLSSEERKKI

LLDVADALEANEDLIRSENEADVAAAQVAGYEKPLVARLTIKPGKIASLAKSIRTLANMEDPINQILKKTEVADDLVLEK

TSCPLGVLLIVFESRPDALVQIASLAIRSGNGLLLKGGKEAIRSNTILHKVITDAIPRNVGEKLIGLVTTRDEIADLLKL

DDVIDLVIPRGSNKLVSQIKASTKIPVLGHADGICHVYIDKSADMDMAKHIVMDAKIDYPAACNAMETLLVHKDLMKSPG

LDDILVALKTEGVNIYGGPIAHKALGFPKAVSFHHEYSSMACTVEFVDDVQSAIDHIHRYGSAHTDCIVTTDDKVAETFL

RRVDSAAVFHNASTRFSDGARFGLGAEVGISTGRIHARGPVGVEGLLTTRWILRGRGQVVNGDKDVVYTHKSLPLQ

>OsALDH18B2 Os01g0848200 [Oryza sativa Japonica Group]

MGRGGIGGAGLVAAVAKADVENTDSTRGFVKDVKRIIIKVGTAVVTGPNGRLAMGRLGALCEQVKQLNFEGYEVILVTSG

AVGVGRQRLKYRKLVNSSFADLQNPQMDMDGKACAAVGQSVLMAIYDTLFSQLDVTSSQLLVTDRDFMDPSFGNQLRETV

NSLLDLKVIPVFNENDAISTRRQPYEDSSGIFWDNDSLARLLAQELKADLLIMLSDVEGLYSGPPSDPQSKIIHTYVHEQ

HGKLISFGEKSRVGRGGMQAKVAAAFTASSKGIPVVIASGFAIDSIIKVMRGEKIGTLFHREANQWGCSKEATAREMAVA

ARDCSRHLQKLSSEERKKILLDIADALEANEDLITSENQADLDLAQDIGYDKSLVARMTIKPGKIKSLAGSIREIADMED

PISHTLKRTEVAKDLVFEKTYCPLGVLLIIFESRPDALVQIASLAIRSGNGLLLKGGKEAMRSNTILHKVITGAIPDVVG

KKLIGLVKNKDEIADLLKLDDVIDLVIPRGSNKLVSQIKAATKIPVLGHADGICHVYIDKSADMDMAKRIVLDAKVDYPA

ACNAMETLLVHKDLNRTEGLDDLLVELEKEGVVIYGGPVAHDTLKLPKVDSFHHEYNSMACTLEFVDDVQSAIDHINRYG

SAHTDCIITTDGKAAETFLQQVDSAAVFHNASTRFCDGARFGLGAEVGISTGRIHARGPVGVDGLLTTRCILRGSGQVVN

GDKGVVYTHRELPLQ

>OsALDH22A1 Os07g0688800 [Oryza sativa Japonica Group]

MALWWPLLVLAAAYALCRILLFLIPPTVPSIDVDASDVLEDANQNKEDSYIYIPPRKGKGAQTDKVQCYEPATMKYLGYF

PALTPDEVKEHVAQARKAQKIWAKSSFKQRRQFLRILLKYILEHQDLICEISSRDTGKTMVDASLGEIMTTCEKITWLLD

EGEKWLKPEYRSCGRSMLHKKAKVEFYPLGVIGAIVSWNYPFHNVFNPMLAAIFSGNAAVIKVSEHASWSGCFYFRIIQA

ALAAVGAPDNLVHIITGFAETGQALVSSVDKIIFVGSPGVGRMIMNRASDTLIPVTLELGGKDAFIVCEDVDLPSVVQVA

VRAALQSSGQNCAGAERFYVHKDIYSTFVSQVVKIIKSISVGPPLSGRYDMGAICMIEHSEKLQNLVNDAVDKGAEIAGR

GSFGHLGEDAVDQFFPPTVLVNVNHTMKIMQEEAFGPILPIMKFNSDEEVVKLANDSKYGLGCAVFSGNQKRAIKIASQL

HCGVAAINDFASSYMCQSLPFGGVKDSGFGRFAGVEGLRACCLVKAVVEDRWWPYVKTMIPKPIQYPVSENGFEFQELLV

ETLYGLSVWDRLRSLVNLLKMISEQNNSPANTRKKSR

>SlALDH2B1

MDSLLMQPQTFPTFDPRTGEAITTVAEADTEDVNRAVFAARKAFDEGPWPKMTCAERSCIMLQFADLLERHSDELAALET

WDKGKPYEQAANEEIPMLIRLFRYYAGWADKIHGLTAPADSLHHVQTLHEPIGVAGQIIPWNFPLLMFAWKVGPALACGN

TVVLKPAEQTPLSALYVSKLFHEVGLPPGVLNVIPGSGSAGADLASHMDVDKIAFTGSTETGKAVVGAAAKSNLKPVTLE

LGGKSPFIICEDADVDKAVELAHSAVFFNQGQCCCAGSRTFVHERVYDEFVEKAKARALKRIVGDPFKKGVEQGPQIDTE

QFEKILKYIKSGTESGATLESGGEKLGSKGFYVQPTVFSNVQDNMLIARDEIFGPVQSLLKFKDVEEVIRRANSSHYGLA

AGVFTQNIDAANTISRALRVGTVWVNCFNIFDAAIPFGGYKMSGHGREKGVYSLSNYLQVKAIVTPLKNPAWL

>SlALDH2B3

MAARVFLSRSVHLLSKGKRSHLGKIAAYKYSTAAAFEEPVKPTVNVDHTKLLINGQFVDSASGKTFPTLDPRTGEVIAHI

AEGDAEDINRAVAAARKAFDEGPWPRMTAYERSKILLRLADLIEKHNDQIATLETWDTGKPYAQAAKIEVPMVVRLLRYY

AGWADKIHGMTIPADGPYHVQTLHEPIGVAGQIIPWNFPLLMFSWKIGPALACGNTIVLKTAEQTPLSALYVASLFQEAG

LPEGVLNIISGYGATAGASLCSHMDVDKLAFTGSTETGKTILELAAKSNLKPVTLELGGKSPFIVCEDADIDTAVEQAHF

ALFFNQGQCCCAGSRTYVHEKVYDEFLEKAKARALKRVVGDPFKSGTEQGPQIDSKQFDKIMKYIRSGVDSGATLETGGE

QFGKKGYYIRPTVFSNVKDDMLIAQDEIFGPVQSILKFKDLDEVVRRANSSRYGLAAGVFSQNIDTANTLARALRVGTVW

INCFDTFDATIPFGGYKMSGQGREKGEYGLKNYLQVKAVVTPLKNPAWL

>SlALDH2B4

MAFRLITSRLSHSSSSSLASLFQGRNSRVAATAALRYTTAAQDPIKPSVNVEYTKLFINGQFVDSASGKTFPTLDPRTGE

VIAHVAEGDVEDINRAVVAARNAFDEGPWPKMSAYERSKILFRIADLIEKHNDEIATLETWDSGKLYQQVATIEIPMIVR

LLRYYAGWADKIHGMTVPADGPYHVQTLHEPIGVVGQIIPWNFPLLMFAWKIGPALACGNTVVLKTAEQTPLSALYVSKL

LQEAGLPEGVLNVISGFGPTAGAALSSHMDVDKLAFTGSTDTGKTIMSLAANSNLKPVTLELGGKSPFIVCEDADVDQAV

EFAHFALFFNQGQCCCAGSRTYVHESIYDEFVEKAKARALKRTVGDPFDSSNEQGPQISSEQFEKVLKYIRSGIESGATL

ETGGDRLGTQGYYIKPTVFSNVKDDMLIATDEIFGPVQSILKFKDHDEVIRRANATKYGLAAGVFTKNIDTANTFMRALR

VGTIWINCFDIFDAAIPFGGYKMSGQGREKGEYSLKQYLQVKAVVTSLKNPAWL

>SlALDH2B7a

MAARRISSLLSRSLNLPSVSASLGRSHGVARHINRFSTAAAVEEIITPPVQINHTKLLINGQFVDSASGKTFPTLDPRTG

EVIANVAEGDLEDVNRAVAAARKAFDEGPWPKMSAYERSRIMLKFADLVEKHNDEIAALETWDNGKPYLQAAQAEVPSFV

RLFRYYAGWADKIHGLTVPADGPHHVQILHEPIGVAGQIIPWNFPLLMMAWKVGPALACGNTIVLKTAEQTPLTALYVAN

LLHEAGLPPGVLNIVSGFGPTAGAALASHMDVDKLAFTGSTETGQTVLQLAAKSNLKPVTLELGGKSPFIICEDADIDHA

VELAHFALFFNQGQCCCAGSRTYVHERVYDEFVEKAKARAMRRVVGDPFKKGVEQGPQIDSEQFQKILRYIREGRDSSAT

LECGGDRIGSKGYFIQPTVFSNVKEDMSIAQDEIFGPVQCVFKFKDIGEVIKRANNTRYGLAAGVFTKNIDTANTLTRGL

RAGTVWVNCYDIFDAGIPFGGYKMSGMGREKGIYSLNNYLQVKAVVTPLKNPAWI

>SlALDH2B7b

MNTHTRRIISQLRTSIRYSNYWRKGIRRFCSSAVVHEEPITPPVEVKYNQLLINGQFVDAASGKTFPTFDPRTGEAITTV

AEADTEDVNRAVFAARKAFDEGPWPKMTCAERSCIMLQFADLLERHSDELAALETWDKGKPYEQAANEEIPMLIRLFRYY

AGWADKIHGLTAPADSLHHVQTLHEPIGVAGQIIPWNFPLLMFAWKVGPALACGNTVVLKPAEQTPLSALYVSKLFHEVG

LPPGVLNVIPGSGSAGADLASHMDVDKIAFTGSTETGKAVVGAAAKSNLKPVTLELGGKSPFIICEDADVDKAVELAHSA

VFFNQGQCCCAGSRTFVHERVYDEFVEKAKARALKRIVGDPFKKGVEQGPQIDTEQFEKILKYIKSGTESGATLESGGEK

LGSKGFYVQPTVFSNVQDNMLIARDEIFGPVQSLLKFKDVEEVIRRANSSHYGLAAGVFTQNIDAANTISRALRVGTVWV

NCFNIFDAAIPFGGYKMSGHGREKGVYSLSNYLQVKAIVTPLKNPAWL

>SlALDH2B7c

MAFRLITSRLSHSSSSSLASLFQGRNSRVAATAALRYTTAAQDPIKPSVNVEYTKLFINGQFVDSASGKTFPTLDPRTGE

VIAHVAEGDVEDINRAVVAARNAFDEGPWPKMSAYERSKILFRIADLIEKHNDEIATLETWDSGKLYQQVATIEIPMIVR

LLRYYAGWADKIHGMTVPADGPYHVQTLHEPIGVVGQIIPWNFPLLMFAWKIGPALACGNTVVLKTAEQTPLSALYVSKL

LQEAGLPEGVLNVISGFGPTAGAALSSHMDVDKLAFTGSTDTGKTIMSLAANSNLKPVTLELGGKSPFIVCEDADVDQAV

EFAHFALFFNQGQCCCAGSRTYVHESIYDEFVEKAKARALKRTVGDPFDSSNEQGPQISSEQFEKVLKYIRSGIESGATL

ETGGDRLGTQGYYIKPTVFSNVKDDMLIATDEIFGPVQSILKFKDHDEVIRRANATKYGLAAGVFTKNIDTANTFMRALR

VGTIWINCFDIFDAAIPFGGYKMSGQGREKGEYSLKQYLQVKAVVTSLKNPAWL

>SlALDH2B7d

MAARVFLSRSVHLLSKGKRSHLGKIAAYKYSTAAAFEEPVKPTVNVDHTKLLINGQFVDSASGKTFPTLDPRTGEVIAHI

AEGDAEDINRAVAAARKAFDEGPWPRMTAYERSKILLRLADLIEKHNDQIATLETWDTGKPYAQAAKIEVPMVVRLLRYY

AGWADKIHGMTIPADGPYHVQTLHEPIGVAGQIIPWNFPLLMFSWKIGPALACGNTIVLKTAEQTPLSALYVASLFQEAG

LPEGVLNIISGYGATAGASLCSHMDVDKLAFTGSTETGKTILELAAKSNLKPVTLELGGKSPFIVCEDADIDTAVEQAHF

ALFFNQGQCCCAGSRTYVHEKVYDEFLEKAKARALKRVVGDPFKSGTEQGPQIDSKQFDKIMKYIRSGVDSGATLETGGE

QFGKKGYYIRPTVFSNVKDDMLIAQDEIFGPVQSILKFKDLDEVVRRANSSRYGLAAGVFSQNIDTANTLARALRVGTVW

INCFDTFDATIPFGGYKMSGQGREKGEYGLKNYLQVKAVVTPLKNPAWL

>SlALDH2C4

MAEMNGNSETQFQIPKIKFTKLFINGEFVDSVSGNTFETIDPRNEEVIARISEGDKEDIDLAVKAAREAFDNGPWPRLSA

AERRRIMLKFADLIIENAEEIAALDAMDAGKLFVPVKNMDIPAAAEIIRYYAGAADKIHGTTLKMSREMQGYTLLEPIGV

VGHIIPWNFPTQMFLMKVGPALAAGCTMIVKPAEQTPLSALYYAQLAKQAGVPDGVINVVTGFGSTAGAALCSHMDVDKI

SFTGSTEVGRLVMQAAALSNLKPVSLELGGKSPFIVFDDVDVDKVAPLALVGILFNKGEICVAGSRLFIQEGIYDKFVKK

LEQMVKTWVVGDPFDPNSHQGPQVDKKQYERVLSYIEHGKREGAKLLTGGNALDRKGYFIEPTIFIDVEDDMKIAKEEIF

GPVLAVMKFKTVEEVIKRANCTNYGLAAGVMTNNLNIANTVSRSIRAGVIWINCYFAFDPDCPYGGYKCSGFERDLGMEG

LHKYLQVKSVATPIYNSPWL

>SlALDH3F1a

MSTTTMTKLCNSYPTTPQIMLECEKELEVLKETFKSGKTKEESWRRSQLKNLLKLLEEKENDIFKALKQDLGKHKVEAYR

DEVGTLVKSVHYALDGLKQWMSPKKAKLPIAAFPSSAELLPEPLGLVLIISSWNFPFSLSLEPLIGAIAAGNVVLLKPSD

QAPASSSVLAKIIPNYLDNKAIKVIEGDYTVGDKLLQQKWDKIFFTGSPKVAQIVMGAAAKHLTPVTLELGGKCPAIIDS

LSSSWDKKIAMKRILSGKFGSCAGQACIGIDYILVDNTFVNELVKLIKLGIPKMLGENPKESHSISRIVNKNQFLRLKNL

LDEPMVKKSIIYGGSSDEDNLYIEPTVLLDPPLQSTIMTDEIFGPLLPIITLDKIEDSIEFINARPKPLTIYAFTKNEEF

KRKITKGTSSGSLVFNDTIIQYAADTLPFGGVGQSGFGRYHGKFSFDTFSHEKAIARRSFLTDIWFRYPPWSDHTLQLFR

SAFIYDYLSVVLITLGLKRA

>SlALDH3F1b

MTGLSLEPLIGAIAYENVALLKPSDQAPASSSVLAKIIPNYLDNKAIKVIEGDYTVGDKLLQQKWDKIFFTGSPKVAQIV

MGAAAKHLNPVTLELGGKCPAIIDSLSSSWDKKIAMRRILSGKFGSCAGQACSGIDYILVDNIFVNELVKLIKLGIPKML

GENPKESHSISRIVIKNQFLRLKNLLDEPMNQRYIEPTVLLYPPVQSTIMIDEIFGPLSPIITLDKIEDNIEFINARPKP

LTIYAFTKNEEFKGKITKGTCSGSLVFSDTIIQDLEQFLPXSLALDHXFXCMRTEKANHLKDKMEELSLNSSNANLVESS

GTVVKDRKVGHRAARCYQRKGQDSKKEGQSDVQANLVEGNEVVVVVVVEANLQANKIGRVLETGASRHFSANKELLHDFE

ESTDRECIYIGDSTTDVVMGLSLEPLIGAIAAGNVALLKPLDQAPASSSVLAKIIPNYLDNKAIKVIEGDYTVKLIKLGI

PIMLGENPNESHSISRIVNKNQFLRLKNLLDEPMVKKSIIYGGSSDEDNLDIEPTVLLDPPVQSTIMADEIFGPLSPIIT

LDKIEDSIEFINARPKPLTIYAFTKNEEFKRKITKRTSSGSLVFNDTIIQYATDTLPFGGVGQSGFGRYHGKFSFDTFSH

EKAIAKRSFLTDIWFRYSPWSDHTLQLFRSAFIYDYISVVLITLGLKRA

>SlALDH3F1c A new protein sequence entered manually

MRTEKANHLKDKMEELSLNSSNANLVESSGTVVKDRKVGHRAARCYQRKGQDSKKEGQSDVQANLVEGNEVVVVVVVEAN

LQANKIGRVLETGASRHFSANKELLHDFEESTDRECIYIGDSTTDVVMGLSLEPLIGAIAAGNVALLKPLDQAPASSSVL

AKIIPNYLDNKAIKVIEGDYTVKLIKLGIPIMLGENPNESHSISRIVNKNQFLRLKNLLDEPMVKKSIIYGGSSDEDNLD

IEPTVLLDPPVQSTIMADEIFGPLSPIITLDKIEDSIEFINARPKPLTIYAFTKNEEFKRKITKRTSSGSLVFNDTIIQY

ATDTLPFGGVGQSGFGRYHGKFSFDTFSHEKAIAKRSFLTDIWFRYSPWSDHTLQLFRSAFIYDYISVVLITLGLKRA

>SlALDH3F1d

MDVVEEDVLGEVTTAFRSRRTRSVAWRKAQLQAILKLLDENEEEIFEALRQDLGKHPVESYRDEVGVVRKSATNALRCVE

KWMAPQKAPIPLVLFPARGAVVSEPLGVVLIFVSWNFPISLALDPVIGAISAGNAIVLKPSELAPKCSSVLANTIPRYLD

PEAIKVVEGGHDVSEQLLQLKWDKIFFTGSPRVGRLIMSAAAKHLTPVTLELGGKCPTILDRLSNFSDLQVAVKRIVGGK

WGPCNGQACIGIDYVLVETQFAPVLIELLEKSIKTFYGENLKTLANLARIVNKHHFDRVHNLLKDPKVAASVVYGGSVDE

ENMAIEPTILLNPPLDADIMNEEIFGPLLPIITLKNIEESIPFINSRPKPLAIYAFTKNDSLKEKILQETSSGSLTFNDA

MIQFLCDTLPFGGVGQSGYGRYHGKFTFDTFSHEKAVLHRSFLVELESRYPPWNDFKMEFVRLAYNYDYLGMILLLLGLR

GLFRTNRRQ

>SlALDH3H1

MDAEAIVKELRGTYGSGKTKSYEWRVSQLKALLKIAENHEKEITDALYSDLSKPELEAFIHEVSMMKTACKLALKELKWW

MKPEKVKTSLTSFPSSAEIVPEPLGVVLVISAWNYPFLLSLDPVIGAIAAGNAVVLKPSEIAPATSSVLAKLLGQYMDVS

AIRVVEGAVPETTALLEQKWDKIFYTGNGKVGRIVLAAAAKHLTPVVLELGGKSPVVVDSNIDYKIAVRRIIAGKWGCNN

GQACISPDYIITTKESVPKLLDAMKQELEKFYGKDPLKSGDLSRIVNANHFQRLSKLLDDNKVVDKVVHGGQRDENNLKI

SPTILLDVPEDSLIMKEEIFGPLLPIITVNKVEDSIQFIKAREKPLAAYLFTSNKKLEEEFVMNISAGGLLINDTTLQVA

LSTLPFGGVGESGMGSCHGKFSFDTFSHKKAVLRRSFAGDVPARYPPYTAGKARFLKALLNGDIIGLIRALIGW

>SlALDH5F1a

MQMIRVRTRMALSACAMLYRSSISGPVRLMTTDTQSVAAKLSSSGLLRSQALIGGKWVDAYDGKTIKVHNPATGEVITDV

PCMGGRETNDAISSAYDAFSSWSKLTAAERSRYLRKWYDLIMAHKEELGQLMTLEQGKPLKEAIGEVSYGAGFIEFSAEE

GKRIYGDIIPSPLADRRLFVLKQPVGVVGAITPWNFPLAMITRKVGPALACGCTVVIKPSELTPLTALAAAELSIQAGIP

PGVVNVVMGNAPDIGDALLASPQVRKITFTGSTKVGKKLMEGAAATVKKVSLELGGNAPCIIFDDADLEVALKGALATKF

RNTGQTCVCANRILVQEGIYDKFANAFAKAVQNMKVGDGFTEGVEQGPLINEAAVQKVEYFVDEATSKGAKVLVGGKRHS

LGMTFYEPTVVTGVNSEMLLAKEEVFGPVAPLLKFKTDEEAIQMANDTNAGLAAYIFSTNIKRAWRVTEALEYGIVGVNE

GLVSTEVAPFGGVKQSGLGREGSKYGMDEYLEMKYVCLGSMS

>SlALDH5F1b

MQMIRVRTRMALSACAMLYRSSISGPVRLMTTDTQSVAAKLSSSGLLRSQALIGGKWVDAYDGKTIKVHNPATGEVITDV

PCMGGRETNDAISSAYDAFSSWSKLTAAERSRYLRKWYDLIMAHKEELGQLMTLEQGKPLKEAIGEVSYGAGFIEFSAEE

GKRIYGDIIPSPLADRRLFVLKQPVGVVGAITPWNFPLAMITRKVGPALACGCTVVIKPSELTPLTALAAAELSIQAGIP

PGVVNVVMGNAPDIGDALLASPQVRKITFTGSTKVGKKLMEGAAATVKKVSLELGGNAPCIIFDDADLEVALKGALATKF

RNTGQTCVCANRILVQEGIYDKFANAFAKAVQNMKVGDGFTEGVEQGPLINEAAVQKVEYFVDEATSKGAKVLVGGKRHS

LGMTFYEPTVVTGVNSEMLLAKEEVFGPVAPLLKFKTDEEAIQMANDTNAGLAAYIFSTNIKRAWRVTEALEYGIVGVNE

GLVSTEVAPFGGVKQSGLGREGSKYGMDEYLEMKYVCLGSMS

>SlALDH6B2

MMQFSVHRVKKLRSLTPGIFAVANHHFSVATESSWKHRTSLRVPNLIGGSFVDSQSSEFVDVINPATQEVVSQIPLTTDK

EFKSAVSAAKEAFPSWKNTPITTRQRVMLKFQELIRKNMDKLAFNVTTEQGKTLKDAQGDVFRGLEVVEHACGMATLQMG

EYGSNVSNGIDTYSLREPLGVCAGICPFNFPAMIPLWMFPVAATCGNTFILKPSEKDPGASMMLAELAMEAGLPDGVLNI

VHGTHDVVNAICDDDDIRAVSFVGSNQAGMHIYSRASAKGKRVQSNMGAKNHGVVMPDANIDSTVNALVGAGFGAAGQRC

MALSTVVFVGDSKPWEEKLLERAKTLKVNAGTEPDADLGPVISKQAKERVCRLVQSGVDSGAKLLLDGRDIVVPGYEKGN

FVGPTILCGVTPDMECYKEEIFGPVLLCMQANSLDEAINIVNQNMYGNGAAIFTTSGVAARKFQTEIESGQIGINVPIPV

PLPFFSFTGSKASFVGDLNFYGKAGVQFYTQIKTVTQQWKDLSSGSGNSLAMPTSQK

>SlALDH7B4a

MTNFTMEEYEFLKELGIGPQNLGCYVNGTWKATGPVISTVNPASNQIIAEVYEASARDYEEGMSACAEAAKIWVQVPAPK

RGEIVRQIGDALRANLQQFGRLVSLEMGKILPEGIGEVQEVIDMCDFAVGLSRQLNGSIIPSERPNHMMLETWNPLGIVG

VITAFNFPCAVLGWNACIALVCGNCVVWKGAPTTPLVTIAMTKIVASVLEKNNLPGSIFTAFCGGAAVGQAIAMDTRIPL

VSFTGSSKVGLAVQQTVSQRFGKCLLELSGNNAIIIMDDADIKLAVRSVLFAAVGTAGQRCTTCRRLLVHESIYDKVLEP

LVDVYKQVKIGDPLEKGTLLGPLHTRTSRENFEKGIHNIKSQGGKILTGGSVVESEGNFVRPTIVEISSKAEIVKEELFA

PVLYVMKFKTFEEAVEINNSVPQGLSSSIFTRNPENIFKWIGPQGSDCGIVNVNIPTNGAEIGGAFGGEKGTGGGREAGS

DSWKQYMRRSTCTINYGSELPLAQGINFG

>SlALDH7B4b

MTNFTMEEYEFLKELGIGPQNLGCYVNGTWKATGPVISTVNPASNQIIAEVYEASARDYEEGMSACAEAAKIWVQVPAPK

RGEIVRQIGDALRANLQQFGRLVSLEMGKILPEGIGEVQEVIDMCDFAVGLSRQLNGSIIPSERPNHMMLETWNPLGIVG

VITAFNFPCAVLGWNACIALVCGNCVVWKGAPTTPLVTIAMTKIVASVLEKNNLPGSIFTAFCGGAAVGQAIAMDTRIPL

VSFTGSSKVGLAVQQTVSQRFGKCLLELSGNNAIIIMDDADIKLAVRSVLFAAVGTAGQRCTTCRRLLVHESIYDKVLEP

LVDVYKQVKIGDPLEKGTLLGPLHTRTSRENFEKGIHNIKSQGGKILTGGSVVESEGNFVRPTIVEISSKAEIVKEELFA

PVLYVMKFKTFEEAVEINNSVPQGLSSSIFTRNPENIFKWIGPQGSDCGIVNVNIPTNGAEIGGAFGGEKGTGGGREAGS

DSWKQYMRRSTCTINYGSELPLAQGINFG

>SlALDH10A8

MAIPNIRIPCRQLFIDGEWREPLKKNRLPIINPANEEIIGYIPAATEEDVDMAVKAARSALRRDDWGSTTGAQRAKYLRA

IAAKVLEKKPELATLETIDNGKPWFEAASDIDDVVACFEYYADLAEALDSKKQTEVKLHLDSFKTHVLREPLGVVGLITP

WNYPLLMTTWKVAPALAAGCAAILKPSELASITSLELGEICREVGLPPGALSILTGLGHEAGSPLVSHPDVDKIAFTGSG

PTGVKIMTAAAQLVKPVTLELGGKSPIVVFDDIHNLDTAVEWTLFGCFWTNGQICSATSRLIIQETIAPQFLARLLEWTK

NIKISDPLEEDCKLGPVISRGQYEKILKFISTAKDEGATILYGGDRPEHLKKGYYIQPTIITDVDTSMEIWKEEVFGPVL

CVKTFKIEEEAIELANDTKFGLGAAILSKDLERCERFTKAFQSGIVWINCSQPCFWQPPWGGKKRSGFGRELGEWSLENY

LNIKQVTQYVTPDEPWAFYKSPSKL

>SlALDH10A9

MANRNVPIPRRQLYIGGEWREPVKKNRIPIINPATEEIIGDIPAATAEDVDIAVEAARKAIARDDWGSTTGAQRAKYLRA

IAAKVLEKKSVLATLESLDSGKTLYESAADMDDVAGCFEYYAGLAEALDSRRMTPVNLNSDSYKSYVLREPLGVVGLITP

WNYPLLMAIWKVAPALAAGCAAILKPSELASITCLELGEICREIGLPSGALNILTGLGPEAGGPLASHPHVDKISFTGSG

PTGSKIMTAAAQLVKPVSLELGGKSPIVVFDDIDNLDIAAEWTLFGIFANTGQVCSATSRLIVQENIASAFMDRLLKWTK

NIKISDPLEEDCKLGPVVSAGQYEKVLKFISNAKSEGATILCGGERPQHLKKGYYVQPTIITDVNTSMEIWKEEVFGPVL

CVKTFKTEEQAIELANDTKYGLGAAVMSKDVKRCERFTKAFQTGIIWINCSQPTFNELPWGGKKRSGFGRDLGKWGLENF

LNIKQVTEYTSAEPLAFYKSPSKN

>SlALDH11A3a

MAGNGVFAEIIDGEVYKYYCEGEWKKSASGKSVAIINPTTRKTQYKVQACTQEEVNKVMEIAKAAQKSWAKTPLWKRAEL

LHKAAAILKEHKAPIAECLVKEIAKPAKDAVTEVVRSGDLVSYTAEEGVRILGEGKFLVSDSFPGNERTKYCLTSKIPLG

VILAIPPFNYPVNLAVSKIAPALIAGNSLVLKPPTQGAVAALHMVHCFHLAGFPKGLISCVTGKGSEIGDFLTMHPGVNC

ISFTGGDTGVAISKKAGMVPLQMELGGKDACIVLEDADLDLAAGNIVKGGFSYSGQRCTAVKVVLVMESVADILVEKVNA

KVAKLTVGPPEDNCDITPVVSESSANFIEGLVMDAKEKDATFCQPYKREGNLIWPLLLDNVRPDMRIAWEEPFGPVLPVI

RINSVEEGIHHCNASNFGLQGCVFTKDINKAILISDAMETGTVQINSAPARGPDHFPFQGIKDSGIGSQGITNSINMMTK

VKTTVINLPTPSYTMGKL

>SlALDH11A3b

MAGNGVFAEIIDGEVYKYYCEGEWKKSASGKSVAIINPTTRKTQYKVQACTQEEVNKVMEIAKAAQKSWAKTPLWKRAEL

LHKAAAILKEHKAPIAECLVKEIAKPAKDAVTEVVRSGDLVSYTAEEGVRILGEGKFLVSDSFPGNERTKYCLTSKIPLG

VILAIPPFNYPVNLAVSKIAPALIAGNSLVLKPPTQGAVAALHMVHCFHLAGFPKGLISCVTGKGSEIGDFLTMHPGVNC

ISFTGGDTGVAISKKAGMVPLQMELGGKDACIVLEDADLDLAAGNIVKGGFSYSGQRCTAVKVVLVMESVADILVEKVNA

KVAKLTVGPPEDNCDITPVVSESSANFIEGLVMDAKEKDATFCQPYKREGNLIWPLLLDNVRPDMRIAWEEPFGPVLPVI

RINSVEEGIHHCNASNFGLQGCVFTKDINKAILISDAMETGTVQINSAPARGPDHFPFQGIKDSGIGSQGITNSINMMTK

VKTTVINLPTPSYTMG

>SlALDH11A4a

MALRMWASSTANALRVSSTVSRTNFSLSRCFSTVLEGLKYASSHEWVKHEGSVATIGITDHAQDHLGEVVFVDLPDSGTS

VSHGSSFGAVESVKATSDINSPISGEIVEVNTKLSETPGLINSSPYEDGWMIKVKPSNPSELESLMGAKEYTKLCDEEEI

H

>SlALDH11A4b

MALRMWASSTANALRVSSTVSRTNFSLSRCFSTVLEGLKYASSHEWVKHEGSVATIGITDHAQDHLGEVVFVDLPDSGTS

VSHGSSFGAVESVKATSDINSPISGEIVEVNTKLSETPGLVKCLLTHISTRALMKTDG

>SlALDH12A1

MMYRLSAYRQLQKRASSSHLNWITLFDSRRSNHTLSFATVKAEEVSGSQPAEVHNLVQGKWTKSSSWNTILDPLNGQPFI

KVAEVNESELQPFVESLSKCPKHGLHNPFKAPERYLMLGDVSTKAAHALGLPEVSDFFAKLIQRVSPKSYQQALVEVLVT

QKFLENFCGDQVRFLARSFAVPGNHLGQQSHGFRWPYGPVAVIAPFNFPLEIPLLQLMGALYMGNKPVLKVDSKVCIVME

QMLRLLHECGLPVDDVDFINSDGKTMNKLLVEAKPRMTLFTGSSRVAEKLADDLSGRVKLEDAGFDWKILGPDVNEVDYV

AWVCDQDAYACSGQKCSAESILFMHENWSKSSLLDKMTELAARRKLDDLTIGPVLTVTTETMLDHAKKLLQIPGSRLLFG

GEALQNHSIPKIYGAIKPTAIFVPLEEILKDEHYPLVTKEIFGPFQVVTEYKDNQLPLVLDALEKMHAHLTAAVVSNDIL

FLQKVIGNSVNGTTYAGLRARTTGAPQNHWFGPAGDPRGAGIGTPEAIKLVWSCHREIIYDVGPMPLGWKVPAST

>SlALDH18B1

MDSADPARAFVKDVKRIIIKVGTAVVTRGDGRLALGRMGSLCEQIRELTSQGFEVILVTSGAVGVGRQRLRYRKLINSSF

ADLQKPQGDLDGKACAAVGQNGLMALYDTLFSQLDVTSAQLMVTDNDFRDPDFRRQLNETVNSLLCLKVVPIFNENDAIS

TRKAPYEDSSGIFWDNDSLAALLALELKADLLVLLSDVEGLYTGPPTDPQSELIHTYVKEKHEGLITFGDKSRVGRGGMT

AKVKAAVYAAYAGIPVVITSGFANNNIIKALDGQRVGTLFHREAIKWASIGDFDAREMAVSARECARRLQTLSSQERSKI

LLDIADALEAKEEEILAENEADVAAAQQAGYENALISRLAMKPGKISSLANSVRVLANMDEPVGRILKRTELADGIILEK

TSSPLGVLLIIFESRPDALVQIASLAVRSGNGLLLKGGKEAKRSNAILHKVITSSIPPIVGERLIGLVTSREEIPELLKL

DDVIDLVIPRGSNKLVSQIKAATKIPVLGHADGICHVFIDKSADLDMAKRIVLDAKTDYPAACNAMETLLVHEDLVQTGG

LNDLILELQEKGVSLFGGPKASSVLNIPEANSFHHEYGALACTVEIVEDVNTAIEHIHRHGSAHTDSIITEDKEVAELFL

RQVDSAAVLHNASTRFSDGFRFGLGAEVGISTSRIHARGPVGVEGLLTTRWLARGSGQVVDGDKEIVYTHKDLNLEA

>SlALDH18B2

MDSADPARAFVKDVKRIIIKVGTAVVTRGDGRLALGRMGSLCEQIRELTSQGFEVILVTSGAVGVGRQRLRYRKLINSSF

ADLQKPQGDLDGKACAAVGQNGLMALYDTLFSQLDVTSAQLMVTDNDFRDPDFRRQLNETVNSLLCLKVVPIFNENDAIS

TRKAPYELSLQDSSGIFWDNDSLAALLALELKADLLVLLSDVEGLYTGPPTDPQSELIHTYVKEKHEGLITFGDKSRVGR

GGMTAKVKAAVYAAYAGIPVVITSGFANNNIIKALDGQRVGTLFHREAIKWASIGDFDAREMAVSARECARRLQTLSSQE

RSKILLDIADALEAKEEEILAENEADVAAAQQAGYENALISRLAMKPGKISSLANSVRVLANMDEPVGRILKRTELADGI

ILEKTSSPLGVLLIIFESRPDALVQIASLAVRSGNGLLLKGGKEAKRSNAILHKVITSSIPPIVGERLIGLVTSREEIPE

LLKLDDVIDLVIPRGSNKLVSQIKAATKIPVLGHADGICHVFIDKSADLDMAKRIVLDAKTDYPAACNAMETLLVHEDLV

QTGGLNDLILELQEKGVSLFGGPKASSVLNIPEANSFHHEYGALACTVEIVEDVNTAIEHIHRHGSAHTDSIITEDKEVA

ELFLRQVDSAAVLHNASTRFSDGFRFGLGAEVGISTSRIHARGPVGVEGLLTTRWLARGSGQVVDGDKEIVYTHKDLNLE

A

>SlALDH19

MALSVQEMGQRAKKATAQVAGLSLATRNTLLKNMGAALLMRQDEIIAANQQDLVAYGASLSRPMQKRLTLDSDALTAIAE

SLAAVATLPDPLAGPYDTWENHAGLKIVKKIVPLGVVAMIYEARPNVTVDAAALALKSGNAVILRGGKEAIHSNTVLATI

LRDVLIDQNLNPDIIQLITDTTHESVNTLLHMREAIDVLIPRGSAAFIDYVVANATVPVIETGAGNTHIFVDASADQAAA

LRIIHNAKTQKPAVCNAAEKLLIHEAIAQEFLPKIADRLIAARVALRGDQASLGIDGRLTPASDADWDTEYNDLVMGIKI

VPDVTAAIDWINTHTTHHSETIISQDPDNIAAFMNQVDAAVVYQNASSRFTDGFEFGFGAEIGISTQKLHARGPMGLPAL

TTIKYEALAMAIRA

>SlALDH22A1

MAFWWPLIVIAIAFAICKLLLMLIPDNVPSIDVDTSDVLDDGNQTKDNSFIYIPSRRHTDKVQCYEPATMKYLGYFPALK

PDEVKERVVQARKAQKIWAKSSFKQRRLFLRILLKYIIEHQDLICNISSRDTGKTMVDASLGEIMTTCEKIHWLLSEGEK

WLKPEYRSCGRSMLHKVAKVEFSPLGVVGAIVSWNYPFHNIFNPMLAAVFSGNSIVIKVSEHASWSGCFYLRIIQTALAA

VGAPENLVEVITGFAETGEALVSSVDKIIFVGSPGVGKKIMRSASDTLIPVTLELGGKDAFIVCEDVDVPHVAQIAARGA

LQSSGQNCAGAERFYVHKDVYSSFVAEVVKIVKSVTAGPPLSGKYDMGAICMQEHSERLQYLVNDALDKGAEIVARGSVG

NIGEGAVDQYFPPTVIVNVNHTMKLMQEEAFGPILPIMKFSSDEEVVQLANDSSYGLGCAVFSGSQRRARQIASQLHCGV

AAVNDFASNYMCQSLPFGGVKDSGFGRFAGIEGLRACCLVKSVVEDRWWPFIKTKIPKPIQYPIAENGFEFQESLVHTLY

GLNIWDRLRALVNVLKILSEQPPAPTSNRRRND

>StALDH2B1

MNPTTKAETNVNRPKSSTFLYCNSNHIASNCRKPSNLVMFEEDGEIDSFLLGGQTHQEYSNYWKKGIRRFCSSAVVHEEP

ITPPVEVKYNQLLINGQFVDAASGKTFPTFDPRTGEAITTVAEANTEDVNRAVSAARKAFDEGPWPKMTAYVGRIKFMVL

QLLLTVCTMFRPCMSQLVLPDR

>StALDH2B2

MAARRLSSLLSRSLHLPSASASLGRSHGVARHINRFSTAAAVEELITPPVQVNHTKLLINGQFVDSASGKTFPTLDPRTG

EVIANVAEGDLEDVNRAVAAARKAFDEGPWPKMSAYERSRIMLKFADLVEKHNDEIAALETWDNGKPYLQAAQAEVPSFV

RLFRYYAGWADKIHGLTVPADGPYHVQTLHEPIGVAGQIIPWNFPLLMMAWKVGPALACGNTIVLKTAEQTPLTALYVAN

LFHEAGLPPGVLNIVSGFGPTAGAALASHMDVDKLAFTGSTETGQTVLQLAAKSNLKPVTLELGGKSPFIICEDADVDHA

VELAHFALFFNQGQCCCAGSRTYVHERVYDEFVEKAKARAMRRVVGDPFKKGVEQGPQIDSEQFKKILRYIREGRDSSAT

LECGGDRIGSKGYFIQPTVFSNVKEDMSIAQDEIFGPVQCVFKFKDIGEVIKRANNTRYGLAAGVFTKNIDTANTLTRGL

RAGTVWINCYDIFDAGIPFGGYKMSGTGREKGIYSLNNYLQVKAVVTPLKNPAWI

>StALDH2B3

MTRSEQKNKCPSFSNTTAEMRFNGRKKYKEMLLAQKEALLLQRRTRDLESRRRLLQVPVMIREPYVTSTKGKAKACVMRP

VVGDPFTKGVEQGPQINSGQFKKTLRCIREGRDSSAALECGDDTICSQSYFIQFKC

>StALDH2B4

MILVCFPSTTLSRKLLLSSPPSEIAPPKPHNNIVNDEDNNPKEKSNLKPVTLELGGKSPFIVCEDADIDTAVEQAHLALF

FNQGQCCCAGSQTYVHEKVYDEFLEKAKARTLKRVVGDPFKSGTEQGPQDDMLIAQDEIFGPVQSILKFKDLDEVVRRAN

SSRYGLAAGVFSQNIDTANTLARALRVGMVWINCFDTFDATIPFGGYKMSGQGREKGEYDLRNYLQVKAVATALKNPTWL

>StALDH2B5

MLPYLWQRTETKRAKELSTTTNRLQQTRKRDLDEMVRRDNSSQYGLAAGVFSQNIDTANTLARASRVGMVWINCFDTFNS

TIPFGGYKMSGQGREKGEYGLRNYLQVKSVVTPLKSPAWL

>StALDH2B6

MAARVFLSRSVHLLSKGKRSHLGRIAAYKYSTAAALEEPIKPTVNVDHTKLFINGQFVDSASGKTFPTLDPRTGEVIAHI

AEGDAEDINRAVAAARKAFDEGPWPRMTAYERSKILLRLADLIEKHNDQIATLETWDTGKPYAQAAKIEVPMVVRLLRYY

AGWADKIHGMTIPADGPYHVQTLHEPIGVAGQIIPWNFPLLMFSWKIGPALACGNTIVLKTAEQTPLSALYVANLLQEAG

LPEGVLNIISGFGATAGASLCSHMDVDKLAFTGSTETGKTILELAAKSNLKPVTLELGGKSPFIVYEDADIDTAVEQAHF

ALFFNQGQCCCAGSRTYVHEKVYDEFLEKAKARALKRVVGDPFKSGTEQGPQIDSKQFDKIMKYIRSGVDSGATLETGGE

QFGKKGYYIKPTVFSNVKDDMLIAQDEIFGPVQSILKFKDLDEVVRRANSSRYGLAAGVFSQNIDTANTLARALRVGTVW

INCFDTFDATIPFGGYKMSGQGREKGEYGLRNYLQVKAVVTPLKNPAWL

>StALDH2B7

MGKGTGSFGKRRNKTHTLCVRCGRRSFHIQKSRCSACAYPAARLRKYNWSVKALRRKTTGTGRMRYLRNVPRRFKTNFRE

GRNSRVAATAALRYTTAAPIAQDPIKPSVNVEYTKLFINGQFVDSTSGKTFPTLDPRTGEVIAHVAEGDVEDINRAVVAA

RNAFDEGPWPKMSAYERSKVLFRIADLIEKHNDEIATLETWDSGKLYQQVATIEIPMIVRILRYYAGWADKIHGMTVPAD

GPYHVQTLHEPIGVVGQIIPWNFPLLMFAWKIGPALACGNTVVLKTAEQTPLSALYVSKLLQEAGLPEGVVNVISGFGPT

AGAALCSHMDVDKLAFTGSTDTGKTIMSLAANSNLKPVTLELGGKSPFIVCEDADVDQAVEFAHFALFFNQGQCCCAGSR

TYVHESIYDEFVEKAKARALKRTVGDPFESGNEQGPQISSEQFEKVLKYIRSGIESGATLETGGDRLGTRGYYIKPTVFS

NVKDDMLIATDEIFGPVQSILKFKDHDEVIRRANATKYGLAAGVFTKNIDTANTFMRALRVGTIWINCFDIFDAAIPFGG

YKMSGQGREKGEYSLKQYLQVKAVVTSLKNPAWL

>StALDH2C1

MAESNGNSETQFQIPKIKFTKLFINGEFVDSVSGNTFETIDPRNEEVIARISEGDKEDVDLAVKAAREAFDDGPWPRLSP

SERRRIMLKFADLIVENAEEIAALDAMDAGKLFAPVKNMDIPAAAEVIRYYAGAADKIHGTTLKMSCELQGYTLLEPIGV

VGHIIPWNFPTQMFVMKVGPALAAGCTMIVKPAEQTPLSALYYAQLAKQAGVPDGVINVVTGFGSTAGAALCSHMDVDKI

SFTGSTEVGRLVMQAAALSNLKPVSLELGGKSPFIVFDDVDVDKVAPLALVGILYNKGEICVAGSRLFIQEGIYDKFVKK

LEEMAKTWVVGDPFDPNSHQGPQVDKKQYERVLSYIEHGKREGAKLLTGGNALDRKGYFIEPTIFIDVEDDMTIAKEEIF

GPVLAVMKFKTVEEVIKRANCTNYGLAAGVMTNDLNIANTVSRSIRAGVIWINCYFAFDPDCPYGGYKCSGFERDLGMEG

LHKYLQVKSVATPIYNSPWL

>StALDH3F1

MGAAAKHLTPVTLELGGKCPVIIDSLSSSWDKKIAIKRILSGKFGSCAGQACIGIDYILVDKTFVNELVKLIKLGIPKMF

GENPKESHSISRIVNRNHFLRLKNLLDEPMVKKSVIYGGSSDEDNLYIEPTVLLDPPMQSTIMTEEIFGPLLPIITLDKI

EDSIEFINARPKPLTIYAFTKNEEFKRKITKGTSSGSLVFNDTIIQYAADTLPFGGVGQSGFGRYHGKFSFDTFSHEKAI

ARRSFLTDIWFRYPPWSDHTLQLFKSAFIYDYLSIVLITLGLKKA

>StALDH3F2

MNGVEEDVLGELRTTFRSGRTRSVAWRKAQLQAILKLLDENEEEIFEALKQDLGKHPVESYRDEVGVVRKSATNALRCVE

KWMAPQKAPIPLVLFPARGAVVSEPLGVVLLFVSWNFPISLTLDPAIGAISAGNTIVLKPSELAPKCSSVLANTIPRYLD

PEAIKVVEGGQDVSEQLLQLKWDKIFFTGSPRVGRLIMSAAAKHLTPVTLELGGKCPTILDTLSSSYDLQVAVKRIAGGK

WGPCNGQACIGIDYVLVETQFAPVLIELLEKIIKTFYGENLKTLGNLARIVNKHHFDRVHNLLKDPKVAASVVYGGSVDE

ENMVIEPTILLNPPLDADIMTEEIFGPLLPIITLNNIEESIQFINSRPKPLAIYAFTKNDSLKEKILQETSSGSLTFNDA

MIQFICDTLPFGGVGQSGYGRYHGKFSFDTFSHEKAVLHRSLLIELESRYPPWNNFKLEFVRLAYDYDYLGLILLLLGLR

GIFRTNRRQ

>StALDH3H1

MDAEAIVKELRGTYGTGKTKSYEWRVSQLKALFKIAENHEKEITDALYSDLSKPELEAFIHEISMMKTACKLALKELKRW

MKPEKVKTSLTSFPSSAEIVPEPLGVVLVISAWNYPFLLSLDPVIGAIAAGNAVVLKPSEIAPATSSVLAKLLGQYMDVS

AIRVVEGAVPETTALLEQKWDKIFYTGNGKVGRIVLAAAAKHLTPVVLELGGKSPVVVDSNIDYKIAVRRIIAGKWGCNN

GQACISPDYIITTKENVPKLLDAMKQELEKFYGKDPLKSGDLSRIVNANHFQRLSKLLDDKKVVDKVVHGGQRDEDNLKI

SPTILLDVPEDSLIMKEEIFGPLLPIITVNKVEDSIQFINAREKPLAAYLFTSNKKLEEEFVMNISAGGLLINDTTLQVA

LSTLPFGGVGESGMGSCHGKFSFDSFSHKKAVLRRSFAGDVPARYPPYTTGKARFLKALLNGDILGLIRALIGW

>StALDH5F1

MALSACAMLHRSSISGPVRLMTTATQSIAAKLSSSGLLRSQALIGGKWVDAYDGKTIKVHNPATGEVITDVPCMGGRETN

DAISSAYDAFSSWSKLTAAERSKYLRKWYDLIMAHKEELGQLMTLEQGKPLKEAIGEVSYGAGFIEFSAEEGKRIYGDII

PSPLADRRLFVLKQPVGVVGAITPWNFPLAMITRKVGPALACGCTVVIKPSELTPLTALAAAELSIQAGIPPGVVNVVMG

NAPAIGDALLASPQVRKITFTGSTKVGKKLMEGAAATVKKVSLELGGNAPCIIFDDADLEVALKGALATKFRNTGQTCVC

ANRILVQEGIYDKFANAFAKAVQNMKVGDGFTEGVEQGPLINEAAVQKVESFVEEATSKGAKVLVGGKRHSLGMTFYEPT

VVTGVNSEMLLAKEEVFGPVAPLLKFKTDEEAIQMANDTNAGLAAYIFSTNIKRAWRVTEALEYGIVGVNEGLVSTEVAP

FGGVKQSGLGREGSKYGMDEYLEMKYVCLGSMS

>StALDH6B1

MMQFSVHRVRKVRSLTPGIFALANHHFSVATESSWKHRTSLRVPNLIGGSFVDSQSSEFVDVINPATQEVVSQIPLTTDK

EFKSAVSAAKEAFPSWKNTPITTRQRVMLKFQELIRKNMDKLAFNVTTEQGKTLKDAQGDVFRGLEVVEHACGMATLQMG

EYVSNVSNGIDTYSLREPLGVCAGICPFNFPAMIPLWMFPVAATCGNTFILKPSEKDPGASMMLAELAMEAGLPDGVLNI

VHGTHDVVNAICDDDDIRAISFVGSNTAGMHIYSRASAKGKRVQSNMGAKNHGVVMPDANIDSTINALVAAGFGAAGQRC

MALSTVVFVGDSKPWEEKLLERAKTLKVSAGTEPDADLGPVISKQAKERVCQLVQSGVDSGAKLLLDGRDIVVPGYEKGN

FVGPTILSGVTPDMECYKEEIFGPVLLCMQANSLDEAINIVNQNKYGNGAAIFTTSGVAARRFQTEIESGQIGINVPIPV

PLPFFSFTGSKASFAGDLNFYGKAGVQFYTQIKTVTQQWKDLSGGSGVSLAMPTSQK

>StALDH6B2

MGTKNYAVVMPYTNVEATLNVLVAAGFGVAGERCTTISTGLCWRLKITVKERISKLIQAIVDSGAKLVLDGRQVAVPKLL

SSLLNICG

>StALDH7A1

MTSFTKKEYEFLKELGIGPQNLGCYVNGTWKATGPVISTFNPANNQIIAEVVEASAQDYEEGMSACAEAAKIWVQVPAPK

RGEIVRQIGDALRANLQEFGRLVSLEMGKILPEGIGEVQEVIDMCDFAVGLSRQLNGSVIPSERPNHMMLEMWNPLGIVG

VITAFNFPCAVLGWNACIALVCGNCVVWKGAPTTPLVTIAMTKIVASVLEKNNLPGSIFTAFCGGADVGQAIAKDTRIPL

VSFTGSSKVGLAVQQTVSQRFGKCLLELSGNNAIIIMDDADIKLAVRSVLFAAVGTAGQRCTTCRRLLVHESIYEKVLEP

LVDVYKQVKIGDPLEKGTLLGPLHTCTSRENFEKGIHNIKSQGGKILTGGSVVESEGNFVHPTIVEISSKAEIVKEELFA

PVLYVMKFKTFEEAVEINNSVPQGLSSSIFTRNPENIFKWIGPQGSDCGIVNVNIPTNGAEIGGAFGGEKGTGGGREAGS

DSWKQYMRRSTCTINYGSELPLAQGINFG

>StALDH10A1

MAIPNIRIPCRQLFIDGEWREPLKKNRLPIINPANEEIIGYIPAATEEDVDIAVKAARSALRRDDWGSTTGAQRAKYLRA

IAAKVLEKKPELATLETIDNGKPWFEAASDIDDVVACFEYYADLAEALDSKKKTEVKLHLDSFKTHVLREPLGVVGLITP

WNYPLLMTTWKVAPALAAGCAAILKPSELASITSLELGEICREVGLPPGALSILTGLGHEAGSPLVSHPDVDKIAFTGSG

PTGVKIMTAAAQLVKPVTLELGGKSPIVVFDDIHDLDIAVEWTLFGCFWTNGQICSATSRLIIQETIAPQFLARLLEWTK

NIKISDPLEEDCKLGPVISRGQYEKVLKFISTAKDEGATILYGGDRPEHLKKGYYIQPTIITDVDTSMEIWNEEVFGPVL

CVKTFKTEEEAIELANDTKYGLGAAILSKDLERCERFTKAFQSGVVWINCSQPCFWQPPWGGKKRSGFGRELGEWSLENY

LNIKQVTQYVTPDEPWAFYKSPSKL

>StALDH10A2

MANRNVPISRRQLYIGGEWREPVKKNRIPIINPATEEIIGDIPAATAEDVDIAVEAARKAIARDDWGSTTGAQRAKYLRA

IAAKVLEKKSVLATLESLDSGKTLFESAADMDDVAGCFEYYADLAEALDSRRKTPVNLNSDSFKTYVLREPLGVVGLITP

WNYPLLMAIWKVAPALAAGCAAILKPSELASVTCLELGEICREIGLPSGALNILTGLGPEAGGPLASHPHVDKISFTGSG

PTGSKIMTAAAQLVKPVSLELGGKSPIVVFDDIDNLDIAAEWTLFGIFANTGQVCSATSRLIVQESIASAFLDRLLKWTK

NIKISDPLEEDCKLGPVVSAGQYEKVLKFISNAKSEGATILYGGKRPQHLKKGYYVQPTIITDVNTSMEIWKEEVFGPVL

CVKTFKTEEEAIELANDTKYGLAAAVMSKDVKRCERFTKAFQTGIIWINCSQPTFNQLPWGGKKRSGFGRDLGEWGLESF

LNIKQVTEYTSAEPWAFYKSPSRN

>StALDH11A1

MAGNGVFAEIIDGEVYKYYCEGEWRKSASGKSVAIINPTTRKTQYKVQACTQEEVNKVMEIAKAAQKSWAKTPLWKRAEL

LHKAAAILKEHKAPIAECLVKEIAKPAKDAVTEVVRSGDLVSYTAEEGVRILGEGKFLVSDSFPGNERTKYCLTSKIPLG

VILAIPPFNYPVNLAVSKIAPALIAGNSLVLKPPTQGAVAALHMVHCFHLAGFPKGLISCVTGKGSEIGDFLTMHPGVNC

ISFTGGDTGVAISKKAGMVPLQMELGGKDACIVLEDADLDLAAGNIVKGGFSYSGQRCTAVKVVLVMESVADTLVEKVNA

KVAKLTVGPPEDNCDITPVVSESSANFIEGLVMDAKEKDATFCQPYKREGNLIWPLLLDNVRPDMRIAWEEPFGPVLPVI

RINSVEEGIHHCNASNFGLQGCVFTKDINKAILISDAMETGTVQINSAPARGPDHFPFQGIKDSGIGSQGITNSINMMTK

VKTTVINLPTPSYTMG

>StALDH12A1

MYRLSAYRQLKNRASSSHLNWITLFNSTRSNHTLSFATVKAEEVSGSQPAEVHNLVQGKWTKSSSWNTILDPLNGQPFIK

VAEVNESELQPFVESLSKCPKHGLHNPFKAPERYLMLGDVSTKAAHALGLPEVSDFFAKLIQRVSPKSYQQALIEVLVTQ

KFLENFCGDQVRFLARSFAVPGNHLGQQSHGFRWPYGPVAVIAPFNFPLEIPLLQLMGALYMGNKPVLKVDSKVCIVMEQ

MLRLLHECGLPVDDVDFINSDGKTMNKLLVEAKPRMTLFTGSSRVAEKLADDLSGRVKLEDAGFDWKILGPDVNEVDYVA

WVCDQDAYACSGQKCSAESILFMHENWSKSSLIDKMTELAARRKLDDLTIGPVLTVTTETMLDHAKKLLQIPGSRLLFGG

EALQNHSIPKIYGAIKPTAIFVPLEEILKDEHYPLVTKEIFGPFQVVTEYKDNQLPLVLDALEKMHAHLTAAVVSNDILF

LQKVIGNSVNGTTYAGLRARTTGAPQNHWFGPAGDPRGAGIGTPEAIKLVWSCHREIIYDVGPMPLGWKVPAST

>StALDH18A1

MDSADPARAFVKDVKRIIIKVGTAVVTRGDGRLALGRMGSLCEQIRELTSQGFEVILVTSGAVGVGRQRLRYRKLINSSF

ADLQKPQGDLDGKACAAVGQNGLMALYDTLFSQLDVTSAQLMVTDNDFRDPDFRRQLNETVNSLLCLKVVPIFNENDAIS

TRKAPYEDSSGIFWDNDSLAALLAMELKADLLVLLSDVEGLYTGPPSDPQSELIHTYVKEKHEGLITFGDKSRVGRGGMT

AKVKAAVYAAYAGIPVVITSGFANNNIIKALDGQRVGTLFHREAIKWASIGDFDAREMAVSARECARRLQTLSSQERSKI

LLDIADALEAKEEEILAENEADVAAAQQSGYENSLISRLAMKPGKISSLANSVRVLANMDEPVGRILKRTELADGIILEK

TSSPLGVLLIIFESRPDALVQIASLAVRSGNGLLLKGGKEAKRSNAILHKVITSSIPPTVGERLIGLVTSREEIPELLKL

DDVIDLVIPRGSNKLVSQIKAATKIPVLGHADGICHVFIDKSADLDMAKRIVLDAKTDYPAACNAMETLLVHEDLVQTGG

LNDLILELQVKGVSLFGGPKASSVLSIPEANSFHHEYGALACTVEIVEDVNTAIEHIHRHGSAHTDSIITEDKEVAELFL

RQVDSAAVLHNASTRFSDGFRFGLGAEVGISTSRIHARGPVGVEGLLTTRWLARGSGQVVDGDKEIVYTHRDLNLEA

>StALDH18A2

MDSADPARAFVKDVKRIIIKVGTAVVTRGDGRLALGRMGSLCEQIRELTSQGFEVILVTSGAVGVGRQRLRYRKLINSSF

ADLQKPQGDLDGKACAAVGQNGLMALYDTLFSQLDVTSAQLMVTDNDFRDPDFRRQLNETVNSLLCLKVVPIFNENDAIS

TRKAPYEDSSGIFWDNDSLAALLAMELKADLLVLLSDVEGLYTGPPSDPQSELIHTYVKEKHEGLITFGDKSRVGRGGMT

AKVKAAVYAAYAGIPVVITSGFANNNIIKALDGQRVGTLFHREAIKWASIGDFDAREMAVSARECARRLQTLSSQERSKI

LLDIADALEAKEEEILAENEADVAAAQQSGYENSLISRLAMKPGKISSLANSVRVLANMDEPVGRILKRTELADGIILEK

TSSPLGVLLIIFESRPDALVQIASLAVRSGNGLLLKGGKEAKRSNAILHKVITSSIPPTVGERLIGLVTSREEIPELLKL

DDVIDLVIPRGSNKLVSQIKAATKIPVLGHADGICHVFIDKSADLDMAKRIVLDAKTDYPAACNAMETLLVHEDLVQTGG

LNDLILELQVKGVSLFGGPKASSVLSIPEANSFHHEYGALACTVEIVEDVNTAIEHIHRHGRQVTFLITIQF

>StALDH22A1

MAFWWPLIVIAIAFAICKLLLMLIPDNVPSIDVDTSDVLDDGNQAKDNSFIYIPSRRHTDKVQCYEPATMKYLGYFPALK

PDEVKERVVQARKAQKIWAKSSFKQRRLFLRILLKYIIEHQDLICNISSRDTGKTMVDASLGEIMTTCEKIHWLLSEGEK

WLKPEYRSCGRSMLHKVAKVEFSPFGVVGAIVSWNYPFHNIFNPMLAAVFSGNSIVIKVSEHASWSGCFYLRIIQTALAA

VGAPENLVEVITGFAETGEALVSSVDKIIFVGSPGVGKKIMRSASNTLIPVTLELGGKDAFIVCEDVDVPHVAQIAARGA

LQSSGQNCAGAERFYVHKDVYSSFVAEIVKIVKSVTAGPPLSGKYDMGAICMQEHSERLQYLVNDALDKGAEIVARGSVG

NIGEGAVDQYFPPTVIVNVNHTMKLMQEEAFGPILPIMKFSSDEEVVQLANDSSYGLGCAVFSGSQRRARHIASQLHCGV

AAINDFASNYMCQSLPFGGVKDSGFGRFAGIEGLRACCLVKSVVEDRWWPFIKTKIPKPIQYPIAENGFEFQESLVHTLY

GLNIWDRLRALVNVLKILSQQPPAPTSNRRRND

>ZmALDH2B2

MARRAASSLVSRCLLARAPAGAPPAAPSAPRRTVPADGMHRLLPGVLQRFSTAAAVEEPITPSVHVNYTKLLINGNFVDS

ASGKTFPTLDPRTGEVIAHVAEGDAEDINRAVAAARKAFDEGPWPKMTAYERSRILLRFADLIEKHNDELAALETWDNGK

PYEQAAQIEVPMVARLMRYYAGWADKIHGLIVPADGPHHVQILHEPIGVAGQIIPWNFPLLMYAWKVGPALACGNTLVLK

TAEQTPLSALYISKLLHEAGLPEGVVNVVSGFGPTAGAALASHMDVDKIAFTGSTDTGKIILELAAKSNLKTVTLELGGK

SPFIIMDDADVDHAVELAHFALFFNQGQCCCAGSRTFVHERVYDEFVEKAKARALKRVVGDPFRKGVEQGPQIDDEQFNK

ILRYIRYGVDGGATLVTGGDRLGDKGFYIQPTIFSDVQDGMKIAQEEIFGPVQSILKFKDLNEVIKRANASQYGLAAGVF

TNSLDTANTLTRALRAGTVWVNCFDVFDAAIPFGGYKMSGIGREKGVDSLKNYLQVKAVVTPIKNAAWL

>ZmALDH2B5

MAATVRRAASSVLSRFLLTKPSPSPASAAGNKSALLGAGAAALHRFSTAPASAAAAAEEPIQPAVEVKHTQLLINGNFVD

AASGKTFPTLDPRTGEVIARVAEGDSEDIDRAVAAARRAFDEGPWPRMTAYERCRVLLRFADLIERHAEEVAALETWDNG

KTLAQAAGAEVPMVARCVRYYAGWADKIHGLVAPADGAHHVQVLHEPVGVAGQIIPWNFPLLMFAWKVGPALACGNTVVL

KTAEQTPLSALYVANLLHEAGLPEGVLNVVSGFGPTAGAALCSHMGVDKLAFTGSTGTGQIVLELAARSNLKPVTLELGG

KSPFIVMDDADVDQAVELAHQAVFFNQGQCCCAGSRTFVHERVYDEFVEKSKARALKRVVGDPFRDGVEQGPQIDGEQFN

KILRYVQSGVDSGATLVAGGDRVGDRGFYIQPTVFADAKDEMKIAREEIFGPVQTILKFSGVEEVIRRANATPYGLAAGV

FTRSLDAANTLSRALRAGTVWVNCYDVFDATIPFGGYKMSGVGREKGIYALRNYLQTKAVVTPIKNPAWL

>ZmALDH2C1

MATANGSSKGPFEVPKVEVRFTKLFIDGKFVDAVSGKTFETRDPRTGEVIASIAEGGKADVDLAVKAAREAFDNGPWPRM

TGYERGRILHRFADLIDEHVEELAALDTVDAGKLFAVGKARDIPGAAHLLRYYAGAADKVHGATLKMAQRMHGYTLKEPV

GVVGHIVPWNYPTTMFFFKVGPALAAGCAVVVKPAEQTPLSALFYAHLAREAGVPAGVLNVVPGFGPTAGAAVAAHMDVD

KVSFTGSTEVGRLVMRAAAESNLKPVSLELGGKSPVIVFDDADLDMAVNLVNFATYTNKGEICVAGTRIYVQEGIYDEFV

KKAAELASKSVVGDPFNPSVSQGPQVDKDQYEKVLRYIDIGKREGATLVTGGKPCGDKGYYIEPTIFTDVKDDMTIAQDE

IFGPVMALMKFKTVEEVIQKANNTRYGLAAGIVTKNIDVANTVSRSIRAGAIWINCYFAFDPDAPFGGYKMSGFGKDMGM

DALDKYLQTKTVVTPLYNTPWL

>ZmALDH2C2

MASNGCNGNGNGNGNGKAAPAGVVVPEIKFTKLFINGEFVDAASGKTFDTRDPRTGDVLAHVAEADKADVDLAVKSARDA

FEHGKWPRMSGYERGRIMSKLADLVEQHTEELAALDGADAGKLLLLGKIIDIPAATQMLRYYAGAADKIHGDVLRVSGRY

QGYTLKEPIGVVGVIIPWNFPTMMFFLKVSPALAAGCTVVVKPAEQTPLSALYYAHLAKMAGVPDGVINVVPGFGPTAGA

ALASHMDVDSVAFTGSTEVGRLIMESAARSNLKTVSLELGGKSPLIIFDDADVDMAVNLSRLAVFFNKGEVCVAGSRVYV

QEGIYDEFVKKAVEAARSWKVGDPFDVTSNMGPQVDKDQFERVLKYIEHGKSEGATLLTGGKPAADKGYYIEPTIFVDVT

EDMKIAQEEIFGPVMSLMKFKTVDEVIEKANCTRYGLAAGIVTKSLDVANRVSRSVRAGTVWVNCYFAFDPDAPFGGYKM

SGFGRDQGLAAMDKYLQVKSVITALPDSPWY

>ZmALDH2C4

MASNGNGDGTARVVVPEIKFTKLFINGEFVDAASGKTFETRDPRTGDVLAHVAEADQADVDLAVKSARDAFDHGKWPRMS

GYERGRVMSKLADLVEQHTEELAALDGADAGKLLLLGKMIDIPAATQMLRYYAGAADKIHGDVLRVSGKYQGYTLKEPIG

VRRYLSS

>ZmALDH2C5

MVSESNRGGADRTTAAGEERGQLLFDVPEIRFTKLFINGSFVDAVSGRTFETRDPRTGGVIASVAEADKEDVDLAVRAAR

AAFDHGEWPRMSGSERGRIMARLADLVEERADELAALESLDAGKHPAVTRAVDVGNAAGSLRYFAGAADKIHGETLKMPG

QFQGHTLREPLGVAGVIIPWNFPSTMFAVKVAPALAAGCALVVKPAEQTPLSALYLAQLAKQAGVPDGVINVVPGFGPTA

GAALASHMDVDMVSFTGSTEVGRLIMKASAESNLKPVYLELGGKSPLIVFDDADLDMAVELAVGASFFNKGEACVAASRV

YVQERVYDRFEERLAERMRSWVVGDPFSDPSADQGPQVDKAQYERVLSYIDHGKREGATLLTGGRPCGPEGKGYYIEPTV

FTNVKEDMIIAKEEIFGPVMCLMKFKTVEEAIARANDTRYGLGAGVVTRDLDVANRVVRSVRAGVVWVNCYFAMGSDCPF

GGRKMSGFGKDEGMHALDKYLAVKSVVTPLRASPWI

>ZmALDH3E1

MGSVPEEKAKLGFGGLVGDLREVYESGRTQGLEWRQSQLRGLVRLLEEKEEEIFDVLHEDLGKHRGEAFRDEVGVLKKSV

VDKLQNLKNWAAPEKAHTPLVAFPATALVVPEPLGVVLVFSCWNLPIGLALEPLSGALAAGNAVVVKPSELAPATSAFLA

ANIPKYLDSKAVKVVEGGPEVGEKLMEHRWDKVLFTGSSRVGRLIMAQAAKHLTPVALELGSKCPCIVDWLDSDRDSQVA

VNRIIGAKWSTCSGQACIAIDYLLVEEEFAPILIEMLKSTLERFFTKPEYMARILNEKQFQRLSGFLADRRVASSVVHGG

HFNPKTLSMEPTLLLNPPLDSDIMTEEIFGPLLPIITVKKIEDSIKFLRSKPKPLAIYAFTRNEKLKQRIIDETSSGSIT

FNDAIVQYGLDSIPFGGVGHSGFGQYHGKYSFDMFSHKKAVLKRSFLVEFMFRYPPWDETKIGMLRRVYRFDYVSLFLAL

IGLRR

>ZmALDH3E2

MGRTEAADDGAESGGLGLGVGVGVGETVRELREAYESGRTRSLAWRQAQLRGLLRLLEEKEVEAFQALHKDLGKHHAEAY

RDEVGVLIKSANGALQQLGKWMAPEKVRVPLIAWPATAQVVPEPLGVVLVFSCWNVPLGLSLEPLIGAIAAGNAVALKPS

ELSPCTARFLGDNIGRYMDSSAVKVVQGGPDVGVQLMEHRWDKVLFTGSPRIARAVMAAASRHLTPVALELGGKCPCIFD

AMGSARDLQISVNRMIAGKWSSCAGQACIAIDYVLVEERFAPILIKVLKSTLKRFFPEADHMARIVNERHFERLSNLLKD

RSVAPSVLHGGSMDSKNLYIEPTILLNPPLDSAIMTEEIFGPLLPIITVKNIEDSIAFVKAMPKPLAIYAFTRDAALRRR

IVDETSSGSVTFNDAVVQYAIDGLPFGGVGQSGFGQYHGKYSFEMFSHKKAVMKRGYLVELTLRYPPWDESKVTLMRYLY

RFNYFAFVLSFLGLRR

>ZmALDH3H1

MDAEAAAAAAATAVEERERLRRSFASGRTRPAAWREAQLRGLLRMATEREDDICAALHADLAKPLTECYVHEISLVISSC

KFALKNLKKWMKPRKVPGGLLTFPSAASVAAEPLGVVLVISAWNYPFLLAIDPVVGAFAAGNAVALKPSEVAPATSLLLA

DLLPRYVDPSCVRVVQGGIAETTALLELQWDKIFYTGNSRVGRIVMSYAAKHLTPVVLELGGKCPVVVDSDVNLHVAAKR

IAAGKWGCNSGQACVSPDYVVTTKSFAPKLLESLKRVLFEFYGEEPLRSPDLSRVVNSNHFNRLMALMDDYSVSGNVAFG

GQIDERRLRIAPTLLLDVPLDSAMMKEEIFGPLLPIITVDKIGESFAVINSMPKPLAAYLFSNDGQLKQQFERTVSAGGI

MFNDTGIHLTNPNLPFGGVGESGMGAYHGAFSFDAFSHRKAVLDRSFLGEARARYPPYTPAKLAILRGVLNGSPLATVQA

AAGCTGGASAD

>ZmALDH3H2

MAEETVRELRASFAAGQTRPAEWRAAQLKGLIRMIDEKEAEISAALHEDLAKPHMESFLHEISLTKSSCKFALKGLKNWM

KPEKVPAAITTFPSSAQIVPEPLGVVLIISAWNYPFILSIDPVIGAIAAGNAVVLKPSEIAPATSSLLAKLLPEYVDNSC

IKVVEGSVPETTALLEQRWDKIFYTGNGTVGRIVMAAAAKHLTPVALELGGKSPVIVDSNVDLHVAAKRIVVGKWGCNNG

QACIAPDYIITTKSFAPELVASFKRVLERFYGEDPLESADLSRIVNSKQFKRLTNLIEEKRVADKIVYGGKADEKQLKIS

PTLLLDVPEDSEIMTGEIFGPLLPIVTVEKIEESFDLINAKPKPLAAYLFTKNRKLQEEFVASVPAGGMLVNDTALHLTN

PYMPFGGVGDSGMGCYHGKFGFDCFSHKKGVLIRGFGGEANARYPPYTTEKQKILRGLINGSFIALILALLGFPREKR

>ZmALDH3H3

MAEETVQELRASFASGRTRRAEWRAEQLKGLIRMIDEKEAEISAALHEDLAKPHMESYLHEISITRSSCKFALDGLKSWM

KPEKIPAALTTFPSSAQIVPEPLGVVLIISAWNYPFILSIDPVIGAIAAGNAVVLKPSEIAPATSSLLAKLLPEYVDNSC

IKVVEGGVAETTSLLEQRWDKIFYTGNGTVGRIVMAAAAKHLTPVALELGGKSPVVVDSNVDLHVAVKRIVVGKWGCNNG

QACIAPDYIITTKSFAPELVASLKRVLERFYGEDPLQSADLSRIVNSKHFRRLTELIEEKSVADKIVYGGEVDEKQLKIA

PTLLLDVPQDSAIMTGEIFGPLLPIVTVEKIEESFDLINARPKPLAAYLFTKNKKLQEEFVADVPAGGMLVNDTVLHLAN

PYMPFGGVGDSGMGCYHGKFGFDCFSHKKGVLVRGFGGEANARYPPYTTEKQKILRGLINGSFIALILALLGFPREKR

>ZmALDH5F1

MATAMMTMRRAAALGARHIPPAAAFSRHMSADASAAMEKIRAAGLLKTQGLIAGQWVDAYDGKTIEVQNPATGEVLANVS

FMGSRETSDAIASAHSTFYSWSKLTASERGKALRKWYDLIISHKEELALLMTLEQGKPMKEALGEVNYGASFIEYFAEEA

KRIYGDIIPPTLSDRRLLVLKQPVGVVGAITPWNFPLAMITRKVGPALACGCTVVVKPSEFTPLTSLAAADLALQAGIPA

GALNVVMGNAPEIGDALLQSTQVRKITFTGSTAVGKKLMAESANTVKKVSLELGGNAPCIVFDDADIDVAVKGSLAAKFR

NSGQTCVCANRILVQEGIYEKFASAFIKAVQSLKVGNGLEESTSQGPLINEAAVQKVEKFINDATSKGANVMLGGKRHSL

GMSFYEPTVVGNVSNDMLLFREEVFGPVAPLIPFKTEEEAVHMANDTNAGLAAYIFTKSIPRSWRVSESLEYGLVGVNEG

IISTEVAPFGGVKQSGLGREGSKYGVDEYLELKYICMGNLG

>ZmALDH5F2

MAMAMMAMRRAVALGARHIPAAAASSFRVVSLRHMSADAGAAMEKIRAAGLLRTQGLIAGQWVDAYDGKTIEVQNPATGE

VLANVSCMGSRETSDAIASAHSTFYSWSKLTASERSKALRKWYDLIISHKEELALLMTLEQGKPMKEALGEVNYGASFIE

YFAEEAKRIYGDIIPPTLSDRRLLVLKQPVGVVGAITPWNFPLAMITRKVGPALACGCTVVVKPSEFTPLTALAAADLAL

QAGIPAGALNVVMGNAAEIGDALLQSTQVRKITFTGSTAVGKKLMAGSANTVKKVSLELGGNAPCIVFDDADIDVAVKGS

LAAKFRNSGQTCVCANRILVQEGIYEKFAKAFIQAVQSLKVGNGLEESTSQGPLINEAAVQKVEKFINDATSKGANVMLG

GKRHSLGMSFYEPTVVGNVSNDMLLFREEVFGPVAPLIPFKTEEEAVHMANDTNAGLAAYIFTKSIPRSWRVSESLEYGL

VGVNEGIISTEVAPFGGVKQSGLGREGSKYGIDEYLELKYICMGNLG

>ZmALDH6B1

MLRSALFRSAPGLRRSPATAHLSTAAAAAAAWLSNGPASAPSRVRLLIGGEFVESRADEHVDVTNPATQEVVSRIPLTTA

DEFKAAVDAARTAFPGWRNTPVTTRQRVMFKFQELIRANMDKLAENITTEQGKTLKDAWGDVFRGLEVVEHACGMGTLQM

GEYVSNVSNGIDTFSIREPLGVCAGICPFNFPAMIPLWMFPIAVTCGNTFVLKPSEKDPGAAMMLAELAMEAGLPKGVLN

IVHGTNDVVNNICDDEDIKAVSFVGSNTAGMHIYSRASAAGKRVQCNMGAKNHAIILPDADRDATLNALIAAGFGAAGQR

CMALSTAVFVGGSESWEDELVKRASGLVVSSGMVNDADLGPVISRQAKDRICKLVQSGVDLCARILLDGRKIVVPYPVVY

>ZmALDH7B6

MGAFAKEEHQFLAELGLAQRNPGAFACGAWGGSGPTVTSTSPTNNQVIAEVVEASVHDYEEGMRACFDAAKTWMAIPAPK

RGEIVRQIGDALRAKLHHLGRLVSLEMGKILPEGIGEVQEIIDMCDYAVGLSRQLNGSIIPSERPNHMMMEVWNPLGVVG

VITAFNFPCAVLGWNACIALVCGNCVVWKGAPTTPLITIAMTKIVASVLEKNNLPGAIFTSFCGGTEIGQAIALDIRIPL

VSFTGSTRAGLMVQQQVSARFGKCLLELSGNNAIIVMDDADIQLAVRSVLFAAVGTAGQRCTTCRRLILHENIYQTFLDQ

LVEVYKQVRIGDPLEKGTLLGPLHTPASKENFLKGIQTIKSQGGKILFGGSAIESEGNFVQPTIVEITPSAPVVKEELFG

PVLYVMKFQSLKEAIEINNSVPQGLSSSIFTKRPDIIFKWLGPHGSDCGIVNVNIPTNGAEIGGAFGGEKATGGGREAGS

DSWKQYMRRATCTINYGSELPLAQGINFG

>ZmALDH10A5

MAPPQTIPRRGLFIGGAWREPCLGRRLPVVNPATEATIGDIPAGTAEDVEIAVAAARDAFSRDGGRHWSRAPGAVRANFL

RAIAAKIKDRKSELALLETLDSGKPLDEASGDMDDVAACFEYYADLAEALDGKQQSPISLPMENFKSYVLKEPIGVVGLI

TPWNYPLLMATWKVAPALAAGCTTILKPSELASVSCLELGAICMEIGLPPGVLNIITGLGPEAGAPLSSHSHVDKVAFTG

STETGKRIMISAAQMVKPVSLELGGKSPLIVFDDIGDIDKAVEWTMFGIFANAGQVCSATSRLLLHEKIAKKFLDRLVAW

AKNIKVSDPLEEGCRLGSVISEGQYEKIKKFISTARSEGATILYGGGRPQHLRRGFFLEPTIITDVSTSMQIWQEEVFGP

VICVKEFRTESEAVELANDTHYGLAGAVISNDQERCERISKALHSGIIWINCSQPCFVQAPWGGNKRSGFGRELGEWGLD

NYLTVKQVTKYCSDEPWGWYQPPSKL

>ZmALDH10A8

MASPAMVPLRQLFVDGEWRPPAQGRRLPVVNPTTEAHIGEIPAGTAEDVDAAVAAARAALKRNRGRDWARAPGAVRAKYL

RAIAAKVIERKPELAKLEALDCGKPYDEAAWDMDDVAGCFEYFADQAEALDKRQNSPVSLPMETFKCHLRREPIGVVGLI

TPWNYPLLMATWKIAPALAAGCTAVLKPSELASVTCLELADICKEVGLPSGVLNIVTGLGPDAGAPLSAHPDVDKVAFTG

SFETGKKIMASAAPMVKPVTLELGGKSPIVVFDDVDIDKAVEWTLFGCFWTNGQICSATSRLLIHTKIAKKFNERMVAWA

KNIKVSDPLEEGCRLGPVVSEGQYEKIKKFISNAKSQGATILTGGVRPAHLEKGFFIEPTIITDITTSMEIWREEVFGPV

LCVKEFSTEDEAIELANDTQYGLAGAVISGDRERCQRLSEEIDAGCIWVNCSQPCFCQAPWGGNKRSGFGRELGEGGIDN

YLSVKQVTEYISDEPWGWYQSPSKL

>ZmALDH10A9

MMASQAMVPLRQLFVDGEWRPPAQGRRLPVVNPTTEAHIGEIPAGTAEDVDAAVAAARAALKRNRGRDWARAPGAVRAKY

LRAIAAKVIERKQELAKLEALDCGKPYDEAAWDMDDVAGCFEYFADQAEALDKRQNSPVSLPMETFKCHLRREPIGVVGL

ITPWNYPLLMATWKVAPALAAGCAAVLKPSELASVTCLELADICKEVGLPPGVLNIVTGLGPDAGAPLSAHPDVDKVAFT

GSFETGKKIMAAAAPMVKPVTLELGGKSPIVVFDDVDIDKAVEWTLFGCFWTNGQICSATSRLLVHTKIAKEFNEKMVAW

AKNIKVSDPLEEGCRLGPVVSEGQYEKIKKFILNAKSEGATILTGGVRPAHLEKGFFIEPTIITDITTSMEIWREEVFGP

VLCVKEFSTEDEAIELANDTQYGLAGAVISGDRERCQRLSEEIDAGIIWVNCSQPCFCQAPWGGNKRSGFGRELGEGGID

NYLSVKQVTEYISDEPWGWYRSPSKL

>ZmALDH11A3

MALAGTGVFAEILDSEVYRYYADGEWRSSASGKSVAIVNPTTRKTQYRVQACTQEEVNKAMDAAKVAQKAWARTPLWKRA

ELLHKAAAILKEHKAPIAECLVKEIAKPAKDAVSEVVRSGDLVSYTAEEGVRILGEGKLLVSDSFPGNERNKYCLSSKIP

LGVVLAIPPFNYPVNLAVSKIGPALIAGNALVLKPPTQGAVAALHMVHCFHLAGFPKGLISCVTGKGSEIGDFLTMHPGV

NCISFTGGDTGIAISKKAGMVPLQMELGGKDACIVLEDADLDLVSANIVKGGFSYSGQRCTAVKVVLIMESIADAVVQKV

NAKLAKLKVGPPEDDSDITPVVTESSANFIEGLVMDAKEKGATFCQEYRREGNLIWPLLLDHVRPDMRIAWEEPFGPVLP

VIRINSVEEGIHHCNASNFGLQGCIFTRDINKAILISDAMETGTVQINSAPARGPDHFPFQGLKDSGIGSQGITNSINMM

TKVKSTVINLPSPSYTMG

>ZmALDH12A1

MSRLLSRQHLAAVRRSAPFACVSRWLHTPSFATVSPQEVSGSSPAEVQNFVQGSWTASANWNWIVDPLNGDKFIKVAEVQ

GTEIKPFVESLSKCPKHGLHNPLKAPERYLMYGDISAKAAHMLGQPAVLDFFAKLIQRVSPKSYQQALAEVQVSQKFLEN

FCGDQVRFLARSFAVPGNHLGQRSNGYRWPYGPVAIITPFNFPLEIPLLQLMGALYMGNKPVLKVDSKVSIVMEQMIRLL

HDCGLPAEDMDFINSDGAVMNKLLLEANPKMTLFTGSSRVAEKLAADLKGRVKLEDAGFDWKILGPDVQEVDYVAWVCDQ

DAYACSGQKCSAQSVLFMHKNWSSSGLLEKMKKLSERRKLEDLTIGPVLTVTTEAMIEHMNNLLKIRGSKVLFGGEPLAN

HSIPKIYGAMKPTAVFVPLEEILKSGNFELVTKEIFGPFQVVTEYSEDQLELVLEACERMNAHLTAAVVSNDPLFLQDVL

GRSVNGTTYAGIRARTTGAPQNHWFGPAGDPRGAGIGTPEAIKLVWSCHREVIYDVGPVPESWALPSAT

>ZmALDH18B1

MATADRTRTFMKDVKRVIIKVGTAVVTRGDDGRLAVGRLGCLCEQVKELNVLGYEVILVTSGAVGVGKQRLKYRKLVNSS

FADLQKPQMELDGKACAAVGQSGLMALYDMLFTQLDVSSSQLLVTDSDFENPNFRERLCETVESLLDLKVVPIFNENDAI

STRKAPYEDSSGIFWDNDSLAGLLAIELKADLLVLLSDVDGLYSGPPSEPGSKIIHTYIKDKHYSGITFGDKSRVGRGGM

TAKVKAAFVASNSGTPVVITSGFASQSIVRVLQGEKIGTLFHKDASLWEPSKDVSAREMAVAARECSRRLQNLSSDERKK

ILLDIADALEQNEDLIRTENEADVSAAQDAGYQKSLVDRLTLKPEKIASLAKSIRTLANMEDPINQILKRTEVAEDLVLE

KTSCPLGVLLIVFESRPDALVQIASLAVRSGNGLLLKGGKEAMRSNTVLHKVITGAIPDNVGQKLIGLVTSRDEIADLLK

LDDVIDLVIPRGSNKLVSQIKASTKIPVLGHADGICHVYIDKSADMNMAKRIVMDAKTDYPAACNAMETLLVHKDLIKAP

GLDDILLSLKTEGVAIYGGPVAHEVLCIPKADSFHHEYSSMACTIEFVDDVQSAINHIHRYGSAHTDCIITTDDKVAETF

LRQVDSAAVFHNASTRFSDGARFGLGAEVGISTGRIHARGPVGVEGLLTTRWIMRGSGQVVNGDKNVAYTHKNLPLQ

>ZmALDH18B2

MGRGGIGGAAAMAMAMETADPARAFVKDVKRIIIKVGTAVVTGMNGRLAMGRLGSLCEQVKQLNFQGYEVILVTSGAVGV

GRQRLQYRKLIHSSFADLQNPQMNFDGKACAAVGQSVLMAIYDTLFSQLDVTSSQLLVTDRDFKDPSFGDQLRETVFSLL

DLKVVPLFNENDAISTRRQPYEDSSGIFWDNDSLAALLAAELNADLLIMLSDVEGLYSGPPSDPQSKIIHTYVNEKHGKL

ISFGEKSSVGRGGMQAKVSAAANAASKGVPVVIASGFATDSIITVLKGEKIGTLFHNEANLWACSKEATAREMAVAARDC

SRRLQKLSSEERKQILLDIADALEANEDAIRSENDADVEAAQVAGYEKSLVARMTLKPGKITNLARSIRKTADMEDPISH

TLKRTEVAKDLVFEKAYCPLGVLLIIFESRPDALVQIASLAIRSGNGLLLKGGKEVMRSNAILHKVITGVIPDTVGKKLI

GLVTSKEEIADLLALDDVIDLVIPRGSKSLVSQIKATTKIPVLGHADGICHVYIDKSADMDMAKRIVLDAKIDYPAACNA

METLLVHKDLNKSEGLDDLLVELEKEGVVIYGGPVAHDKLKVPKVDSFRHEYSSMACTVEFVDDVQSAIDHINRYGSAHT

DCIITTDRSAAEAFLQQVDSAAVFHNASTRFCDGTRFGLGAEVGISTERIHARGPVGVDGLLTTRCILRGSGQVVNGDKG

VVYTHKDLPLQ

>ZmALDH22A1

MAFWWPLLVLAAAYALCRLLLFLIPPTVPSIDVDASDVLAKEDSFIYIPRRGKSTQTDKVQCYEPATMKYLGYFPVVTPD

EVKEHVAQSRKAQRIWAKSSFKQRRQFLRILLKYILEHQDLICEVSSRDTGKTMVDASLGEIMTTCEKITWLLDEGEKWL

KPEYRSTGRSMLHKRAKVEFYPLGVIGAIVSWNYPFHNVFNPVLAAVFSGNAAVIKVSEHATWSGCFYFRIIQAALSAVG

APENLVHIITGFAETGQALVSSVDKIIFVGSPGVGKMIMKRASETLIPVTLELGGKDSFIVCEDVDLPSVVQVATRAALQ

SSGQNCAGAERFYVHDDIYSAFVSQIVKTVKSISVGPPLSGRYDMGAICMIEHSEKLQNLVNDALDKGAEIAVRGSFGNL

GEDAVDQFFPPTVLVNVDHTMKIMQEETFGPIIPIMKFSSDEEAIKLANDSKYGLGCAVFSGNQKRAIRIASQLHCGVAA

INDFASSYMCQSLPFGGVKDSGFGRFAGVEGLRACCLVKSVVEDRLWPYIRTVIPKPIQYPVSEHGFEFQQLLVETLYGY

SVWDRLRSLVNLIKMVTEQNFAPTSNATTKKRR

Table. S7. AtALDH proteins N-glycosylation sites.

**----------------------------------------------------------------------**

**SeqName Position Potential Jury N-Glyc**

**agreement result**

**----------------------------------------------------------------------**

**AtALDH2B4 50 NPSV 0.6740 (8/9) + WARNING: PRO-X1.**

**AtALDH2B4 401 NATL 0.6011 (6/9) +**

**AtALDH2B4 458 NETK 0.6336 (9/9) ++**

**----------------------------------------------------------------------**

**---------------------------------------------------------------------**

**SeqName Position Potential Jury N-Glyc**

**agreement result**

**----------------------------------------------------------------------**

**AtALDH2C4 421 NNTK 0.5898 (8/9) +**

**----------------------------------------------------------------------**

**(Threshold=0.5)**

**----------------------------------------------------------------------**

**SeqName Position Potential Jury N-Glyc**

**agreement result**

**----------------------------------------------------------------------**

**AtALDH3F1 149 NASA 0.5993 (6/9) +**

**----------------------------------------------------------------------**

**----------------------------------------------------------------------**

**SeqName Position Potential Jury N-Glyc**

**agreement result**

**----------------------------------------------------------------------**

**AtALDH3I1 231 NTTI 0.6870 (9/9) ++**

**AtALDH3I1 274 NLTP 0.2650 (8/9) --**

**AtALDH3I1 465 NDTV 0.5169 (5/9) +**

**----------------------------------------------------------------------**

**----------------------------------------------------------------------**

**SeqName Position Potential Jury N-Glyc**

**agreement result**

**----------------------------------------------------------------------**

**AtALDH5F1 67 NKTI 0.7232 (9/9) ++**

**AtALDH5F1 176 NLSD 0.7066 (9/9) ++**

**AtALDH5F1 450 NDTI 0.4462 (6/9) -**

**----------------------------------------------------------------------**

**----------------------------------------------------------------------**

**SeqName Position Potential Jury N-Glyc**

**agreement result**

**----------------------------------------------------------------------**

**AtALDH6B2 15 NGTY 0.7224 (9/9) ++**

**AtALDH6B2 24 NPTT 0.6586 (8/9) + WARNING: PRO-X1.**

**AtALDH6B2 196 NITT 0.7474 (9/9) ++**

**AtALDH6B2 236 NVSN 0.6461 (9/9) ++**

**AtALDH6B2 316 NDTV 0.4772 (4/9) -**

**----------------------------------------------------------------------**

**----------------------------------------------------------------------**

**SeqName Position Potential Jury N-Glyc**

**agreement result**

**----------------------------------------------------------------------**

**AtALDH7B4 135 NGSV 0.6987 (9/9) ++**

**AtALDH7B4 417 NNSV 0.4807 (6/9) -**

**----------------------------------------------------------------------**

**----------------------------------------------------------------------**

**SeqName Position Potential Jury N-Glyc**

**agreement result**

**----------------------------------------------------------------------**

**AtALDH10A8 410 NDSH 0.3912 (6/9) -**

**AtALDH10A8 423 NDTE 0.5651 (5/9) +**

**AtALDH10A8 443 NCSQ 0.5043 (3/9) +**

**----------------------------------------------------------------------**

**----------------------------------------------------------------------**

**SeqName Position Potential Jury N-Glyc**

**agreement result**

**----------------------------------------------------------------------**

**AtALDH10A9 382 NVTT 0.5721 (7/9) +**

**AtALDH10A9 415 NDSQ 0.4262 (8/9) -**

**AtALDH10A9 448 NCSQ 0.5780 (6/9) +**

**----------------------------------------------------------------------**

**----------------------------------------------------------------------**

**SeqName Position Potential Jury N-Glyc**

**agreement result**

**----------------------------------------------------------------------**

**AtALDH12A1 32 NHSI 0.6784 (9/9) ++**

**AtALDH12A1 347 NWSK 0.6090 (7/9) +**

**AtALDH12A1 406 NHSI 0.3920 (8/9) -**

**AtALDH12A1 432 NKTY 0.6320 (9/9) ++**

**AtALDH12A1 492 NGTT 0.3962 (8/9) -**

**----------------------------------------------------------------------**

**----------------------------------------------------------------------**

**SeqName Position Potential Jury N-Glyc**

**agreement result**

**----------------------------------------------------------------------**

**AtALDH18B1 77 NSSF 0.5947 (9/9) ++**

**AtALDH18B1 124 NDSS 0.7233 (9/9) ++**

**AtALDH18B1 138 NETV 0.6790 (9/9) ++**

**AtALDH18B1 176 NDSL 0.6343 (8/9) +**

**AtALDH18B1 331 NVTT 0.6788 (9/9) ++**

**AtALDH18B1 501 NTTK 0.6198 (8/9) +**

**AtALDH18B1 651 NAST 0.4218 (7/9) -**

**----------------------------------------------------------------------**

**----------------------------------------------------------------------**

**SeqName Position Potential Jury N-Glyc**

**agreement result**

**----------------------------------------------------------------------**

**AtALDH18B2 77 NSSF 0.5944 (9/9) ++**

**AtALDH18B2 176 NDSL 0.5933 (7/9) +**

**AtALDH18B2 266 NISK 0.7893 (9/9) +++**

**AtALDH18B2 501 NSTK 0.5390 (5/9) +**

**AtALDH18B2 651 NAST 0.4236 (7/9) -**

**----------------------------------------------------------------------**

**----------------------------------------------------------------------**

**SeqName Position Potential Jury N-Glyc**

**agreement result**

**----------------------------------------------------------------------**

**AtALDH22A1 455 NDSR 0.4917 (6/9) -**

**AtALDH22A1 588 NVSR 0.5391 (4/9) +**

**----------------------------------------------------------------------**

Table. S8. Prediction of secondary structure of the identified AtALDH proteins.

ALDH2B4

SOPMA :

Alpha helix (Hh) : 223 is 41.45%

3_10_  helix (Gg) : 0 is 0.00%

Pi helix (Ii) : 0 is 0.00%

Beta bridge (Bb) : 0 is 0.00%

Extended strand (Ee) : 87 is 16.17%

Beta turn (Tt) : 42 is 7.81%

Bend region (Ss) : 0 is 0.00%

Random coil (Cc) : 186 is 34.57%

Ambiguous states (?) : 0 is 0.00%

Other states : 0 is 0.00%

ALDH2B7

Alpha helix (Hh) : 236 is 44.19%

3_10_  helix (Gg) : 0 is 0.00%

Pi helix (Ii) : 0 is 0.00%

Beta bridge (Bb) : 0 is 0.00%

Extended strand (Ee) : 84 is 15.73%

Beta turn (Tt) : 40 is 7.49%

Bend region (Ss) : 0 is 0.00%

Random coil (Cc) : 174 is 32.58%

Ambiguous states (?) : 0 is 0.00%

Other states : 0 is 0.00%

ALDH2C4

SOPMA :

Alpha helix (Hh) : 200 is 39.92%

3_10_  helix (Gg) : 0 is 0.00%

Pi helix (Ii) : 0 is 0.00%

Beta bridge (Bb) : 0 is 0.00%

Extended strand (Ee) : 91 is 18.16%

Beta turn (Tt) : 41 is 8.18%

Bend region (Ss) : 0 is 0.00%

Random coil (Cc) : 169 is 33.73%

Ambiguous states (?) : 0 is 0.00%

Other states : 0 is 0.00%

ALDH3F1

Alpha helix (Hh) : 217 is 44.83%

3_10_  helix (Gg) : 0 is 0.00%

Pi helix (Ii) : 0 is 0.00%

Beta bridge (Bb) : 0 is 0.00%

Extended strand (Ee) : 75 is 15.50%

Beta turn (Tt) : 34 is 7.02%

Bend region (Ss) : 0 is 0.00%

Random coil (Cc) : 158 is 32.64%

Ambiguous states (?) : 0 is 0.00%

Other states : 0 is 0.00%

ALDH3H1

Alpha helix (Hh) : 210 is 43.39%

3_10_  helix (Gg) : 0 is 0.00%

Pi helix (Ii) : 0 is 0.00%

Beta bridge (Bb) : 0 is 0.00%

Extended strand (Ee) : 80 is 16.53%

Beta turn (Tt) : 34 is 7.02%

Bend region (Ss) : 0 is 0.00%

Random coil (Cc) : 160 is 33.06%

Ambiguous states (?) : 0 is 0.00%

Other states : 0 is 0.00%

ALDH3I1

Alpha helix (Hh) : 234 is 42.55%

3_10_  helix (Gg) : 0 is 0.00%

Pi helix (Ii) : 0 is 0.00%

Beta bridge (Bb) : 0 is 0.00%

Extended strand (Ee) : 90 is 16.36%

Beta turn (Tt) : 41 is 7.45%

Bend region (Ss) : 0 is 0.00%

Random coil (Cc) : 185 is 33.64%

Ambiguous states (?) : 0 is 0.00%

Other states : 0 is 0.00%

ALDH5F1

Alpha helix (Hh) : 246 is 46.59%

3_10_  helix (Gg) : 0 is 0.00%

Pi helix (Ii) : 0 is 0.00%

Beta bridge (Bb) : 0 is 0.00%

Extended strand (Ee) : 87 is 16.48%

Beta turn (Tt) : 47 is 8.90%

Bend region (Ss) : 0 is 0.00%

Random coil (Cc) : 148 is 28.03%

Ambiguous states (?) : 0 is 0.00%

Other states : 0 is 0.00%

ALDH6B2

Alpha helix (Hh) : 203 is 33.44%

3_10_  helix (Gg) : 0 is 0.00%

Pi helix (Ii) : 0 is 0.00%

Beta bridge (Bb) : 0 is 0.00%

Extended strand (Ee) : 119 is 19.60%

Beta turn (Tt) : 48 is 7.91%

Bend region (Ss) : 0 is 0.00%

Random coil (Cc) : 237 is 39.04%

Ambiguous states (?) : 0 is 0.00%

Other states : 0 is 0.00%

ALDH7B4

Alpha helix (Hh) : 220 is 43.31%

3_10_  helix (Gg) : 0 is 0.00%

Pi helix (Ii) : 0 is 0.00%

Beta bridge (Bb) : 0 is 0.00%

Extended strand (Ee) : 88 is 17.32%

Beta turn (Tt) : 23 is 4.53%

Bend region (Ss) : 0 is 0.00%

Random coil (Cc) : 177 is 34.84%

Ambiguous states (?) : 0 is 0.00%

Other states : 0 is 0.00%

ALDH10A8

Alpha helix (Hh) : 214 is 43.15%

3_10_  helix (Gg) : 0 is 0.00%

Pi helix (Ii) : 0 is 0.00%

Beta bridge (Bb) : 0 is 0.00%

Extended strand (Ee) : 86 is 17.34%

Beta turn (Tt) : 39 is 7.86%

Bend region (Ss) : 0 is 0.00%

Random coil (Cc) : 157 is 31.65%

Ambiguous states (?) : 0 is 0.00%

Other states : 0 is 0.00%

ALDH10A9

Alpha helix (Hh) : 214 is 42.54%

3_10_  helix (Gg) : 0 is 0.00%

Pi helix (Ii) : 0 is 0.00%

Beta bridge (Bb) : 0 is 0.00%

Extended strand (Ee) : 80 is 15.90%

Beta turn (Tt) : 37 is 7.36%

Bend region (Ss) : 0 is 0.00%

Random coil (Cc) : 172 is 34.19%

Ambiguous states (?) : 0 is 0.00%

Other states : 0 is 0.00%

ALDH11A3

Alpha helix (Hh) : 207 is 41.73%

3_10_  helix (Gg) : 0 is 0.00%

Pi helix (Ii) : 0 is 0.00%

Beta bridge (Bb) : 0 is 0.00%

Extended strand (Ee) : 92 is 18.55%

Beta turn (Tt) : 34 is 6.85%

Bend region (Ss) : 0 is 0.00%

Random coil (Cc) : 163 is 32.86%

Ambiguous states (?) : 0 is 0.00%

Other states : 0 is 0.00%

ALDH12A1

Alpha helix (Hh) : 223 is 40.11%

3_10_  helix (Gg) : 0 is 0.00%

Pi helix (Ii) : 0 is 0.00%

Beta bridge (Bb) : 0 is 0.00%

Extended strand (Ee) : 75 is 13.49%

Beta turn (Tt) : 30 is 5.40%

Bend region (Ss) : 0 is 0.00%

Random coil (Cc) : 228 is 41.01%

Ambiguous states (?) : 0 is 0.00%

Other states : 0 is 0.00%

ALDH18B1

Alpha helix (Hh) : 355 is 49.51%

3_10_  helix (Gg) : 0 is 0.00%

Pi helix (Ii) : 0 is 0.00%

Beta bridge (Bb) : 0 is 0.00%

Extended strand (Ee) : 133 is 18.55%

Beta turn (Tt) : 50 is 6.97%

Bend region (Ss) : 0 is 0.00%

Random coil (Cc) : 179 is 24.97%

Ambiguous states (?) : 0 is 0.00%

Other states : 0 is 0.00%

ALDH18B2

Alpha helix (Hh) : 370 is 50.96%

3_10_  helix (Gg) : 0 is 0.00%

Pi helix (Ii) : 0 is 0.00%

Beta bridge (Bb) : 0 is 0.00%

Extended strand (Ee) : 129 is 17.77%

Beta turn (Tt) : 48 is 6.61%

Bend region (Ss) : 0 is 0.00%

Random coil (Cc) : 179 is 24.66%

Ambiguous states (?) : 0 is 0.00%

Other states : 0 is 0.00%

ALDH22A1

Alpha helix (Hh) : 261 is 43.79%

3_10_  helix (Gg) : 0 is 0.00%

Pi helix (Ii) : 0 is 0.00%

Beta bridge (Bb) : 0 is 0.00%

Extended strand (Ee) : 75 is 12.58%

Beta turn (Tt) : 38 is 6.38%

Bend region (Ss) : 0 is 0.00%

Random coil (Cc) : 222 is 37.25%

Ambiguous states (?) : 0 is 0.00%

Other states : 0 is 0.00%

Table. S9. Procheck Ramachandran plot analysis.

AtALDH2B7


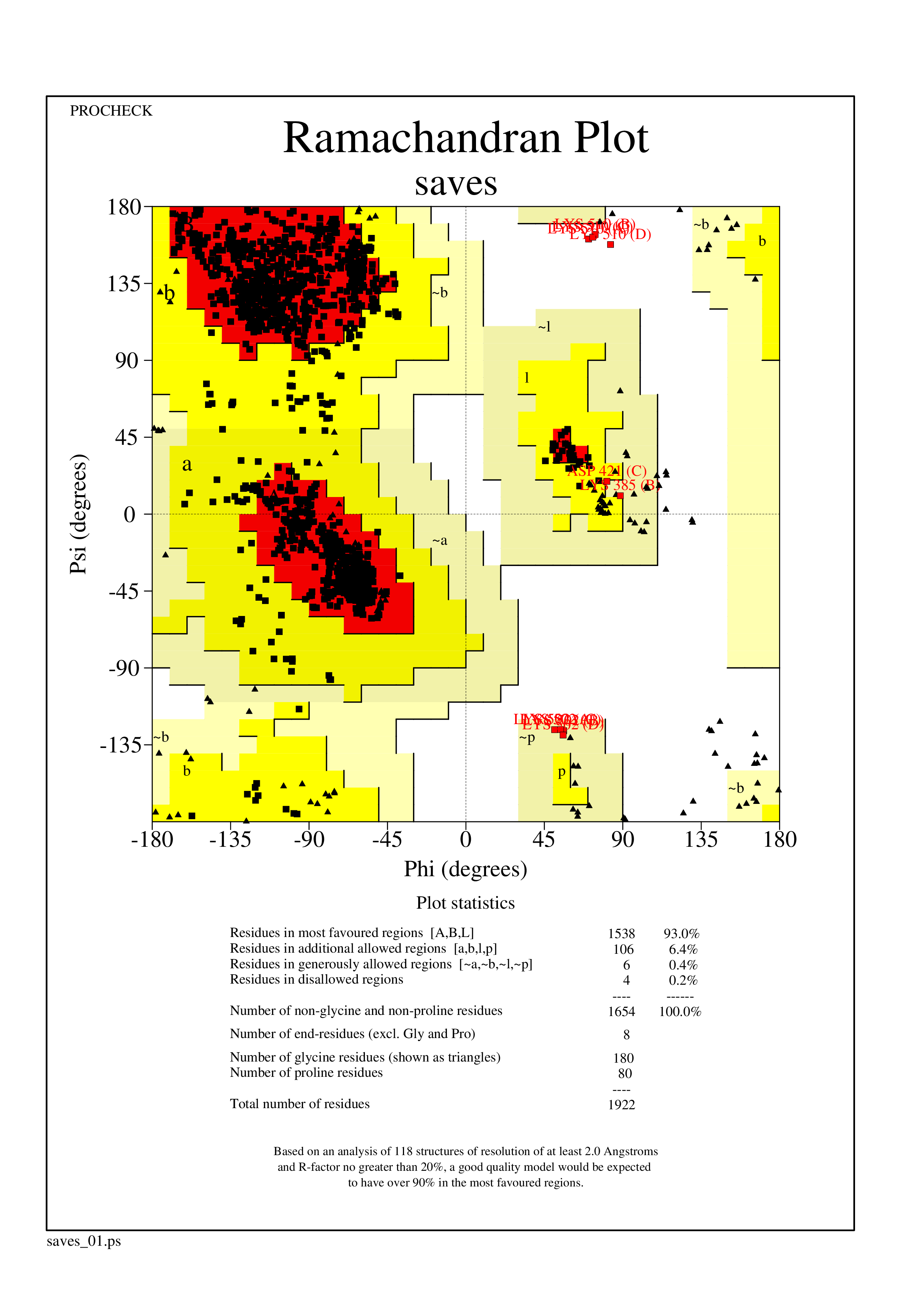


AtALDH3H1


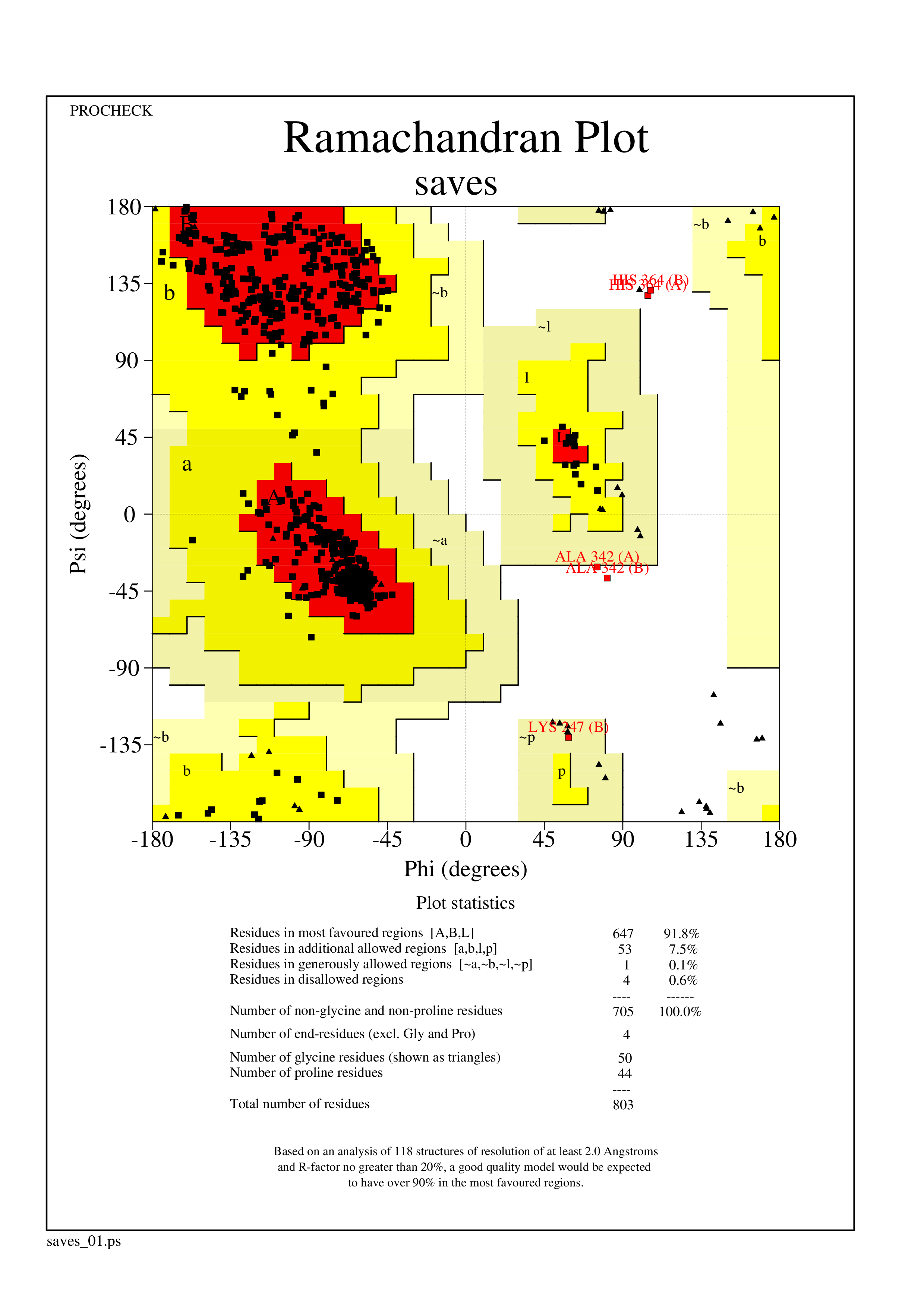


AtALDH5F1


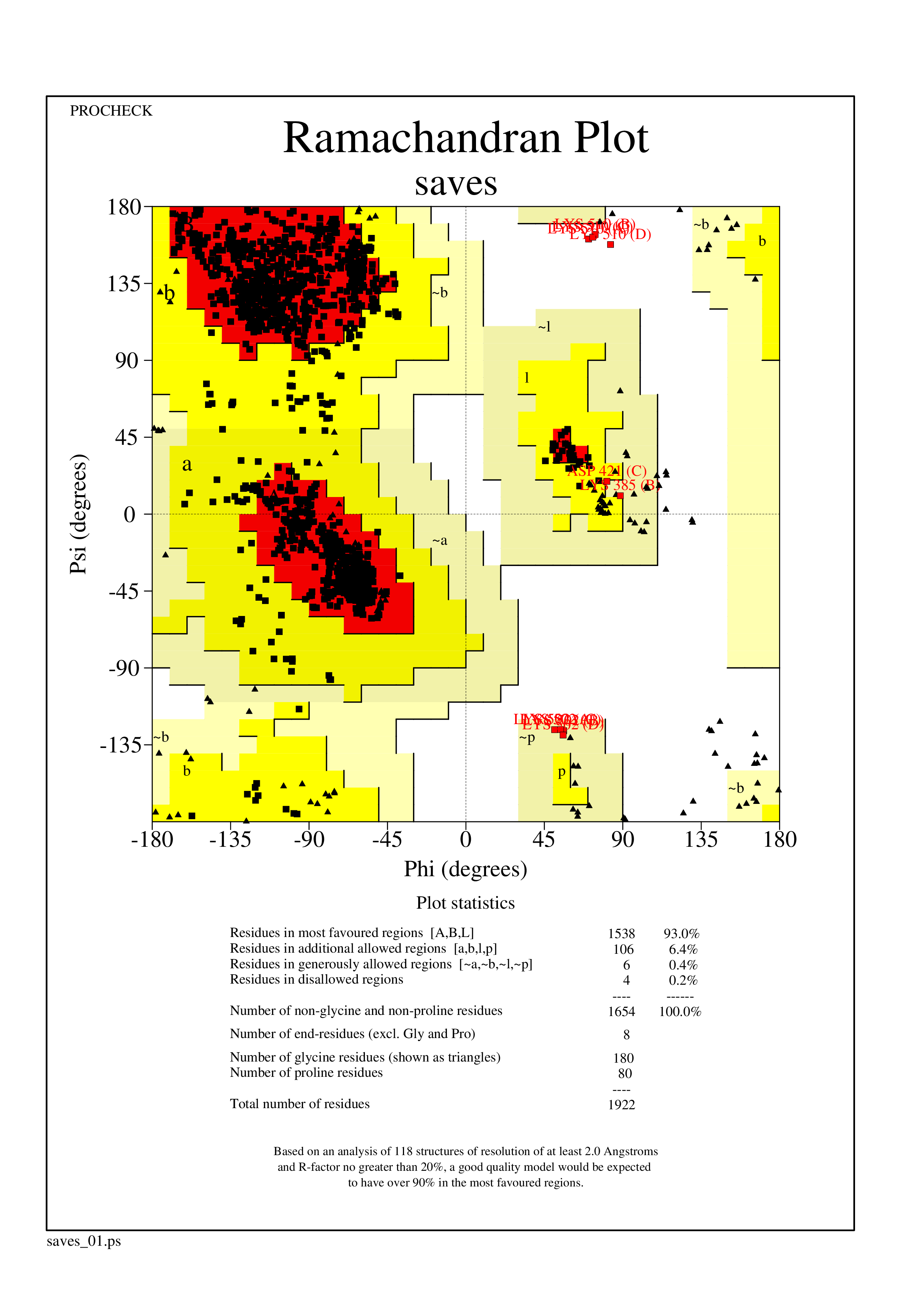

Supplement: Supplementary file 2 [file DataSheet_1.docx]
